# An Octa‐Urea [Pd_2_L_4_]^4+^ Cage that Selectively Binds to *n‐*octyl‐α‐D‐Mannoside

**DOI:** 10.1002/cphc.202100229

**Published:** 2021-05-19

**Authors:** Xander Schaapkens, Joël H. Holdener, Jens Tolboom, Eduard O. Bobylev, Joost N. H. Reek, Tiddo J. Mooibroek

**Affiliations:** ^1^ Van ‘t Hoff Institute for Molecular Sciences University of Amsterdam Science Park 904 1098 XH Amsterdam The Netherlands

**Keywords:** molecular recognition, carbohydrates, cage compounds, supramolecular chemistry, coordination compound

## Abstract

Designing compounds for the selective molecular recognition of carbohydrates is a challenging task for supramolecular chemists. Macrocyclic compounds that incorporate isophtalamide or bisurea spacers linking two aromatic moieties have proven effective for the selective recognition of all‐equatorial carbohydrates. Here, we explore the molecular recognition properties of an octa‐urea [Pd_2_L_4_]^4+^ cage complex (**4**). It was found that small anions like NO_3_^−^ and BF_4_^−^ bind inside **4** and inhibit binding of *n*‐octyl glycosides. When the large non‐coordinating anion ‘BAr^F^’ was used, **4** showed excellent selectivity towards *n*‐octyl‐α‐D‐Mannoside with binding in the order of *K*_a_≈16 M^−1^ versus non‐measurable affinities for other glycosides including *n*‐octyl‐β‐D‐Glucoside (in CH_3_CN/H_2_O 91 : 9).

## Graphical abstract

**An octa‐urea [Pd_2_L_4_]^4+^ cage complex** with non‐coordinating BAr^F^ counter ions selectively binds to *n*‐Oct‐α‐D‐Mannoside with *K*_a_=16 M^−1^ in CH_3_CN/H_2_O (91 : 9). Other carbohydrates such as *n*‐Oct‐β‐D‐Glucoside do not bind in this competitive matrix.

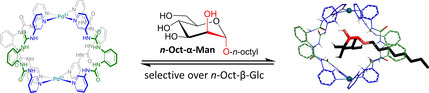

## 1 Introduction

**Figure 1:**
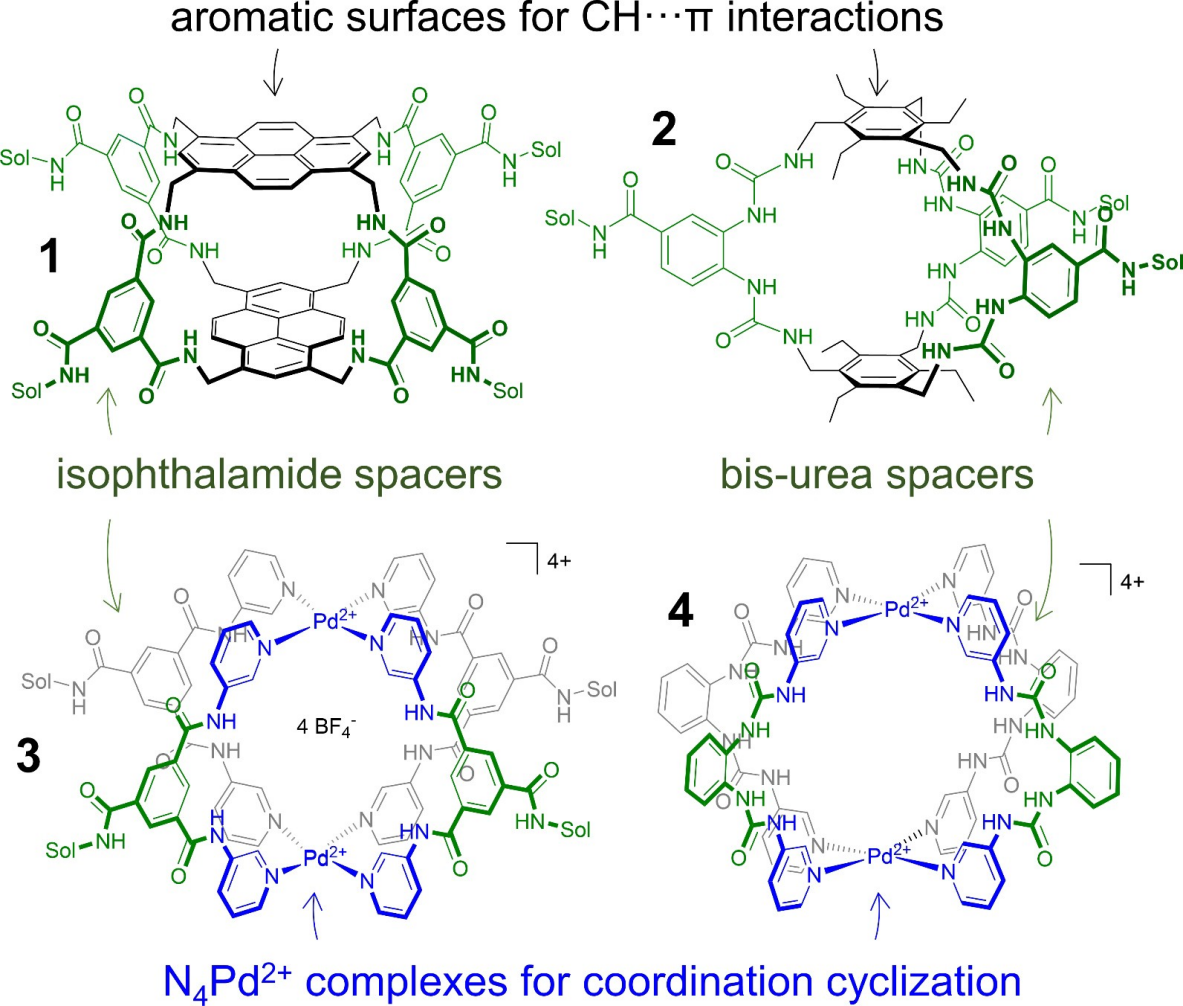
Cage designs for binding carbohydrates: previously reported covalent macrocycles **1** ^[6a]^ and **2**,^[6c]^ coordination cage **3** ^[7]^ and coordination cage **4** ^[8]^ studied in this work.

One of the most versatile class of biomolecules are carbohydrates.^[1]^ These natural molecules have been linked to various malignant phenomena such as diabetes, infection, and cancer metastasis.^[2]^ Many healthy biological processes are also mediated by carbohydrate molecules, such as: hormone activities, neuronal development, fertilization, immune surveillance and inflammatory responses.^[3]^ Glycobiology and biomedical research in general thus stand to benefit from studies to understand these processes, with the ultimate goal of unlocking novel medicinal therapies. Strategies to selectively bind carbohydrates can be seen as an essential element of such research efforts. Inspiration can be found in lectins, which are the natural class of molecules that bind carbohydrates. Crystal structures of lectin‐carbohydrate complexes reveal a large degree of interaction complementarity, where hydroxyl groups are complemented by hydrogen bonding residues in the lectin and flat CH‐surfaces of pyranoses are accommodated by aromatic residues (Phe, Tyr, Trp) for CH⋅⋅⋅π interactions.^[4]^ However, this protein sub‐group is hampered, unfortunately, by its non‐selective and low affinity binding of the target monosaccharides (typically *K*_a_∼10^2^–10^3^ M^−1^).^[5]^ The interaction complementarity has been mimicked by artificial carbohydrate binding molecules.^[6]^ Two prime examples are macrocycles **1** and **2** shown in Figure 1, which comprise pyrenyl or phenyl surfaces for CH⋅⋅⋅π interactions and polar isophthalamide or bis‐urea spacers for hydrogen bonding. It was found that **1** is selective for GlcNAc‐β‐OMe,^[6a]^ while **2** was selective for glucose (*K*_a_ of both ≈18,000 M^−1^ in water),^[6c]^ showing better selectivity and affinity than lectins. A major drawback of such covalent structures, however, is that their synthetic route requires one (or more) macrocyclization step(s) with yields rarely exceeding 20 %. These drawbacks might be remedied if the cyclization is accomplished by using reversible bonds, so that non‐productive oligomerization products can become intermediates towards the desired macrocycle.

Recently, we showed that this could be accomplished by reacting the square planar d^8^ metal Palladium (in its 2+ oxidation state) with an isophthalamide‐linked dipyridyl ligand to form **3**.^[7]^ Coordination cage **3** is shown in Figure 1 and was found to bind selectively to *n*‐oct‐β‐D‐glucoside (**5**, with *K*_a_=51 M^−1^) versus *n*‐oct‐β‐D‐galactoside (**6**, with *K*_a_=29 M^−1^) in CD_2_Cl_2_/DMSO‐*d*_6_ (9 : 1). Given the altered selectivity found for **1** and **2**, we wondered what the effect would be of replacing the isophthalamides in **3** to bis‐ureas in a structure such as **4** (see Figure 1). The nitrate version of **4** was recently published by Chand *et al*., where they were mainly interested in studying the effect of utilizing different ligand‐isomers.^[8]^ Herein, we report that octa‐urea cage **4** can host *n*‐octyl glycosides in organic media.

## 2 Results and Discussion

As is detailed in the supporting information (section S3a), attempts to utilize the [4][NO_3_^−^]_4_ complex for carbohydrate binding studies bore no fruit. The lack of binding was ascribed to firm binding of the nitrate anions within the interior of **4**, as was observed in the crystal structure of [4][NO_3_^−^]_4_.^[8]^ Moreover, the poor solubility of the nitrate version of **4** in solvents other than DMSO hampered further studies.

To enable us to the study the binding properties of **4**, the BF_4_^−^ and BAr^F^ versions were prepared by mixing the appropriate Pd^2+^ salt with the dipyridyl ligand (see section S2 for details, BAr^F^=tetrakis[3,5‐bis(trifluoromethyl)phenyl]borate). As is detailed in section S3b, synthesis of [4][BF_4_^−^]_4_ and [4][BAr^F^]_4_ in pure DMSO‐d_6_ gave complex ^1^H‐NMR spectra. These spectra were somewhat resolved at elevated temperatures (80 °C) or when adding a glycoside, thus hinting at the capacity of **4** to bind carbohydrates. However, the complexity of the spectra during titration experiments hampered a firm characterization of binding in DMSO‐d_6_.

**Figure 2:**
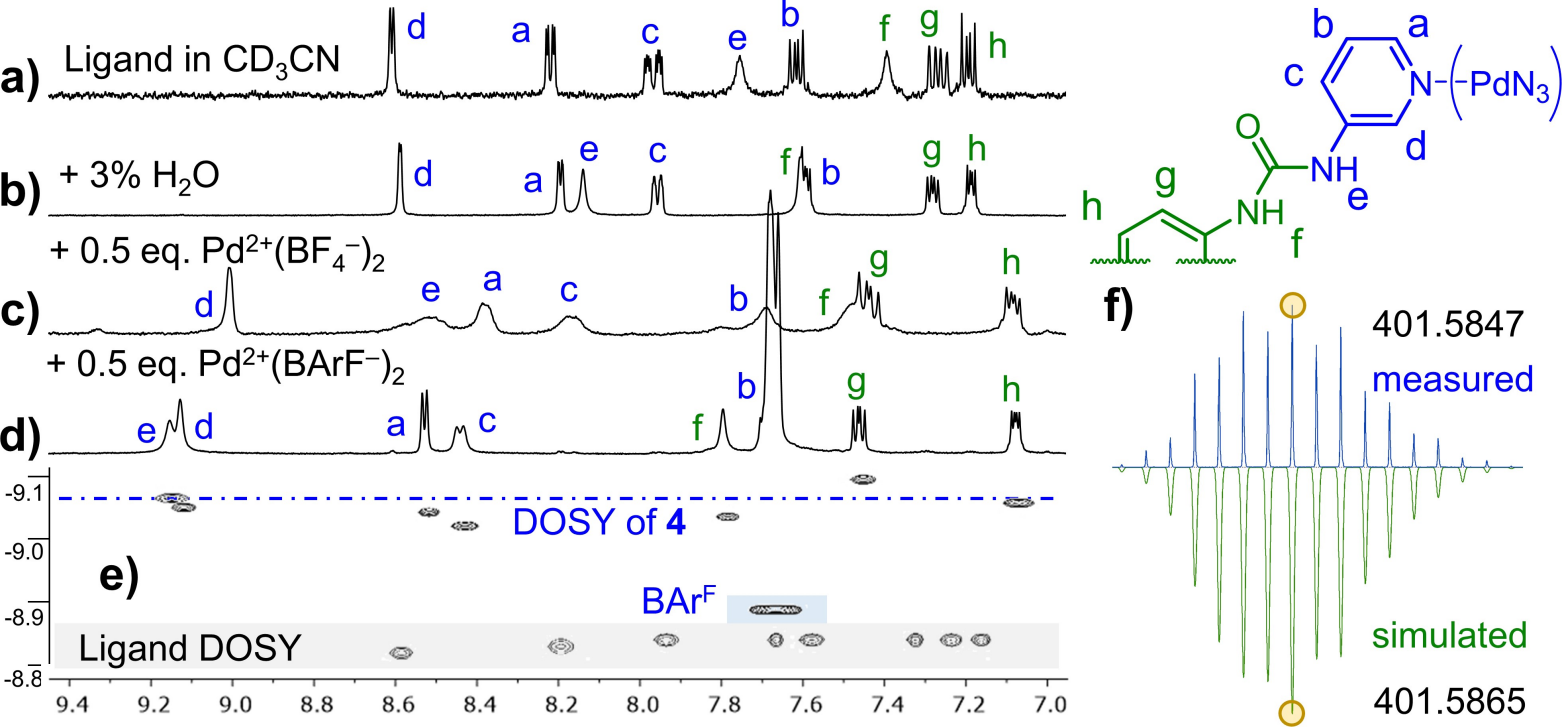
Formation and characterization of [4][BF_4_^−^]_4_ and [4][BAr^F^]_4_. a, b) Comparison of ligand in CD_3_CN+3 % H_2_O; c, d) with the addition of 0.5 eq. [Pd(MeCN)_4_][BF_4_^−^]_2_ or 0.5 eq. [Pd(MeCN)_4_][BAr^F^]_2_. e) DOSY NMR comparison of ligand and [4][(BAr^F^)_4_]. f) Measured (top) and simulated (bottom) CSI HRMS isotope distribution of [4]^4+^ with indicated highest isotopic mass of *m*/*z*=401.585 (see also Figure S2–22).

Changing the solvent from DMSO‐d_6_ to CD_3_CN with a few percentages of water resulted in ^1^H‐NMR spectra with one clear major species for both [4][BF_4_^−^]_4_ and [4][BAr^F^]_4_. This is illustrated in Figure 2, showing assigned ^1^H‐NMR spectra of the dipyridyl ligand in pure acetonitrile (a) and in 3 % water in acetonitrile (b) to which 0.5 eq. of the appropriate palladium salt was added (either BF_4_^−^ in c, or BAr^F^ in d).

The large downfield shift of protons such as **a** (8.19→8.38 or 8.53), **c** (7.95→8.17 or 8.44), **d** (8.59→9.00 or 9.12), and **e** (8.14→8.51 or 9.15) for [4][BF_4_^−^]_4_ and [4][BAr^F^]_4_ respectively, are highly indicative for pyridyl‐Pd coordination. DOSY NMR of the BAr^F^ version of **4** (Figure 2e) revealed that the diffusion constants (*D*) of the major species is smaller for the ligand (log(*D*)=−8.82) than for [4][BAr^F^]_4_ (log(*D*)=−9.04), which is also in line with complex formation. Moreover, the diffusion constant measured for the BAr^F^ anion in [4][BAr^F^]_4_ of log(*D*)=−8.89 is significantly less than that of **4**, implying that these anions are largely dissociated. For the [4][(BF_4_^−^)_4_] complex on the other hand, a {^1^H−^19^F}‐HOESY spectrum revealed a clear nOe signal between CH proton **d** and BF_4_^−^, thus showing this anion is bound to the interior of **4** (see Figure S2‐11). Lastly, the tetra‐cationic **4** was measured by cold‐spray ionization mass spectroscopy of [4][(BF_4_)_4_] and [4][(BAr^F^)_4_] solutions. The measured isotope distribution and highest monoisotopic mass (*m/z*=401.585) are in agreement with the 2 : 4 Pd : ligand ratio expected for **4** (see also Figure 2f).

To probe the possible binding properties of **4**, various binding studies were conducted with carbohydrates **5**–**12** listed in Table 1, as well as with the aromatic dimethyl terephthalate (**13**). We opted for the BAr^F^ version of **4** because of the previously noted interior binding of BF_4_^−^ evidenced by a {^1^H−^19^F}‐HOESY experiment (see section S2).

**Table 1:** Overview of binding studies performed with [4][BAr^F^]_4_ and the structures of titrants **5**–**13** with axial groups highlighted in red.

Entry	Guest	Final concentration of guest [in mM]^[a]^	[%] H_2_O in CD_3_CN	*K*_a_ [M^−1^]
1	**5**	140	3	9^[b]^
2	‘’	140	9	–^[c]^
3	**6**	58	9	<3^[d,e]^
4	**7**	139	9	16^[b]^
5	**8**	39	9	<3^[d]^
6	**9**	6	9	<3^[d]^
7	**10**	6	9	–^[c]^
8	**11**	141	3	<3^[d]^
9	**12**	28	3	–^[c]^
10	**13**	74	3	–^[c]^
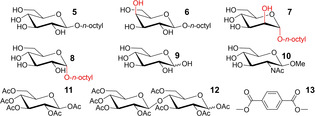				

[a] the final concentration was limited by the solubility of the titrants; [b] selective 1D NOESY by NMR irradiating [4][BAr^F^]_4_ resonances at the final concentrations showed clear nOe signals of the inward facing hydrogens with the carbohydrate region. In all titrations, there was a small and near‐linear shifts at the beginning of the titration that prevented fitting the data to a simple 1 : 1 model. Assuming a 1 : 2 model and accounting for the small shifts by fixing the ‘first’ event at 6 M^−1^ remedied this issue and the ‘second’ event then gives the actual 1 : 1 binding constant reported that is at the basis of the large peak shifting; [c] unable to fit data to a binding constant (shifts are too small and unreliable); [d] could be fitted to a 1 : 2 stoichiometry with stepwise association constants of about 2, but the shifts were very small and no saturation was achieved according to the model. Hence, these are reported as ‘likely below *K*_a_=3 M^−1^’ (which is about the detection limit of a binding constant determination with the used concentrations of host and guest); [e] 1D NOESY NMR by irradiating [4][BAr^F^]_4_ resonances at the final concentrations showed no nOe signals of the inward facing hydrogens with the carbohydrate region.

As several ^1^H‐NMR spectra of [4][BAr^F^]_4_ between 0.560 to 0.245 mM (Figure S3‐3) showed that the resonances of the cage were unperturbed, any significant self‐association of **4** could be excluded in this concentration range. Due to solubility issues, acetonitrile was used with a water contents of 3 or 9 %, depending on the solubility of the titrant. In nearly all titration experiments, most signals shifted somewhat up‐ or downfield, except for proton **g** and particularly proton **h** (see Figures S3‐4 to S3‐13 for all binding studies). Such proton dependent shifts are highly suggestive of a binding event. However, these shifts were mostly very small and nearly linear when plotted *vs* the total guest concentration (i. e., no saturation was observed). This can be ascribed to very weak binding near the detection limit of about *K*_a_≤3 M^−1^, and in some cases saturation might not have been reached due to solubility limitations (e. g. in the case of D‐Glc and Me‐β‐D−GlcNAc). Uncommonly, binding with the flat aromatic dimethyl terephthalate (**13**) was also too low to be properly quantified. Flat aromatics typically do bind strongly to the interior of covalent cages such as **1**.

**Figure 3:**
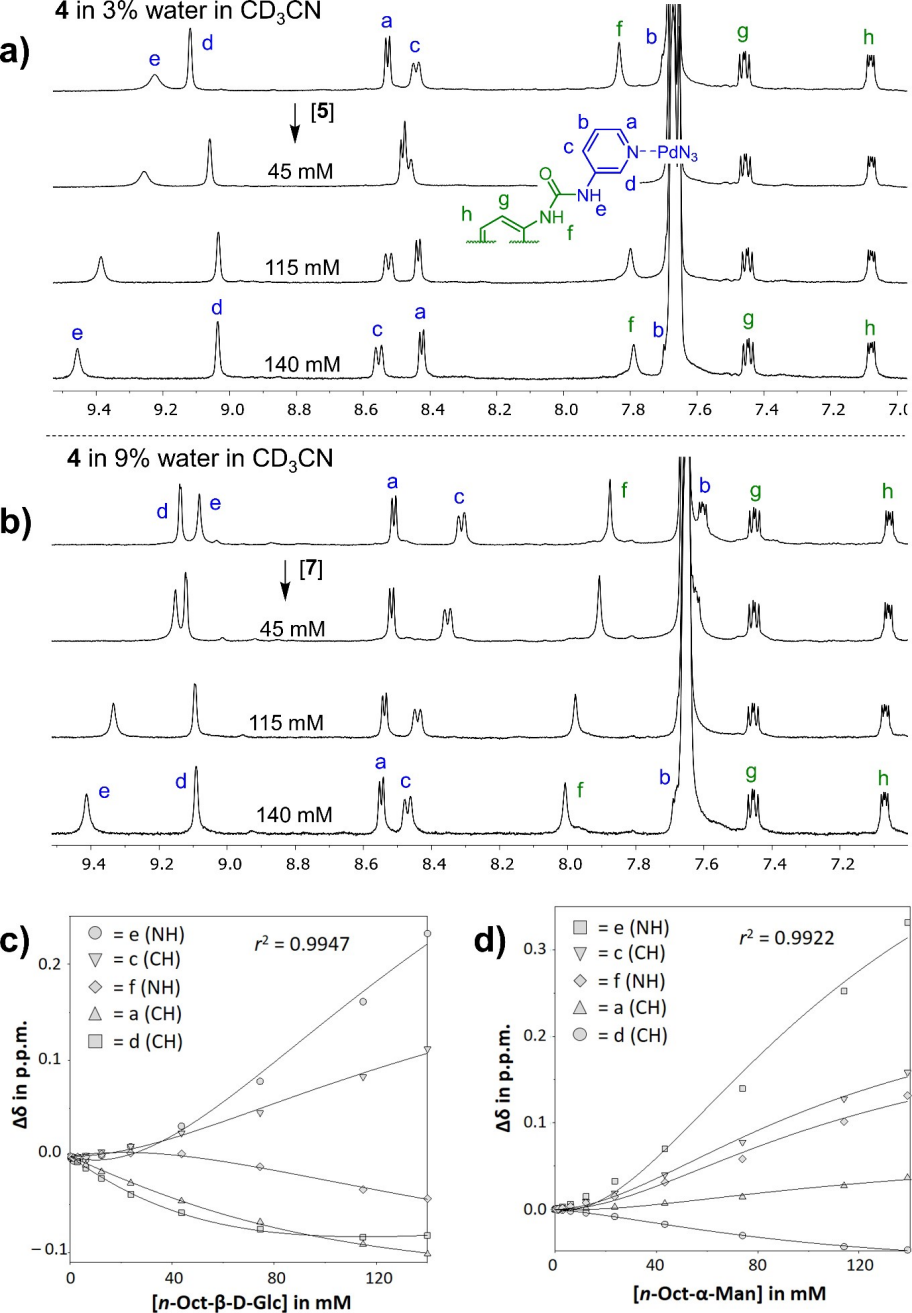
Partial ^1^H‐NMR spectra and HypNMR curve fitting analysis of a) [4][BAr^F^]_4_ titrated with glucoside **5** in acetonitrile with 3 % water and b) titrated with mannoside **7** in acetonitrile with 9 % water. c+d) Fitting on protons **a** and **c**–**f** gave a binding constant of 9 M^−1^ for **5** (c, in 3 % H_2_O in CD_3_CN) and 16 M^−1^ for **7** (d, in 9 % H_2_O in CD_3_CN) with the indicated goodness of fit (*r* ^2^) calculated over all 55 data points (see note b of Table 1 and main text for details). See also Figure S3‐4 and S3‐7.

The titration experiments of [4][BAr^F^]_4_ with *n*‐octyl‐β‐D‐glucoside **5** in CD_3_CN with *3 % H_2_O* and with *n*‐octyl‐β‐D‐mannoside **7** in CD_3_CN with *9 % H_2_O* appeared markedly different compared to the others and selected spectra are shown in Figure 3a and b respectively. With increasing concentration of **5**, most resonances of **4** shifted significantly. Notably, the inwards facing NH proton **e** shifted downfield and the inwards pointing CH proton **d** shifted upfield, while the outwards facing **g** and **h** remained nearly stationary.

In order to quantify the observed shifts in terms of a binding constant for **5** and **7**, the shifts were initially fitted to a simple 1 : 1 binding model. This model did not fit very well, in particular at the beginning of the titrations, at low concentrations of carbohydrate. This was ascribed to small near‐linear shifts, often in opposite direction of the main shifts, which were also present in many titrations with the other substrates. This phenomenon cannot be cage aggregation, as the dilution study did not reveal such shifts (see Figure S3‐3). One might speculate that carbohydrates can also be very loosely associated with the cage's exterior, leading to higher stoichiometries with small shifts. Alternatively, the small initial shifts might result from changes in the equilibrium composition of the cage's conformers and/or coordination oligomers. This phenomenon notwithstanding, the data could be fitted with reasonable accuracy as is shown in Figures 3c and d (*r* ^2^>0.99 over all 55 data points). In these fits, the initial small shifts were taken into account by using a 1 : 2 model and fixing the ‘first’ event to 6 M^−1^. This gave the reported values of an assumed 1 : 1 binding with *K*_a_=9 M^−1^ for **5** (in 3 % H_2_O in CD_3_CN) and 16 M^−1^ for **7** (in 9 % H_2_O in CD_3_CN, see also Table 1). The order of magnitude of these values is consistent with the saturation observed with the concentration of guest used (up to 140 mM). When the titration with glucoside **5** was repeated in acetonitrile with 9 % water, no significant peak shifting was observed (entry 2 in Table 1, see also Figure S3‐5). This implies a clear preference of **4** for mannoside **7** over glucoside **5**.

**Figure 4:**
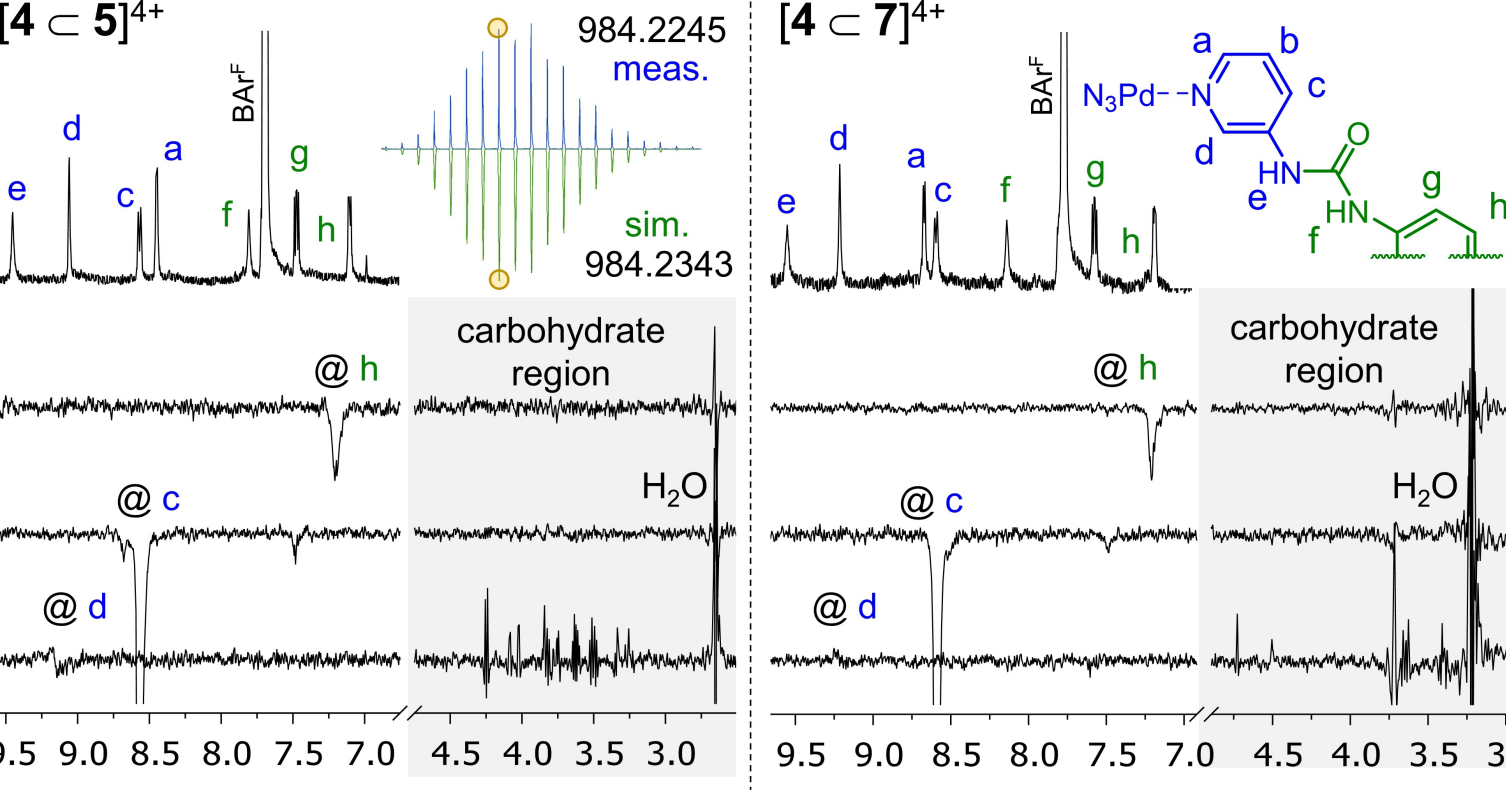
Partial ^1^H‐NMR spectrum of [**4**⊂**5**][BAr^F^]_4_ and [**4**⊂**7**][BAr^F^]_4_ and selective 1D nOe's with *t*_m_=500 ms. The top left inset figure displays the CSI HRMS isotope distribution of a [**4**⊂**5**] species as measured (blue, top) and simulated (green, bottom) with *m*/*z*=984.225 for [**4**⊂**5**][Cl_2_]^2+^; the chloride anion must originate from the eluent used in the spectrometer.

To verify if the observed shifts were indeed caused by binding of glycosides **5** and **7**, a series of selective 1D nOe spectra were measured of the final titration solutions. As is exemplified in Figure 4 for both glycosides, when proton **d** was irradiated, large signals were observed in the carbohydrate region (3.0–4.5 p.p.m.). In contrast, irradiation of the outward pointing pyridyl proton **c**, or phenyl proton **h** did not result in such large nOe signals in the carbohydrate region. These nOe data thus provide evidence that binding occurred and that **5** and **7** reside within the cage's interior. Lastly, the final titration solution of [4][BAr^F^]_4_ with glucosides **5** was investigated with cryospray ionization high resolution mass spectrometry (CSI‐HRMS). As is shown in the top‐left inset figure of Figure 4, a species was observed with a mass and isotope distribution consistent with a 1 : 1 stoichiometry of a [**4**⊂**5**]^4+^ (see also Table S3‐2).

**Figure 5:**
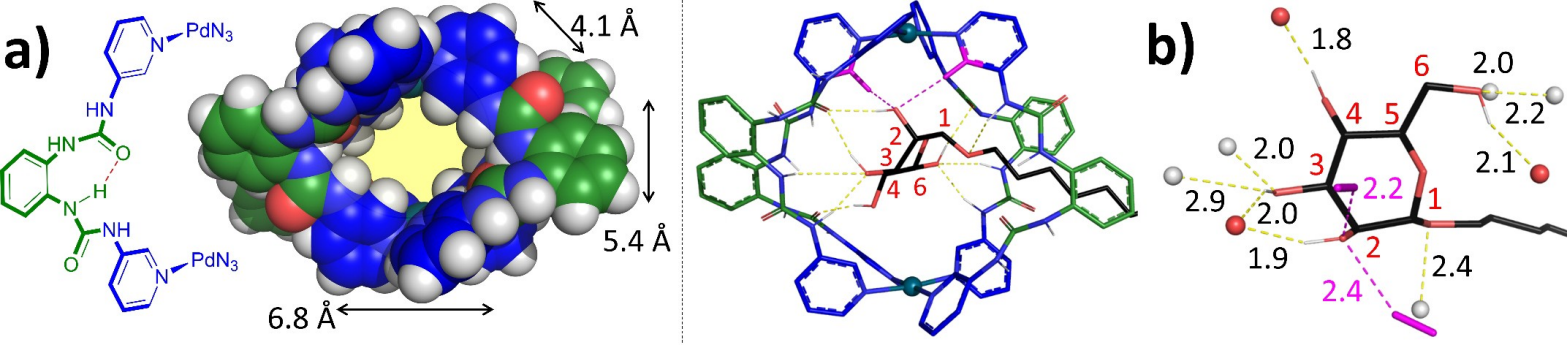
DFT optimized (ωB97X‐D/6‐31G*) molecular models of: a) the empty [4]^4+^ cage represented in space filling mode. In each bis‐pyridyl ligand, the urea's form an intramolecular hydrogen bond as shown. Some dimensions are given as well. b) [**4**⊂**7**]^4+^ with a magnification of the mannoside and the H‐bonds found in the model (yellow dashed lines). See section S4 of the supporting information for details.

While the exact molecular geometry of **4** bound to glycosides **5** and **7** could not be measured, molecular modeling was used to obtain likely approximate geometries. Details of the approach can be found in the supporting information, section S4. The energy minimum conformer of unbound **4** was approximated by the model shown in Figure 5a. This model indicates that the two urea moieties of each dipyridyl ligand establish an intramolecular hydrogen bond (also shown as red dashed line in the schematic representation). Moreover, the interior of this model has the indicated estimated dimensions, which are generally congruent with those of a carbohydrate (see Figure S4‐2).

Starting from this presumed energy minimum conformer, models of **4** bound to *n*‐octyl glycosides **5**–**8** were generated by conformational searches and DFT geometry optimizations as detailed in section S4b. As an example, the energy minimum found for [**4**⊂**7**]^4+^ is shown in Figure 5b. Interestingly, in this structure (as well as the others) the carbohydrate pyranose ring plane is not coplanar with the N_4_Pd^2+^ planes. In the case of [**4**⊂**7**]^4+^ these angles are about 40°. As can be seen in the right‐hand side of Figure 5b, the mannoside in the model is held in place by a total of nine traditional hydrogen bonding interactions involving the cage's urea groups. Six of these hydrogen bonding distance can be seen as typical for charge neutral hydrogen bonds (H⋅⋅⋅O=1.5–2.2 Å) while three can be seen as weak (H⋅⋅⋅O>2.2 Å).^[9]^ Interestingly, the axial hydroxyl on C‐2 is involved in one of the shortest hydrogen bonding H⋅⋅⋅O distances of 1.9 Å. Additionally (highlighted in pink), this axial OH‐2 is involved in two charge assisted [C−H]^δ+^⋅⋅⋅O interactions involving two of the inwards pointing pyridyl CH's (**d**) that are trans‐coordinated relative to each other. The H⋅⋅⋅O distances of 2.42 Å and 2.20 Å can be seen as weak and moderate respectively. The possibility to establish three relatively strong hydrogen bonding interactions with the axial hydroxyl of **7** provides a rationalization to the observed selectivity for this carbohydrate over glucoside **5** (where only seven hydrogen bonds were found, none involving the pyridyl CH's).

## 3 Conclusions

The nitrate version of **4** reported by Chand *et al*. that was only soluble in DMSO‐d_6_ could be modified to the BF_4_^−^ and BAr^F^ analogues which were also soluble in wet (3 % or 9 % water) CD_3_CN. Binding studies of **4**[BAr^F^]_4_ with **5**–**13** showed that binding could only be quantified for glucoside **5** in 3 % water in acetonitrile, and for mannoside **7** in 9 % water in acetonitrile. In both cases a 1 : 2 stoichiometry had to be assumed for a proper fit due to initial shifting of some peaks that we consider to be an artifact. The 1 : 1 binding constants we found are 9 M^−1^ for **5** and 16 M^−1^ for **7** and binding to the interior of **4** could be verified in both cases by selective 1D‐nOe studies. As no affinity of **4** for **5** was observed in 9 % water in acetonitrile, these data indicate a clear preference of **4** for the diaxial *n*‐octyl‐α‐mannoside **7**. This selectivity could be rationalized based on molecular modeling of [**4**⊂**7**]^4+^ where several clear hydrogen bonds were found involving the axial hydroxyl of **7**, including charge assisted [C−H]^δ+^⋅⋅⋅O interactions that were absent in a model of [**4**⊂**5**]^4+^. While the affinities found are low, these studies do show that carbohydrate binding with **4** is possible in very competitive media such as wet acetonitrile.^[10]^ Moreover, the first reported covalently‐assembled cage (a biphenyl analogue of **1**) for carbohydrate binding in a competitive medium has an affinity of merely 4.6 M^−1^ for D‐glucose in water. As such, one can actually consider the affinities in the order of 9–16 M^−1^ observed with **4** as a significant first step. We foresee that installation of a solubility group on the phenyl moiety in the dipyridyl ligand will open up further explorations of the binding potential of the octa‐urea **4** in other solvents.

## Conflict of interest

The authors declare no conflict of interest.

## Supporting information

As a service to our authors and readers, this journal provides supporting information supplied by the authors. Such materials are peer reviewed and may be re‐organized for online delivery, but are not copy‐edited or typeset. Technical support issues arising from supporting information (other than missing files) should be addressed to the authors.

Supplementary
