# Supplementary material for: An Octa‐Urea [Pd2L4]4+ Cage that Selectively Binds to n‐octyl‐α‐D‐Mannoside
Source: Chemphyschem. 2021 May 19;22(12):1187–92. doi: 10.1002/cphc.202100229 (PMC8252426; doi:10.1002/cphc.202100229)
Supplement: Supplementary file 1 — Supplementary [file CPHC-22-1187-s001.pdf]

ChemPhysChem

Supporting Information

An Octa-Urea $[Pd_2L_4]^{4+}$ Cage that Selectively Binds to *n*-octyl-α-D-Mannoside

Xander Schaapkens, Joël H. Holdener, Jens Tolboom, Eduard O. Bobylev, Joost N. H. Reek, and Tiddo J. Mooibroek*

Supplementary material

Table of contents:

Section S1. Materials and methods **2**

Section S2. Syntheses and characterizations **3**

Section S3. NMR studies of 4 **21**

Section S3a. Studies of [**4**][NO_3^-]$_4$ 21

Section S3b. Studies of [**4**][BF_4^-]$_4$ and [**4**][BAr^F]$_4$ in DMSO-d_6 22

Section S3c. Titration procedures with the formation procedures of [**4**][BAr^F]$_4$ in CD_3CN with 3 or 9% water 23

Section S3d. Dilution study of [**4**][BAr^F]$_4$ in CD_3CN with 3% water 24

Section S3e. Studies of the binding of [**4**][BAr^F]$_4$ in CD_3CN with 3 or 9% H_2O 25

Section S4. Modeling of 4 and its *n*-octyl-glycoside complexes 37

Section S4a. Unbound cage **4** 37

Section S4b. Cage **4** in complex with carbohydrates 40

Section S4c. Cartesian coordinates of models computed with DFT 42

References 51

Section S1. Materials and methods

All solvents and chemical were purchased from commercial suppliers and used without further purification. Solvents, when needed, were dried by standard distillation techniques or for larger amounts (>10 mL), the solvent was tapped from a Solvent Purification System (SPS) from mbraun (MB SPS-800, with standard mbraun drying columns). Reactions were carried out under a Nitrogen atmosphere using standard Schlenk techniques, where dry solvents are specified. The dipyridyl ligand 1,1'-(1,2-phenylene)bis(3-(pyridin-3-yl)urea) was prepared according to literature.[1] tetrakis[3,5-bis(trifluoromethyl)phenyl]borate is abbreviated as BAr^F in the text.

Nuclear Magnetic Resonance (NMR) spectra were recorded on a Bruker DRX 500 (125.72 MHz for ^{13}C), Bruker AMX 400 (100.62 MHz for ^{13}C, 128.38 MHz for ^{11}B or 376.50 MHz for ^{19}F), Bruker DRX 300 (75.48 MHz for ^{13}C) or on a Varian Mercury 300 (75.48 MHz for ^{13}C, 96.29 MHz for ^{11}B or 282.32 MHz for ^{19}F) spectrometer at room temperature or specifically specified otherwise. The residual solvent peaks were used as internal standards (^{1}H: δ 7.26 p.p.m., ^{13}C{^{1}H}: δ 77.16 p.p.m. for $CDCl_3$; ^{1}H: δ 5.32 p.p.m., ^{13}C{^{1}H}: δ 53.84 p.p.m. for CD_2Cl_2; ^{1}H: δ 2.50 p.p.m., ^{13}C{^{1}H}: δ 39.52 p.p.m. for DMSO-d_6; ^{1}H: δ 1.94 p.p.m., ^{13}C{^{1}H}: δ 1.32 p.p.m. for CD_3CN), while ^{11}B spectra were externally referenced to 15% $BF_3{\cdot}OEt_2$ in $CDCl_3$ (0.00 p.p.m.) and ^{19}F NMR spectra were externally referenced to CF_3COOH (–76.55 p.p.m.). Chemical shifts (*δ*) are given in parts per million (p.p.m.) and coupling constants (*J*) are quoted in hertz (Hz). Resonances are described as singlet (s), doublet (d), triplet (t), quartet (q), quintet (quint), a combination thereof, broad singlet (br s) or multiplet (m). Low resolution mass spectra were recorded on an Advion (T)LC-MS expression^L CMS mass spectrometer (with a TLC plate express and isocratic pump), using the Electron Spray Ionization (ESI) method. High Resolution Mass Spectra (HRMS) were recorded on a JEOL AccuTOFC-plus JMS-T100LP mass spectrometer, with the indicated ionization method.

CryoSpray Ionization High Resolution Mass Spectrometry (CSI HRMS) was conducted on a HR-ToF Bruker Daltonik GmbH (Bremen, Germany) Impact II, an ESI-ToF MS capable of resolution of at least 40000 FWHM, which was coupled to a Bruker cryo-spray unit. Detection was in positive-ion mode and the source voltage was between 4 and 6 kV. The sample was introduced with a syringe pump at a flow rate of 18 ul/hr. The drying gas (N2) was held at -35 °C and the spray gas was held at -40 °C. The machine was calibrated prior to every experiment via direct infusion of a TFA-Na solution, which provided a *m/z* range of singly charged peaks up to 3500 Da in both ion modes. Software acquisition with Compass 2.0 for Otof series and software processing with Compass DataAnalysis 4.0 sri (x64) (both Bruker Daltonik GmbH (Bremen, Germany)). The peak with the highest intensity of an isotope distribution was used to report and calculate the difference between measured and calculated values (Δ, expressed in p.p.m.). These reported values were obtained from Bruker DataAnalysis 4.4 software, wherein the positive ionization is taken into account by subtracting the mass of the missing electrons (0.0005486 Da per electron). NB: the isotope distributions were simulated with mMass software, which does not take into account the mass of the missing electrons in positive ions. This leads to apparent mass differences as large as 0.0021944 Da (4 x 0.0005486) in a 4+ species.

^{1}H NMR titrations were performed using a Bruker DRX 500 spectrometer operating at 298 K or specifically specified otherwise. The titrations were performed using one of the described procedures in **Section S3c**. Association constants (K_a) were determined by monitoring the change in chemical shift (Δδ) for a selected proton resonance of **4** and fitting these shifts to a 1:2 binding model using HypNMR.[2] An estimated goodness of fit was calculated as r^2 from all the observed and fitted Δδ values used in HypNMR.

Section S2. Syntheses and characterizations

Comments on the synthesis and studies of 4 with different anions

The original nitrate version of **4** was readily prepared using the literature procedure.[1] [**4**][BF_4^-]$_4$ was equally readily prepared from the commercially available tetrakis(acetonitrile)palladium tetrafluoroborate [Pd(NCMe)$_4$]$^{2+}$][BF_4^-]$_4$. [**4**][BAr^F]$_4$ on the other hand, was more tedious to obtain because a Pd(II)(BAr^F)$_2$ complex is not commercially available. Moreover, synthesis of [**4**][BAr^F]$_4$] by mixing appropriate amounts of the ligand, bis(acetonitrile)dichloropalladium(II) and sodium BAr^F might give [**4**][(BAr^F)$_4$] together with four equivalents of sodium chloride; Cl^- would likely compete with the pyridyl ligands for Pd coordination and/or compete for binding with the cage's interior. Therefore, a ((tetrakis)acetonitrile)palladium(II) BAr^F precursor was synthesized *via* counter ion exchange in a biphasic mixture of water and dichloromethane (DCM), as detailed below.

The ^{1}H-NMR spectra of **4** with BF_4^- or BAr^F counter ions clearly show a major species that could be fully assigned, and mass spectroscopic analysis of both complexes revealed a clear prevalence of the 2:4 Pd:ligand stoichiometry of **4**. Interestingly, the DOSY spectra of the BF_4^- complex indicated an average diffusion constant (D) of log(D) = - 8.85 (see Figure S2-10), which is only slightly larger than that of the ligand of log(D) = -8.82 (see Figure S2-2). As highlighted in the main text, this value was much large for **4** in the BAr^F version (log(D) = -9.04) with the BAr^F anion itself at log(D) = -8.89 (see Figure S2-20, thus clearly not bound to **4**).

Our hypothesis for the similar diffusion constants observed by DOSY for the ligand and the BF_4^- version of **4** is the di-urea ligand self-aggregates in acetonitrile with 3% water. Indeed, we observed that the ligand could not be dissolved when higher concentration samples were prepared. Moreover, **4** might well be smaller in the BF_4^- version than in the BAr^F version due to binding of BF_4^- in the interior of **4**. This binding was verified with the {^{1}H-^{19}F}-HOESY spectrum shown in Figure S2-11, showing a clear nOe signal between NH proton **d** and BF_4^-.

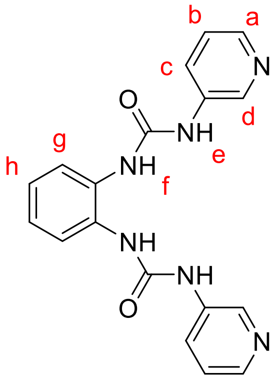

1,1'-(1,2-phenylene)bis(3-(pyridin-3-yl)urea) was synthesized according to literature.[1]
^{1}H NMR (500 MHz, CD_3CN): δ 8.60 (d, J = 2.2 Hz, 2H, H_d), 8.22 (dd, J = 4.7, 1.5 Hz, 2H, H_a), 7.96 (ddd, J = 8.4, 2.7, 1.5 Hz, 2H, H_c), 7.75 (s, 2H, H_e), 7.61 (dd, J = 6.0, 3.6 Hz, 2H, H_b), 7.39 (s, 2H, H_f), 7.27 (dd, J = 8.4, 4.7 Hz, 2H, H_g), 7.19 (dd, J = 6.1, 3.6 Hz, 2H, H_h). ^{1}H 2D-DOSY (500 MHz, CD_3CN): log (D) = -8.81 m^2/s, with d20 = 0.12 sec and p30 = 500 µsec.

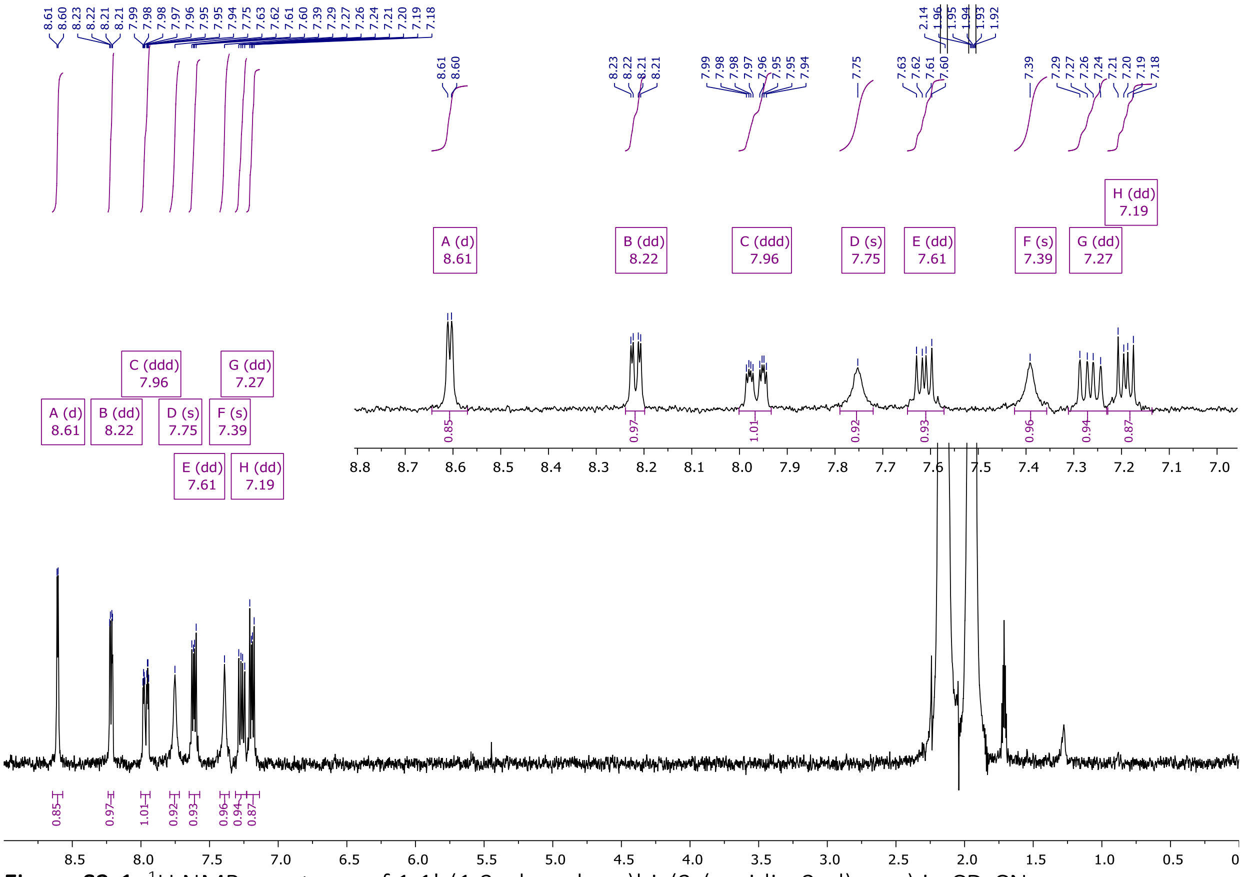

Figure S2-1. ^{1}H NMR spectrum of 1,1'-(1,2-phenylene)bis(3-(pyridin-3-yl)urea) in CD_3CN.

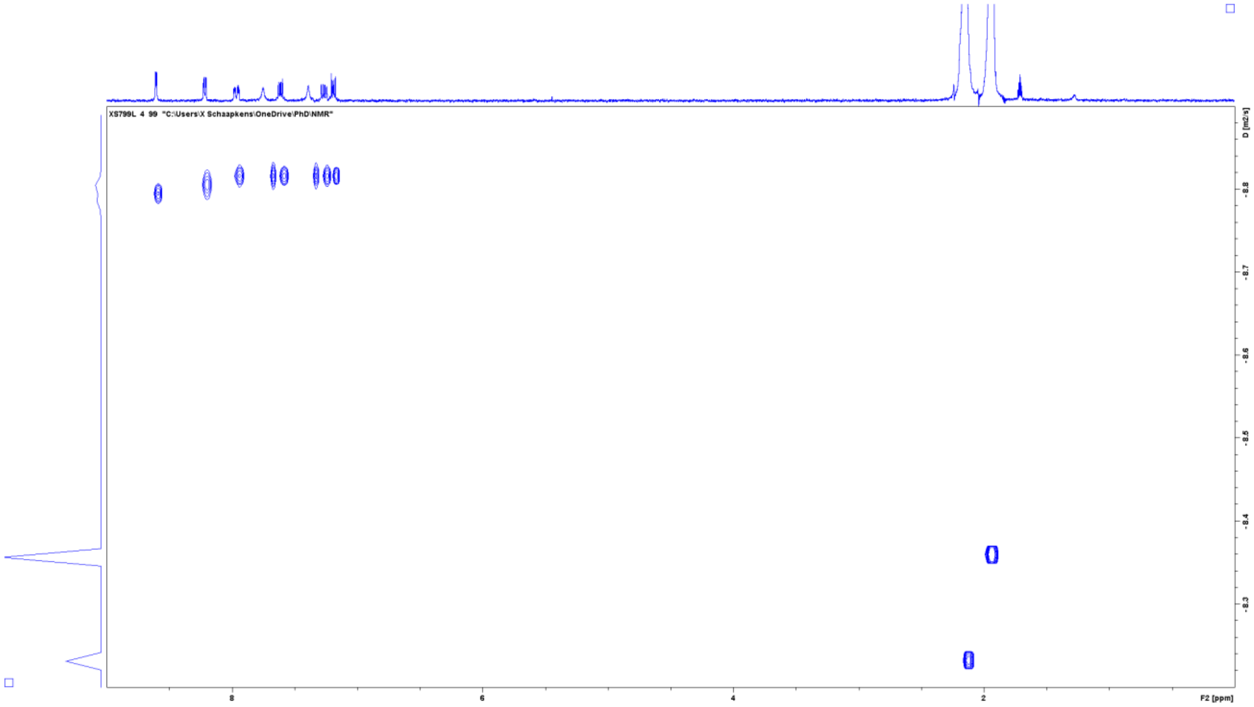

Figure S2-2. ^{1}H 2D-DOSY spectrum of 1,1'-(1,2-phenylene)bis(3-(pyridin-3-yl)urea) in CD_3CN @ 3.33 mM.

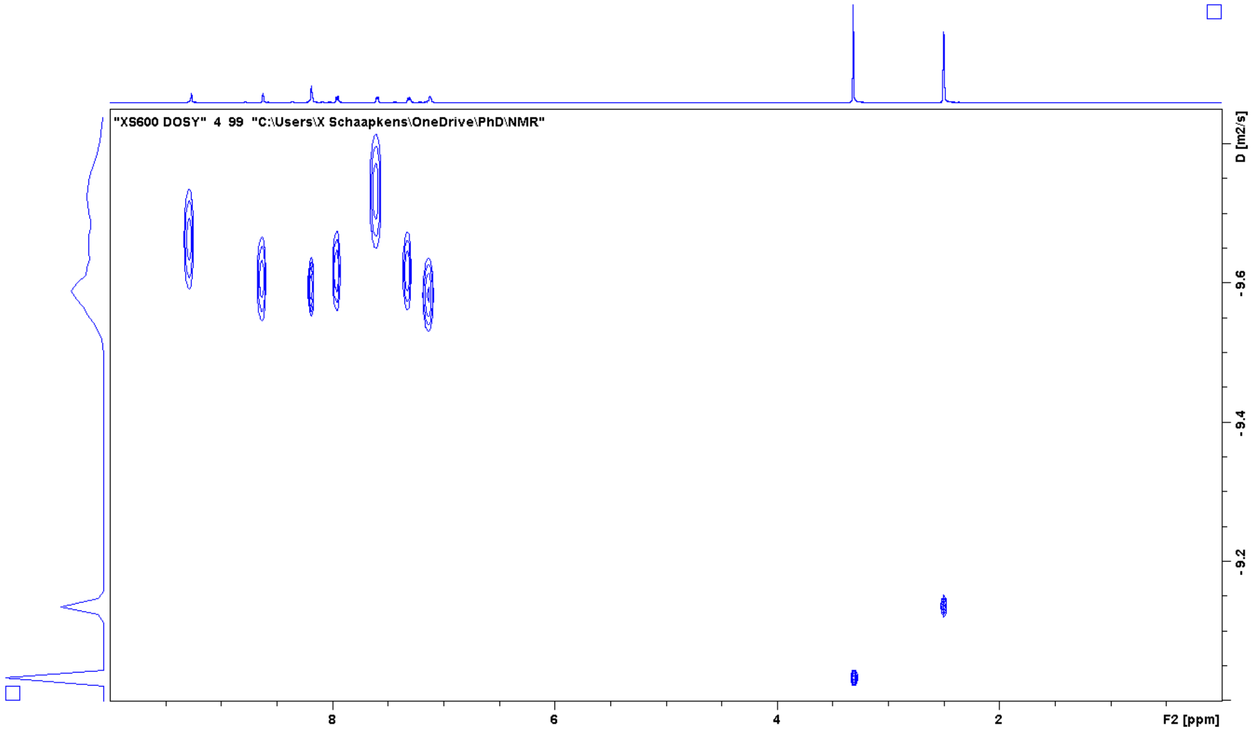

Figure S2-3. ^{1}H 2D-DOSY spectrum of 1,1'-(1,2-phenylene)bis(3-(pyridin-3-yl)urea) in DMSO-d_6 @ 3.33 mM

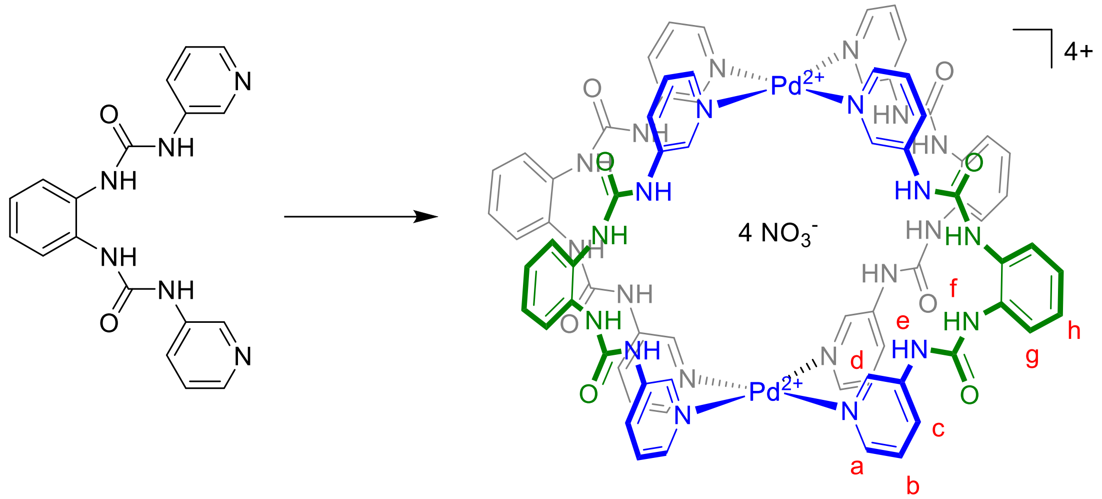

[4][NO$_3$]$_4$ was synthesized according to literature.[1] For DOSY NMR, the compound was isolated as a mixture of **4** containing two additional equivalents of the dipyridyl ligand (**L**). ^{1}H NMR (500 MHz, DMSO-d$_6$): δ 9.58 (s, 8H, H$_d$ **4**), 9.27 (s, 4H, H$_d$ **L**), 8.81 (s, 8H, H$_e$ **4**), 8.63 (s, 4H H$_e$ **L**), 8.29 – 8.21 (m, 16H, H$_a$ **4**, H$_a$ and H$_f$ **L**), 8.19 (s, 8H, H$_f$ **4**), 8.15 – 8.08 (m, 8H, H$_c$ **4**), 7.96 (d, J = 8.7 Hz, 4H, H$_c$ **4**), 7.63 – 7.47 (m, 20H, H$_b$, H$_g$ **4** and H$_b$ **L**), 7.31 (s, 4H, H$_g$ **L**), 7.20 – 7.07 (m, 12H, H$_h$ **4** and H$_h$ **L**). ^{1}H 2D-DOSY (500 MHz, DMSO-d$_6$): log (D) = -9.90 m2/s, with d20 = 0.12 sec and p30 = 500 µsec.

CSI HRMS: m/z calculated for $[(C_{18}H_{16}N_6O_2)_4Pd_2]^{4+}$ $[M]^{4+}$ 401.0847, found 401.0867 (Δ 4.99); calculated for $[(C_{18}H_{16}N_6O_2)_4Pd_2NO_3]^{3+}$ $[M+NO_3]^{3+}$ 555.4423, found 555.4438 (Δ 2.70); calculated for $[(C_{18}H_{16}N_6O_2)_4Pd_2(NO_3)_2]^{2+}$ $[M+2\ NO_3]^{2+}$ 865.1577, found 865.1587 (Δ 1.16).

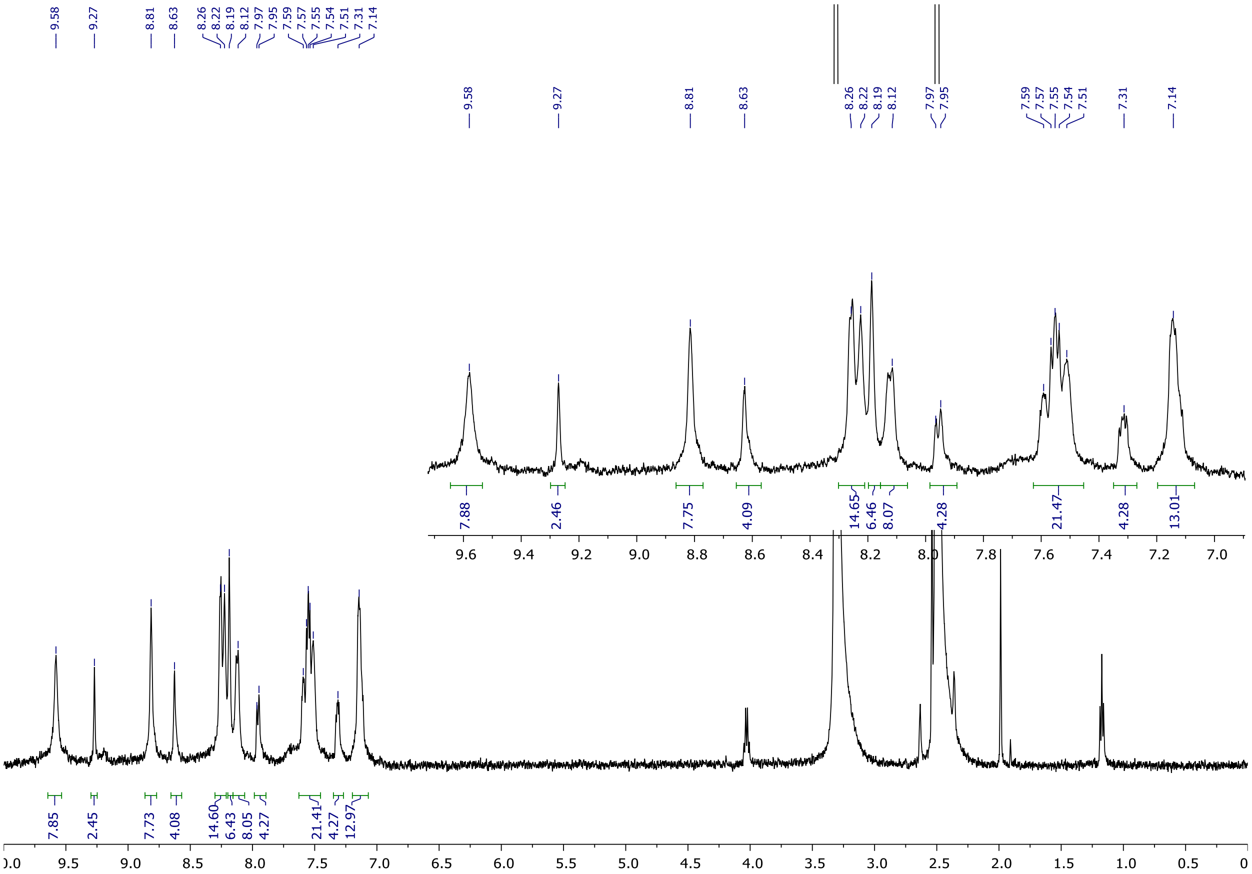

Figure S2-4. ^{1}H NMR spectrum of [**4**][NO_3^-]$_4$ with **L** in a 1:2 ratio in DMSO-d$_6$.

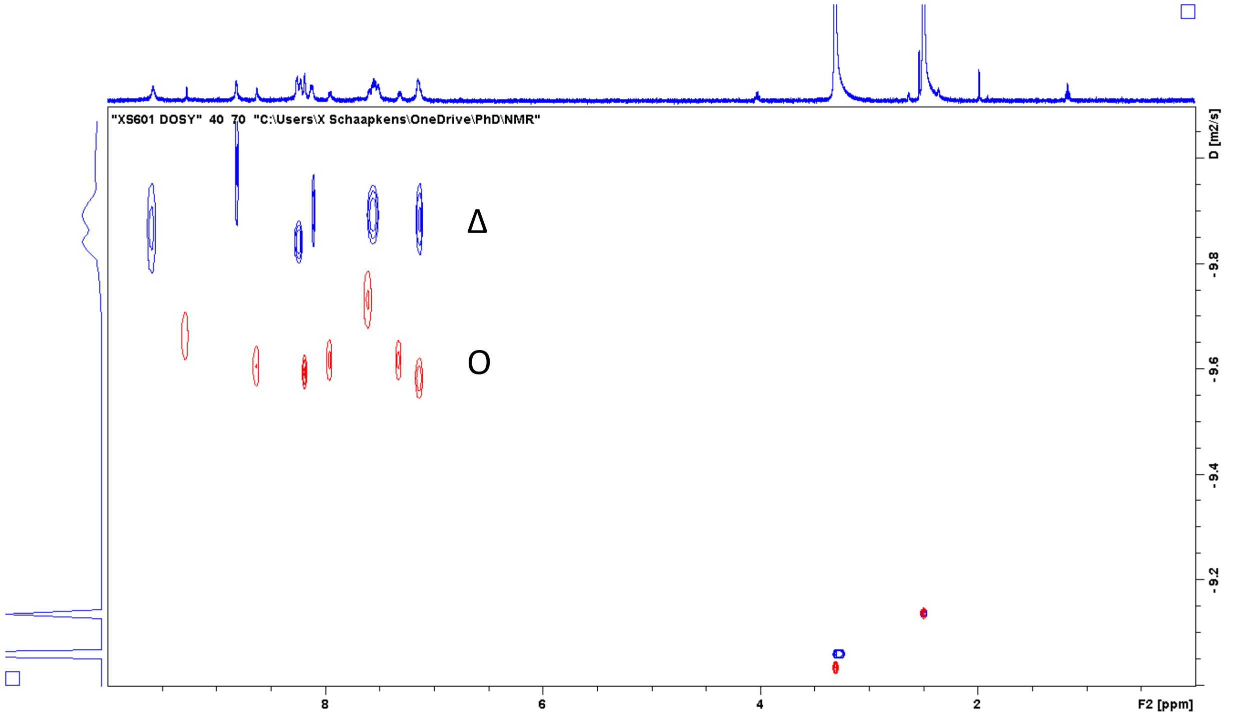

Figure S2-5. ^{1}H 2D-DOSY spectrum of [**4**][NO_3^-]$_4$ (Blue Δ) and two equivalents of the dipyridyl ligand (Red O) contained in the same DMSO-d$_6$ solution.

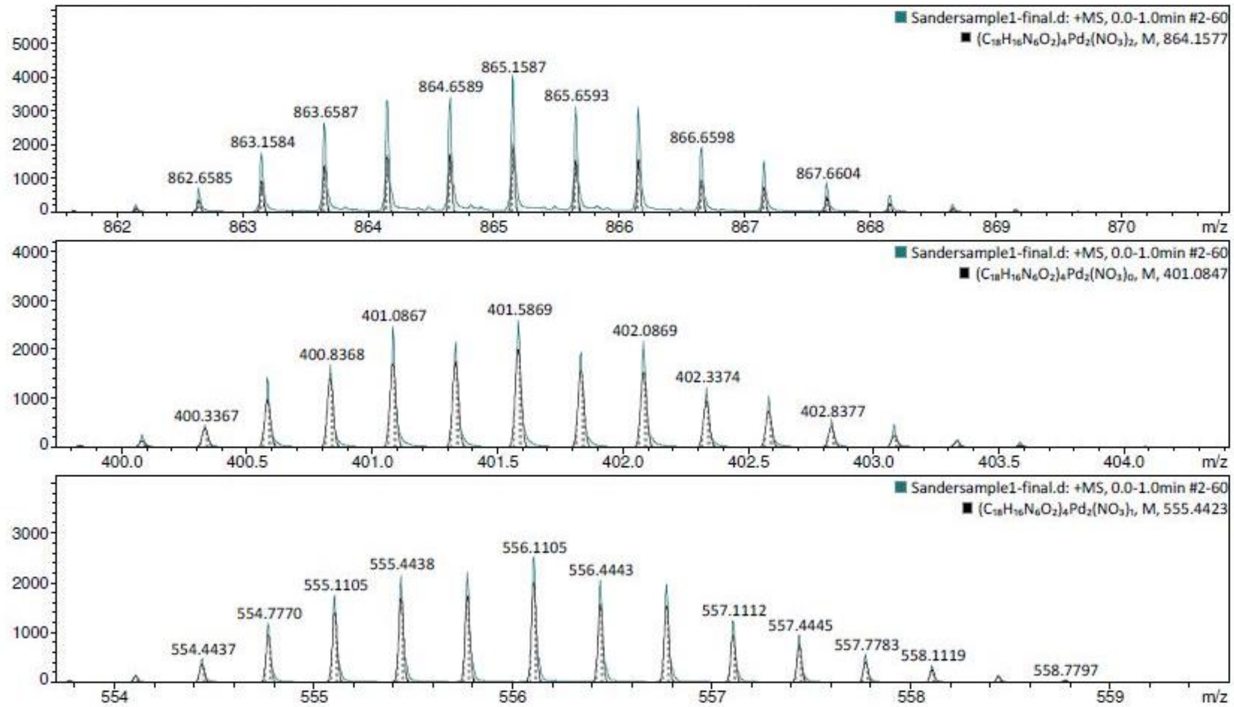

Figure S2-6. From top to bottom: CSI HRMS spectra of [**4**+2 NO_3]$^{2+}$, [**4**]$^{4+}$ and [**4**+NO_3]$^{3+}$ with calculated mass overlaying.

$$Pd(MeCN)_2Cl_2 + NaBAr^F \longrightarrow Pd(MeCN)_4(BAr^F)_2$$

Tetrakis(acetonitrile)palladium bis(tetrakis[3,5-bis(trifluoromethyl)phenyl]borate). To a solution of bis(acetonitrile)palladium dichloride (12.9 mg, 0.0497 mmol, 1 eq.) in DCM with 2% MeCN (1 mL) was added sodium tetrakis[3,5-bis(trifluoromethyl)phenyl]borate (89.8 mg, 0.101 mmol, 2.03 eq.). After stirring for 5 minutes, water with 2% MeCN (1 mL) was added and the resulting biphasic solution was vigorously stirred for 15 minutes. The water layer was pipetted off with a syringe and needle. The addition of water with 2% MeCN, subsequent vigorously stirring and pipetting off were repeated 5 times. The resulting organic layer was filtrated through a hydrophobic syringe filter, added to CD_3CN (1000 µL) and under a gentle flow of nitrogen the DCM was evaporated at 50 °C. (If necessary the solution was afterwards diluted to 1000 µL of CD_3CN to get the correct 49.7 mM concentration). The palladium BAr^F complex was used without further purification/isolation.

Note: The palladium BAr^F complex was stored as a solution in a *coordinating solven*t, because of its instability as a solid and in non-coordinating solvents.

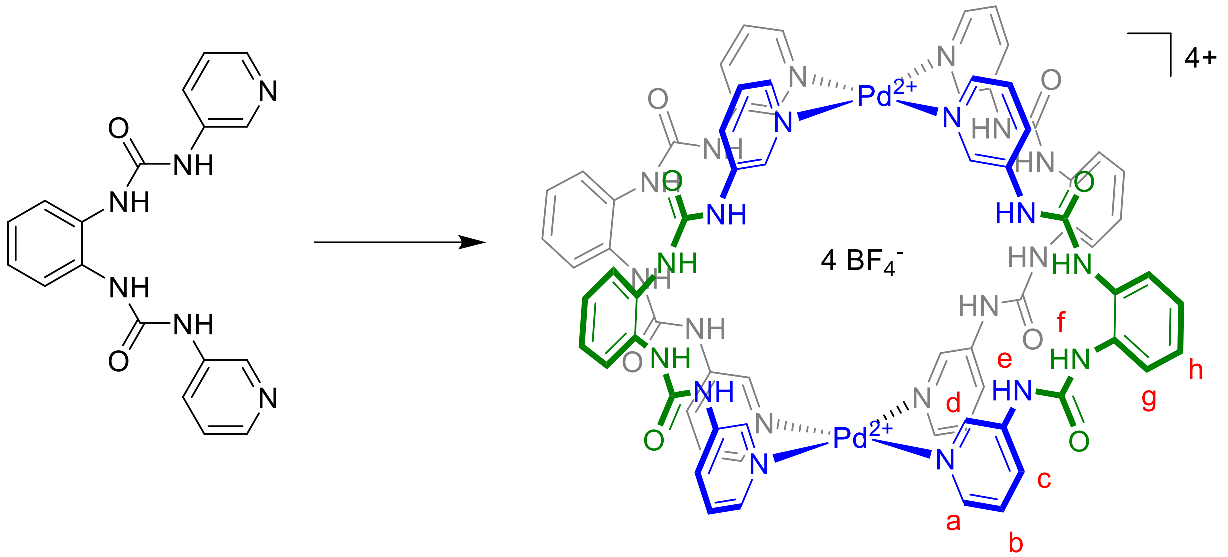

[4][BF$_4$]$_4$ CD$_3$CN/H$_2$O (97:3). To a solution of tetrakis(acetonitrile)palladium tetrafluoroborate in CD$_3$CN (10 µL, 1 µmol, 1 eq.) with CD$_3$CN (572 µL) and H$_2$O (18 µL) was added dipyridyl ligand (**L**, 0.7 mg, 2.01 µmol, 2 eq.) to afford a 0.83 mM solution of **4** in CD$_3$CN with 3% H$_2$O of a total volume of 600 µL.

^{1}H NMR (500 MHz, CD$_3$CN): δ 9.00 (s, 8H, H$_d$), 8.50 (s, 8H, H$_e$), 8.37 (s, 8H, H$_a$), 8.16 (s, 8H, H$_c$), 7.68 (s, 8H, H$_b$), 7.48 (s, 8H, H$_f$), 7.44 (dd, J = 8.5, 5.6 Hz, 8H, H$_g$), 7.08 (dd, J = 6.2, 3.5 Hz, 8H, H$_h$). ^{11}B NMR (96 MHz, CD$_3$CN): δ -1.01 (s, 4B). ^{19}F NMR (282 MHz, CD$_3$CN): δ -150.52 (d, J = 15.3 Hz, 16F). ^{1}H 2D-DOSY (500 MHz, CD$_3$CN): log (D) = -8.87 m^2/s, with d20 = 0.12 sec and p30 = 500 µsec. {^{1}H-^{19}F}-HOESY (300, 282 MHz, CD$_3$CN): -150.52-9.00 (F-H$_d$), with t_m = 500 ms.

CSI HRMS: m/z calculated for [(C$_{18}$H$_{16}$N$_6$O$_2$)$_4$Pd$_2$]$^{4+}$ [M]$^{4+}$ 401.5855, found 401.5839 (Δ -3.98); calculated for [(C$_{18}$H$_{16}$N$_6$O$_2$)$_4$Pd$_2$BF$_4$]$^{3+}$ [M+BF$_4$]$^{3+}$ 564.4487, found 564.4456 (Δ -5.49); calculated for [(C$_{18}$H$_{16}$N$_6$O$_2$)$_4$Pd$_2$(BF$_4$)$_2$]$^{2+}$ [M+2 BF$_4$]$^{2+}$ 890.1752, found 890.1710 (Δ -4.72); calculated for [(C$_{18}$H$_{16}$N$_6$O$_2$)$_4$Pd$_2$(BF$_4$)$_3$]$^{1+}$ [M+3 BF$_4$]$^{1+}$ 1866.3549, found 1866.3489 (Δ -3.21). **NB:** These reported calculated values were obtained from Bruker DataAnalysis 4.4 software, wherein the positive ionization is taken into account (0.0005486 Da per electron). The isotope distributions shown in the figures were simulated with mMass software, which *does not* account for missing electrons. This leads to apparent mass differences between the reported values above and those shown in the figures as large as 0.0021944 Da (4 x 0.0005486) in a 4+ species.

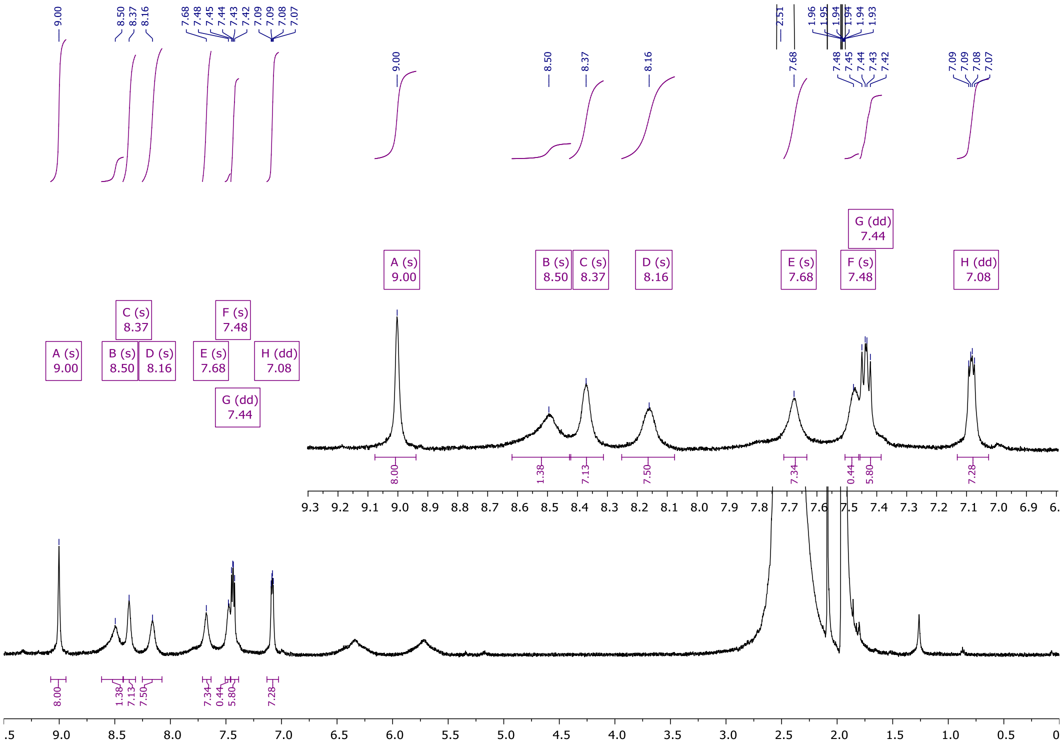

Figure S2-7. ^{1}H NMR spectrum of [**4**][BF_4^-]$_4$ in CD_3CN.

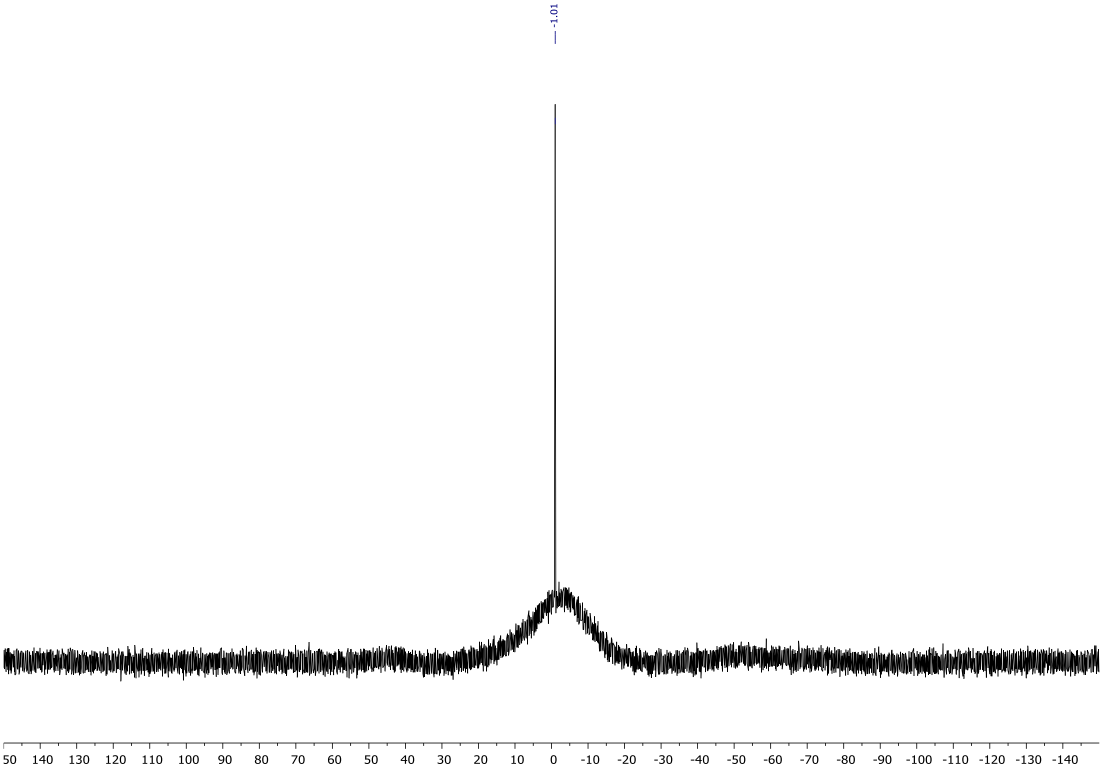

Figure S2-8. ^{11}B NMR spectrum of [**4**][BF_4^-]$_4$ in CD_3CN.

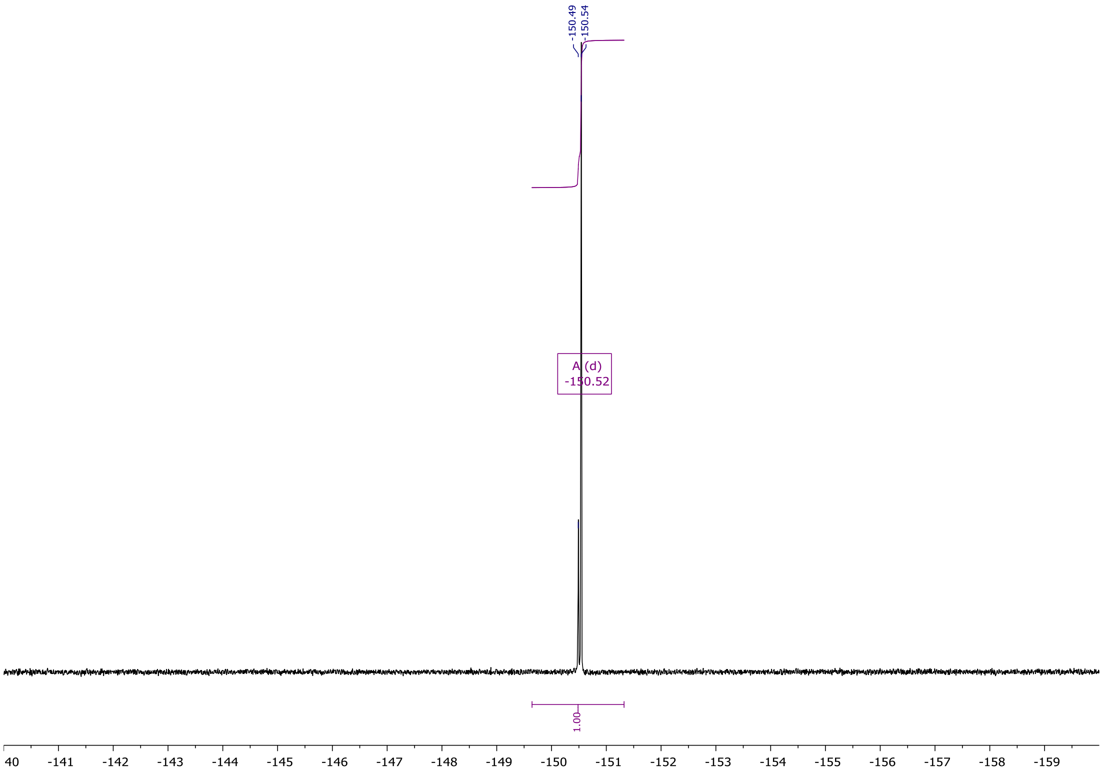

Figure S2-9. ^{19}F NMR spectrum of [**4**][BF_4^-]$_4$ in CD_3CN.

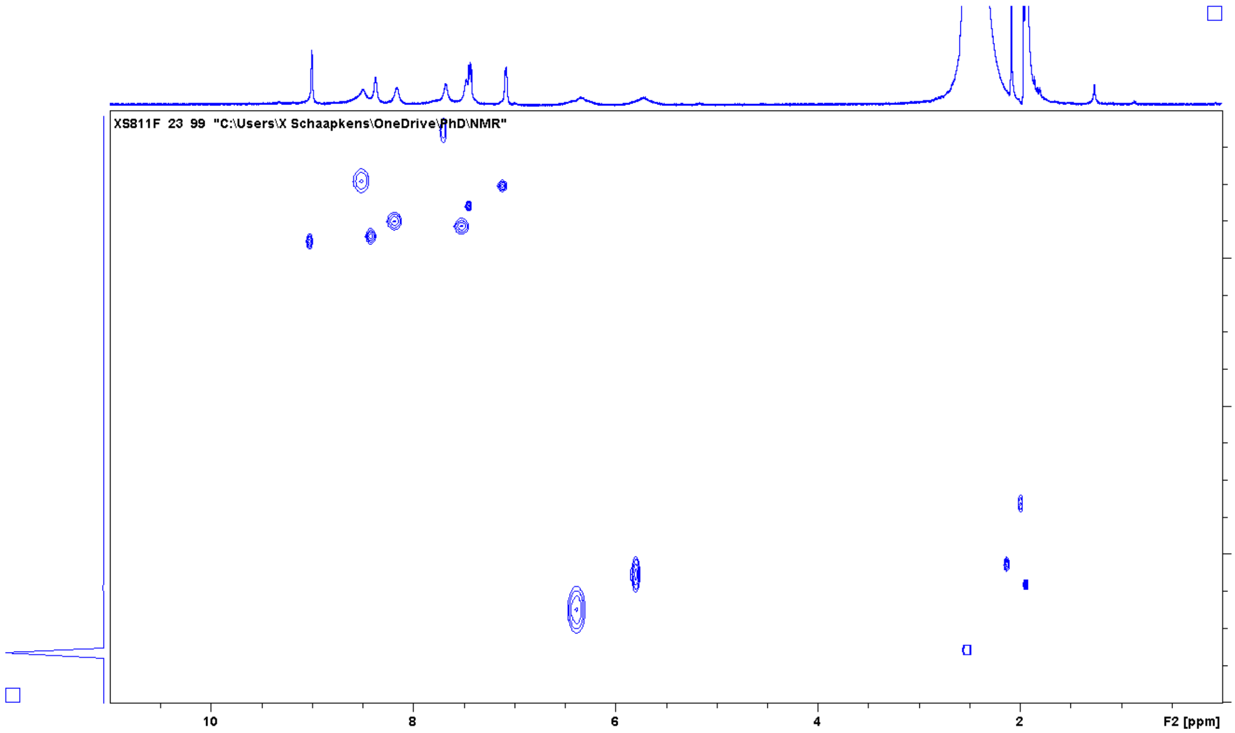

Figure S2-10. ^{1}H 2D-DOSY spectrum of [**4**][BF_4^-]$_4$ in CD_3CN.

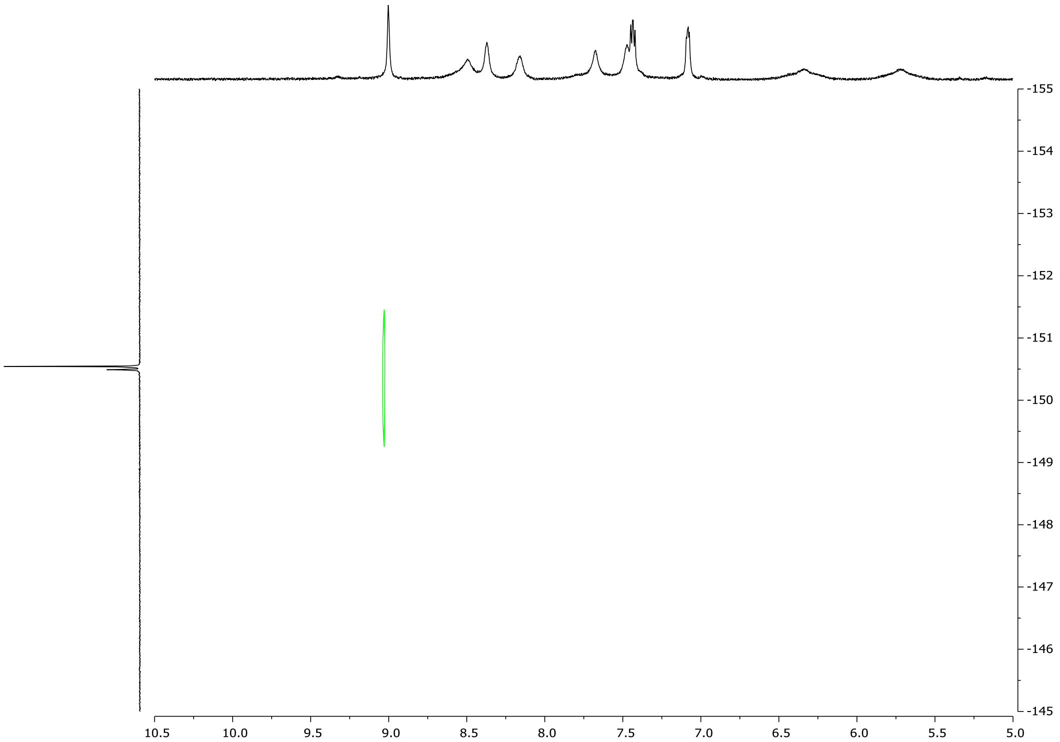

Figure S2-11. {^{1}H-^{19}F}-HOESY spectrum of [**4**][BF_4^-]$_4$ in CD_3CN; the big cross-peak is between the ^{19}F-signal f BF_4^- and the inwards pointing CH of the pyridyl ligands (**d**, see Figure 2).

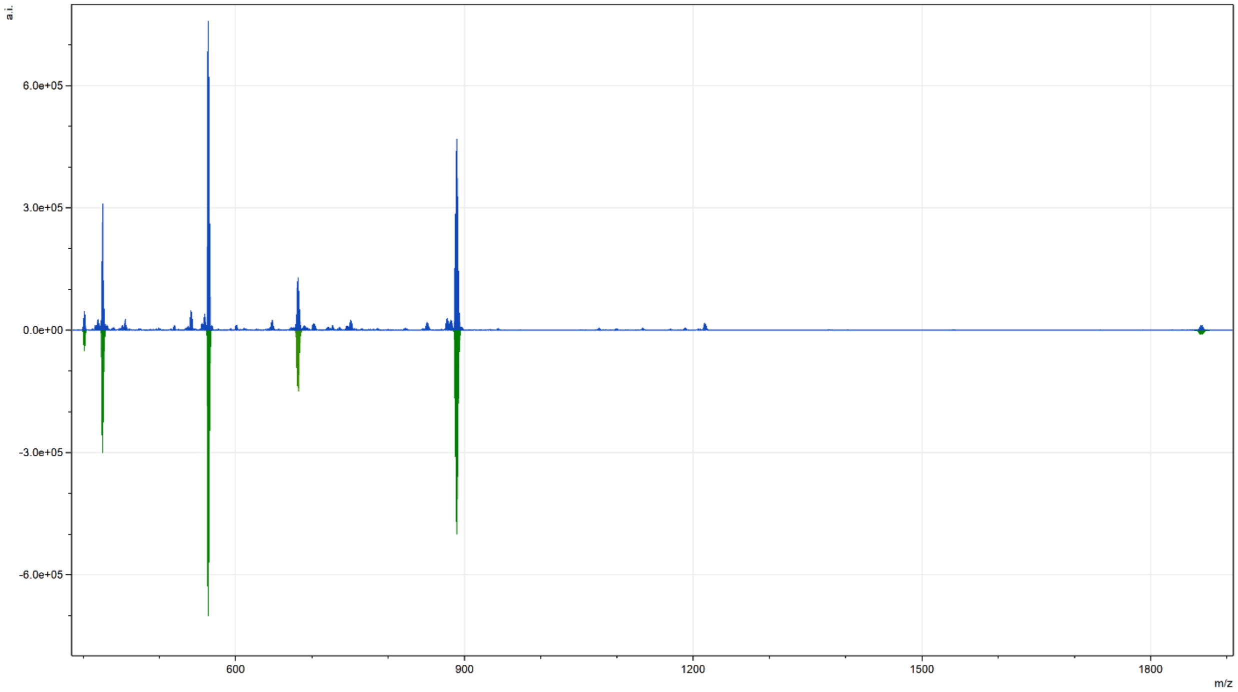

Figure S2-12. CSI HRMS of a solution containing **4** (top) and calculated spectrum (bottom) full spectrum.

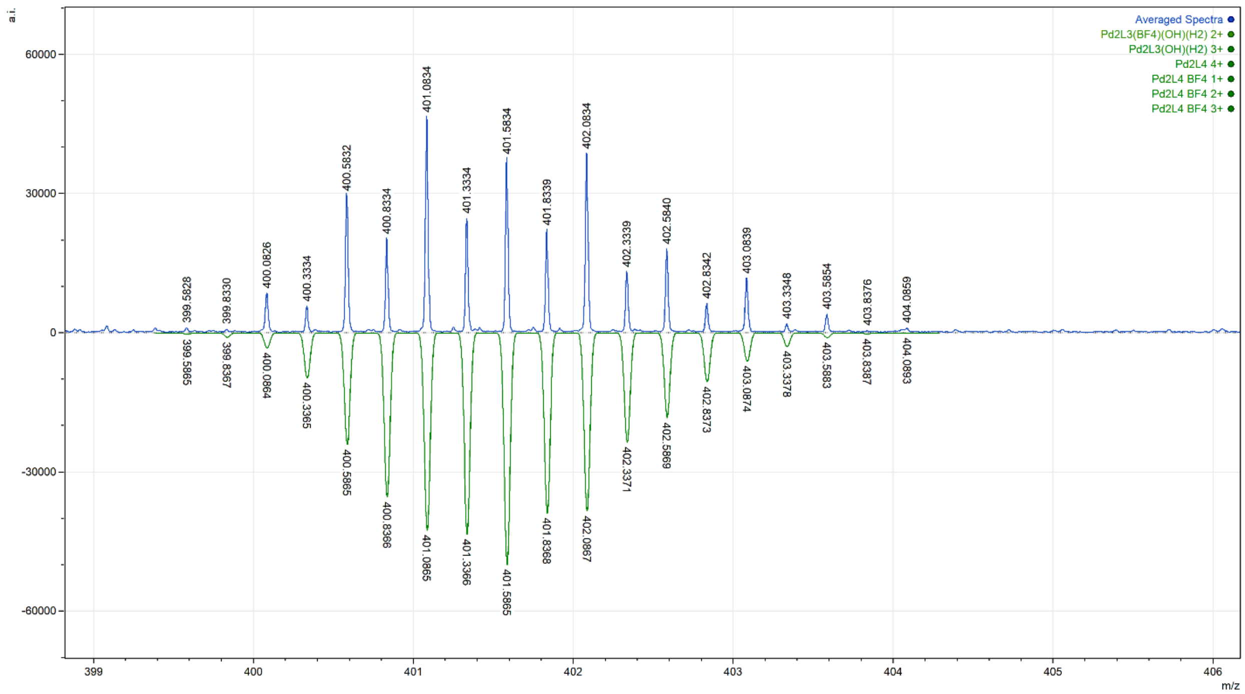

Figure S2-13. CSI HRMS of [**4**]$^{4+}$ (top) and calculated spectrum (bottom) around 401 m/z.

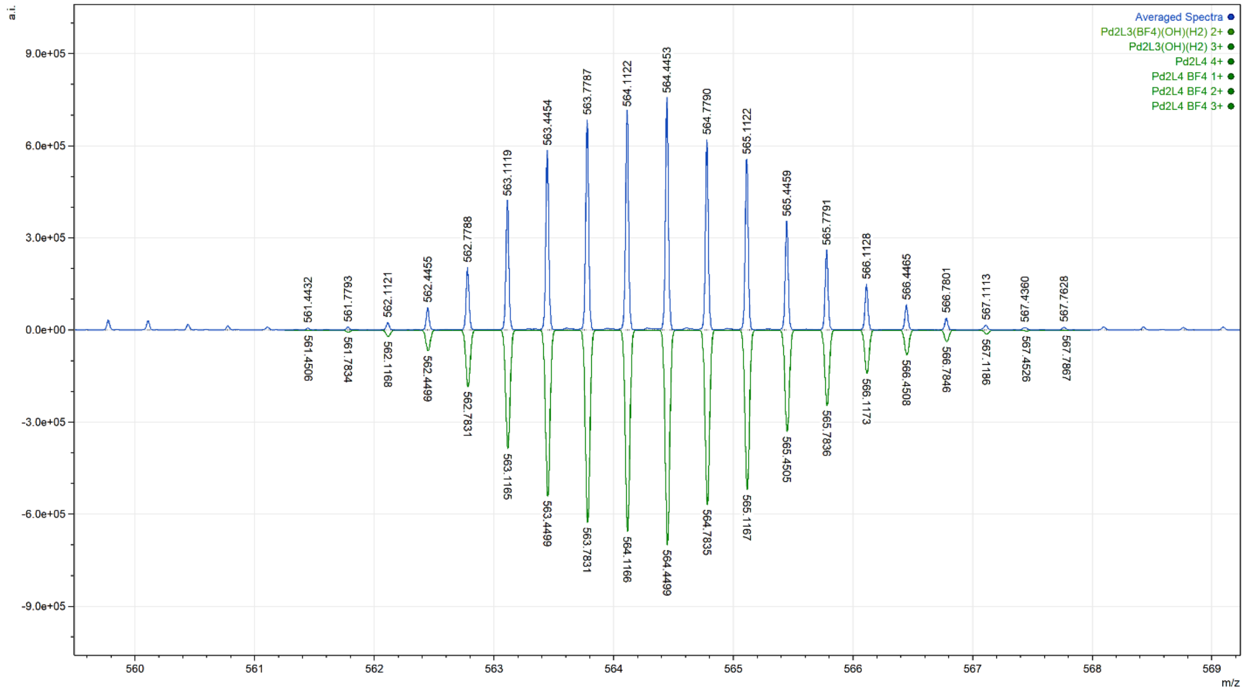

Figure S2-14. CSI HRMS of [**4**+BF_4]$^{3+}$ (top) and calculated spectrum (bottom) around 564 m/z.

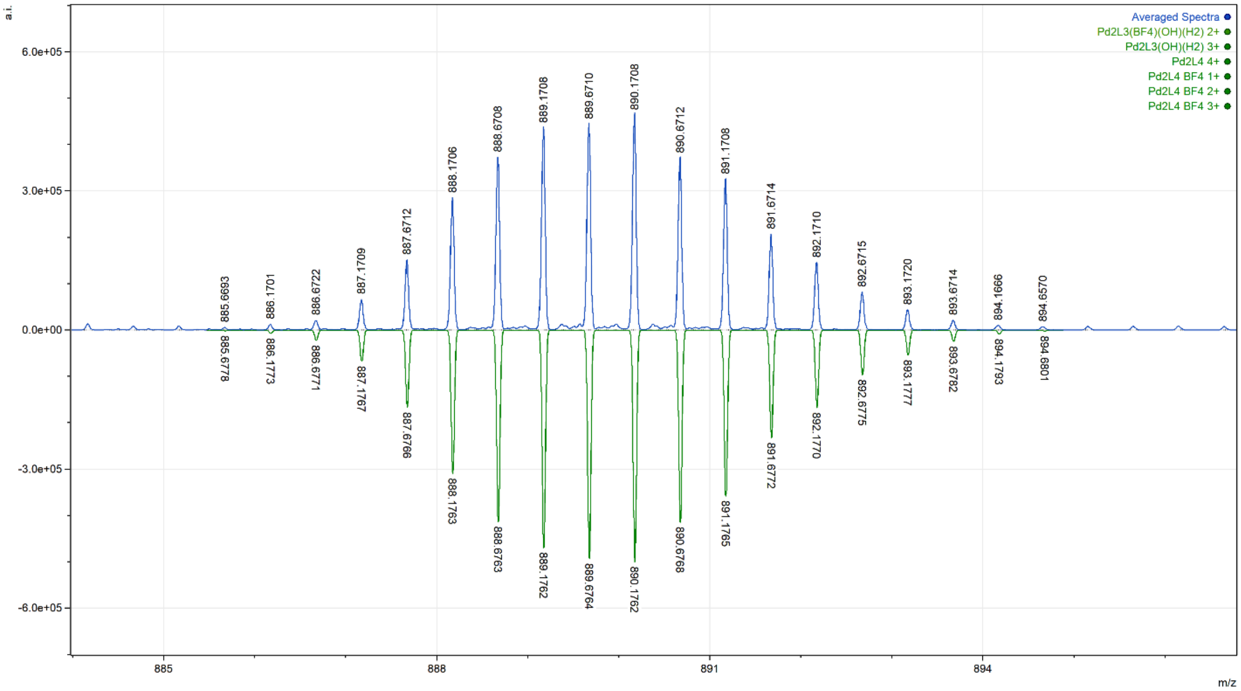

Figure S2-15. CSI HRMS of [**4**+2 BF_4]$^{2+}$ (top) and calculated spectrum (bottom) around 890 m/z.

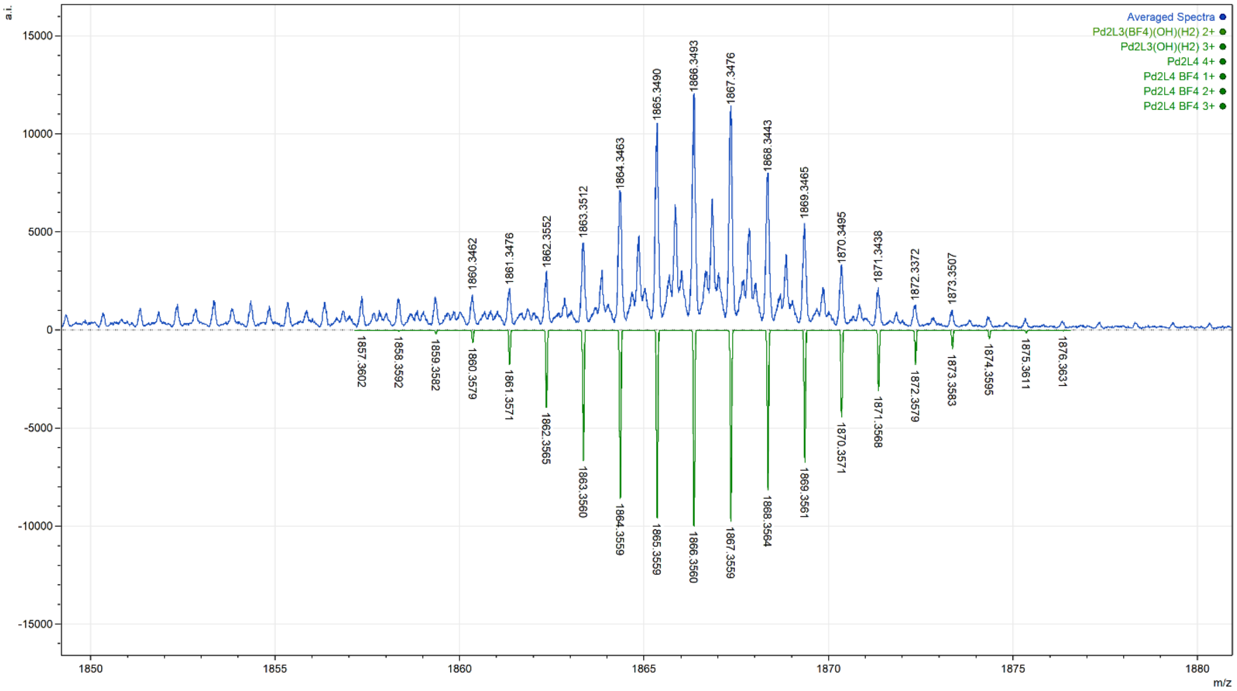

Figure S2-16. CSI HRMS of [**4**+3 BF_4]$^{1+}$ (top) and calculated spectrum (bottom) around 1866 m/z.

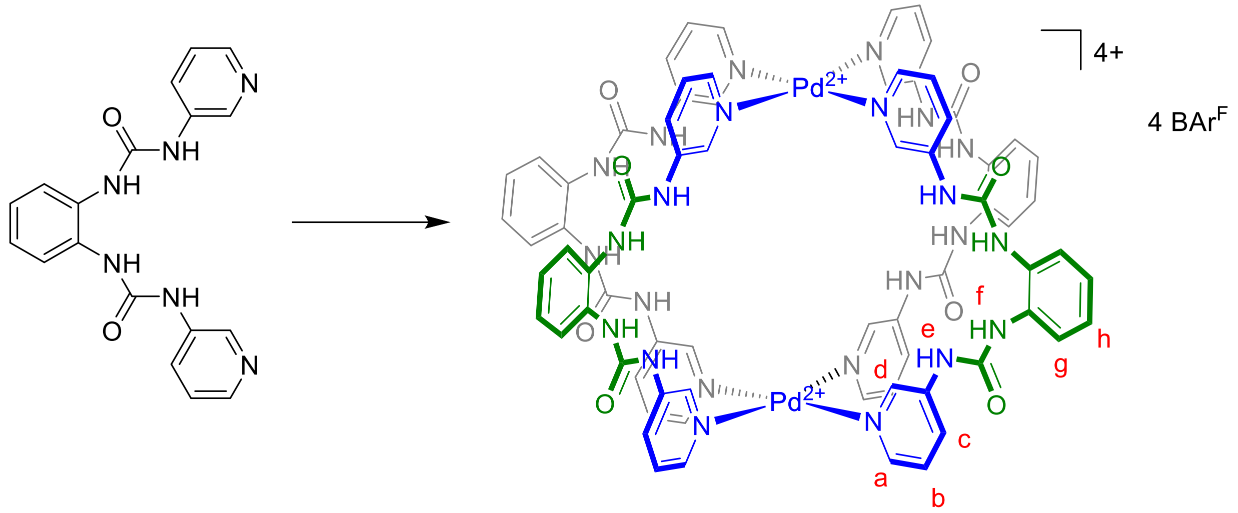

$[Pd_2L2_4][BAr^F]_4$ ($4[BAr^F]_4$) in CD_3CN/H_2O (9.7:0.3). To a solution of the 49.7 mM palladium BAr^F solution (24 µL, 1.2 µmol, 1 eq.) with CD_3CN (558 µL) and H_2O (18 µL) was added dipyridyl ligand (**L**, 0.7 mg, 2.01 µmol, 1.68 eq.) to afford a 0.83 mM cage solution in CD_3CN with 3% H_2O of a total volume of 600 µL.

^1H NMR (500 MHz, CD_3CN): δ 9.15 (s, 8H, H_e), 9.13 (s, 8H, H_d), 8.53 (d, J = 5.2 Hz, 8H, H_a), 8.44 (d, J = 8.5 Hz, 8H, H_c), 7.79 (s, 8H, H_f), 7.67 (m, 56H, H_b, H_{BArF}), 7.46 (dd, J = 8.6, 5.5 Hz, 8H, H_g), 7.08 (dd, J = 6.2, 3.5 Hz, 8H, H_h). ^{11}B NMR (96 MHz, CD_3CN): δ -6.70 (s, 4B). ^{19}F NMR (282 MHz, CD_3CN): δ -63.24 (s, 24F). ^1H 2D-DOSY (500 MHz, CD_3CN): log (*D*) = -9.04 m^2/s, with d20 = 0.12 sec and p30 = 500 µsec.

CSI HRMS: m/z calculated for $[(C_{18}H_{16}N_6O_2)_4Pd_2]^{4+}$ $[M]^{4+}$ 401.5855, found 401.585 (Δ 1.24); calculated for $[(C_{18}H_{16}N_6O_2)_4Pd_2(C_{32}H_{12}BF_{24})]^{3+}$ $[M+BAr^F]^{3+}$ 823.1364, found 823.1339 (Δ 3.03); calculated for $[(C_{18}H_{16}N_6O_2)_4Pd_2(C_{32}H_{12}BF_{24})_2]^{2+}$ $[M+2\ BAr^F]^{2+}$ 1666.2383, found 1666.2324 (Δ 3.54); calculated for $[(C_{18}H_{16}N_6O_2)_4Pd_2(C_{32}H_{12}BF_{24})Cl_2]^{1+}$ $[M+BAr^F+2\ Cl]^{1+}$ 2539.3468, found 2539.3368 (Δ 3.94). **NB:** These reported calculated values were obtained from Bruker DataAnalysis 4.4 software, wherein the positive ionization is taken into account (0.0005486 Da per electron). The isotope distributions shown in the figures were simulated with mMass software, which *does not* account for missing electrons. This leads to apparent mass differences between the reported values above and those shown in the figures as large as 0.0021944 Da (4 x 0.0005486) in a 4+ species.

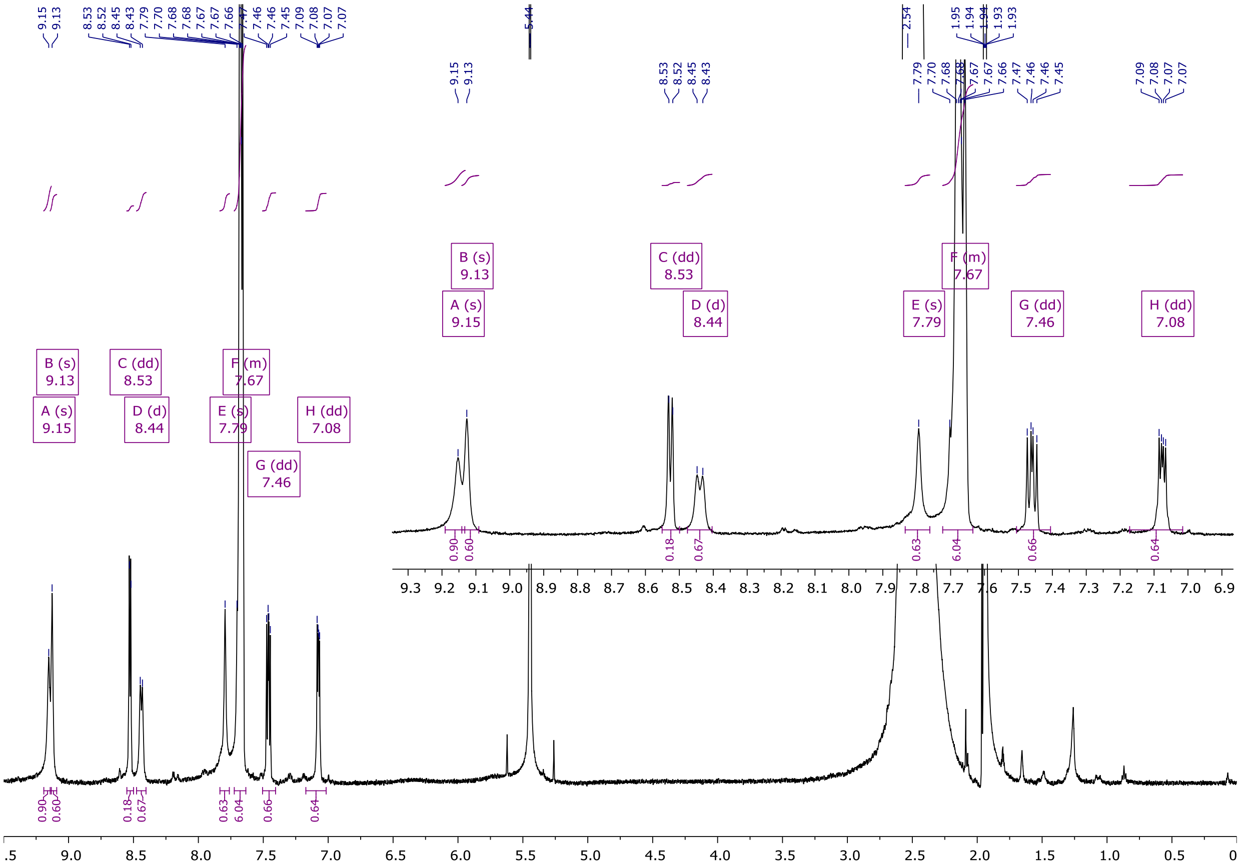

Figure S2-17. ^{1}H NMR spectrum of [**4**][BAr^F]$_4$ in CD_3CN.

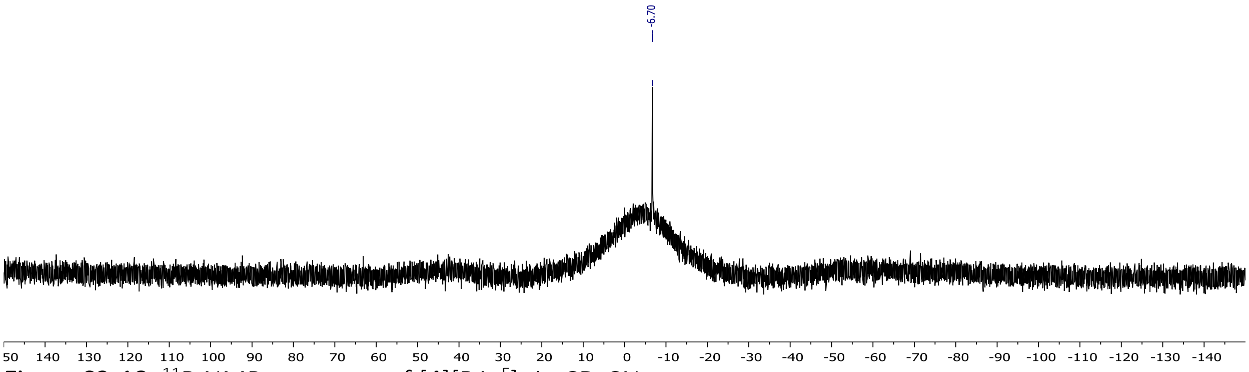

Figure S2-18. ^{11}B NMR spectrum of [**4**][BAr^F]$_4$ in CD_3CN.

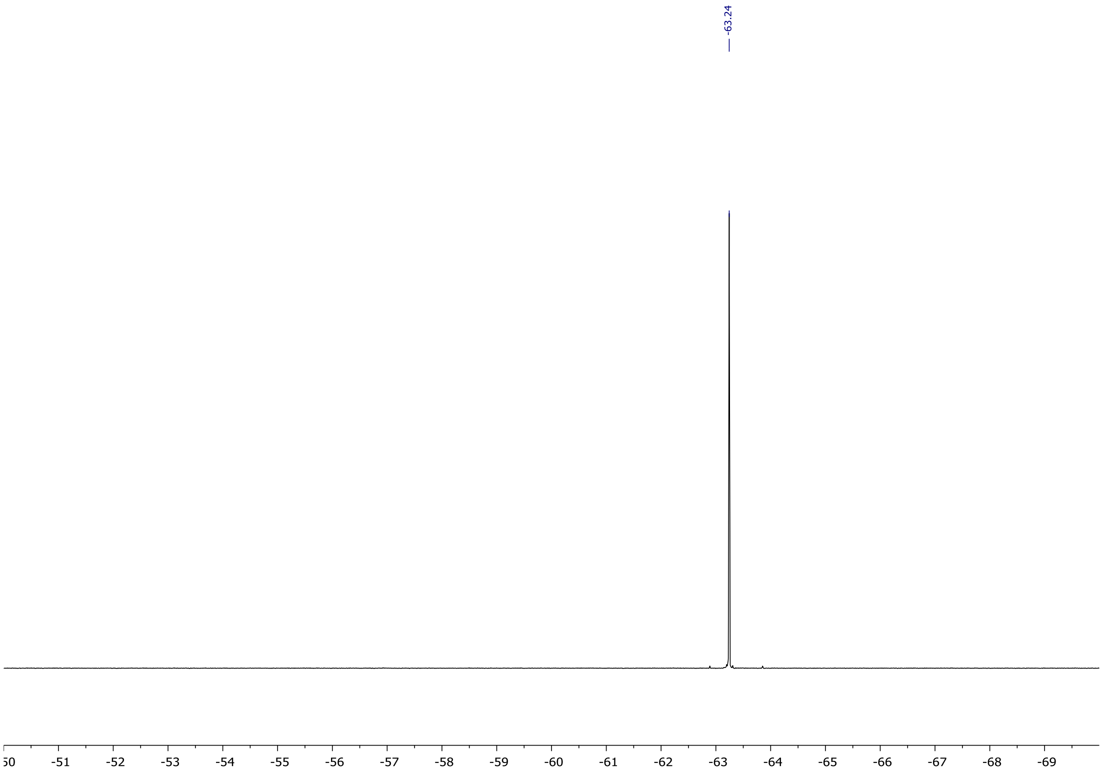

Figure S2-19. ^{19}F NMR spectrum of [**4**][BAr^F]$_4$ in CD_3CN.

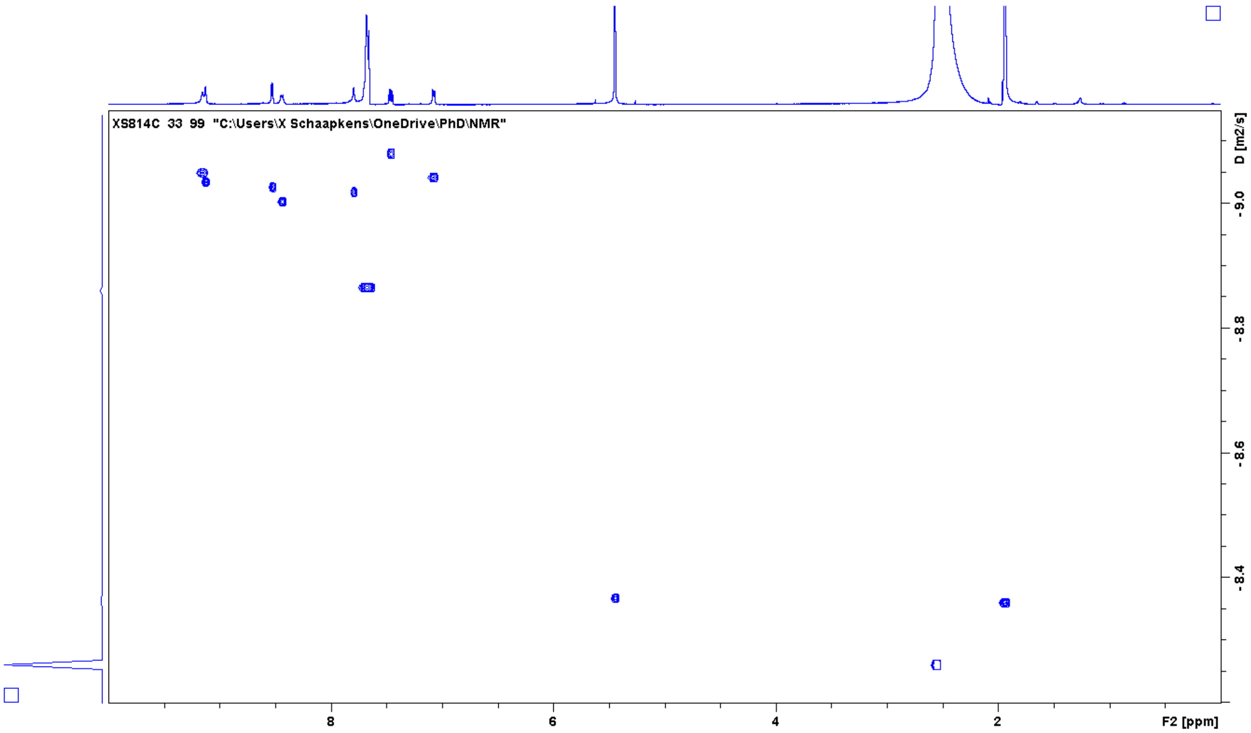

Figure S2-20. ^{1}H 2D-DOSY spectrum of [**4**][BAr^F]$_4$ in CD_3CN.

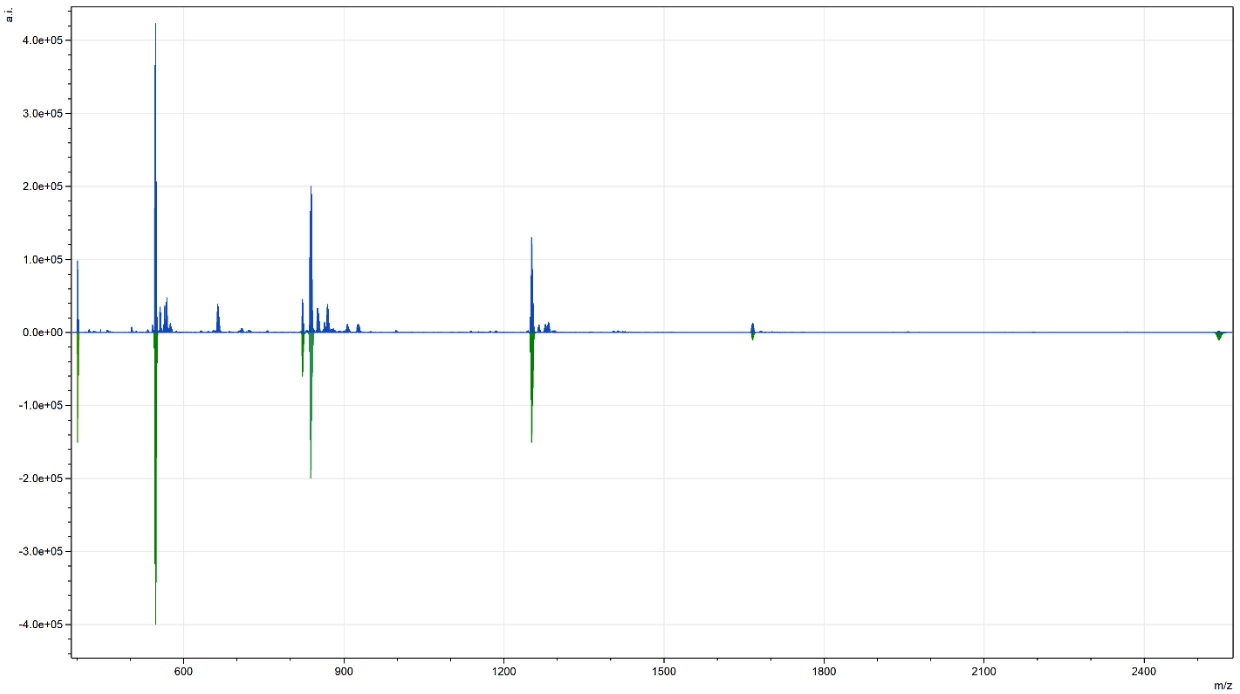

Figure S2-21. CSI HRMS of full spectrum of a solution containing **4** (top) and calculated spectrum (bottom).

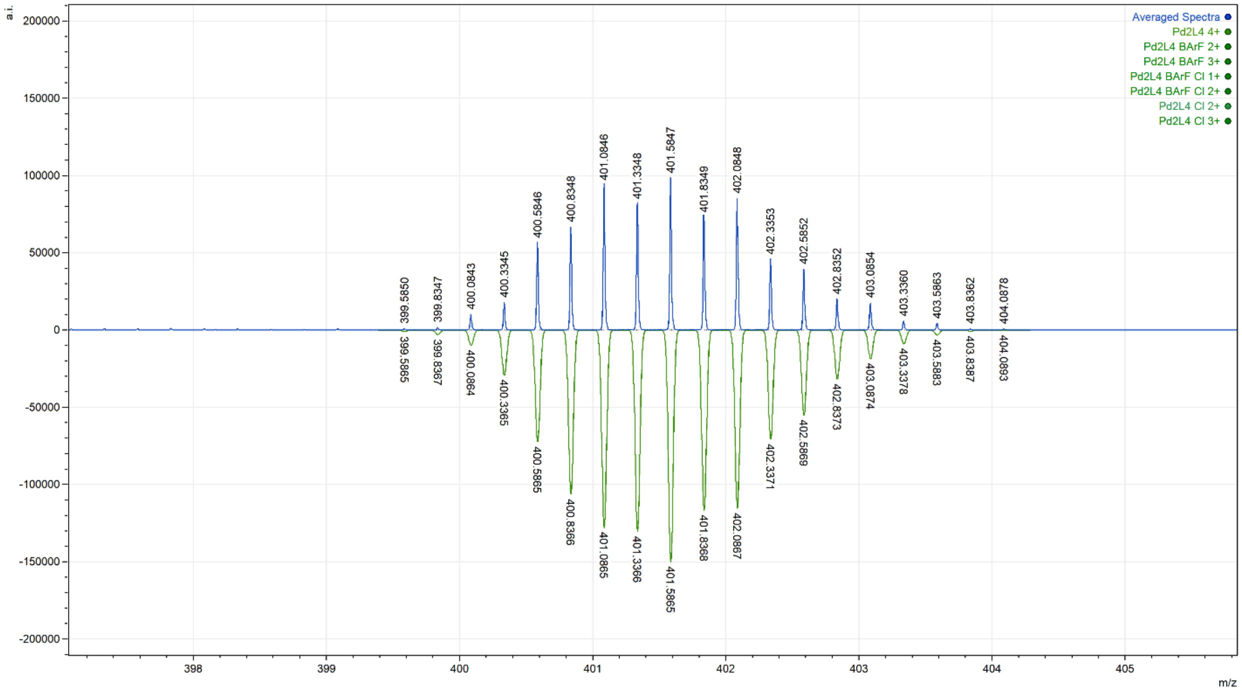

Figure S2-22. CSI HRMS of [**4**]$^{4+}$ (top) and calculated spectrum (bottom) around 401 m/z.

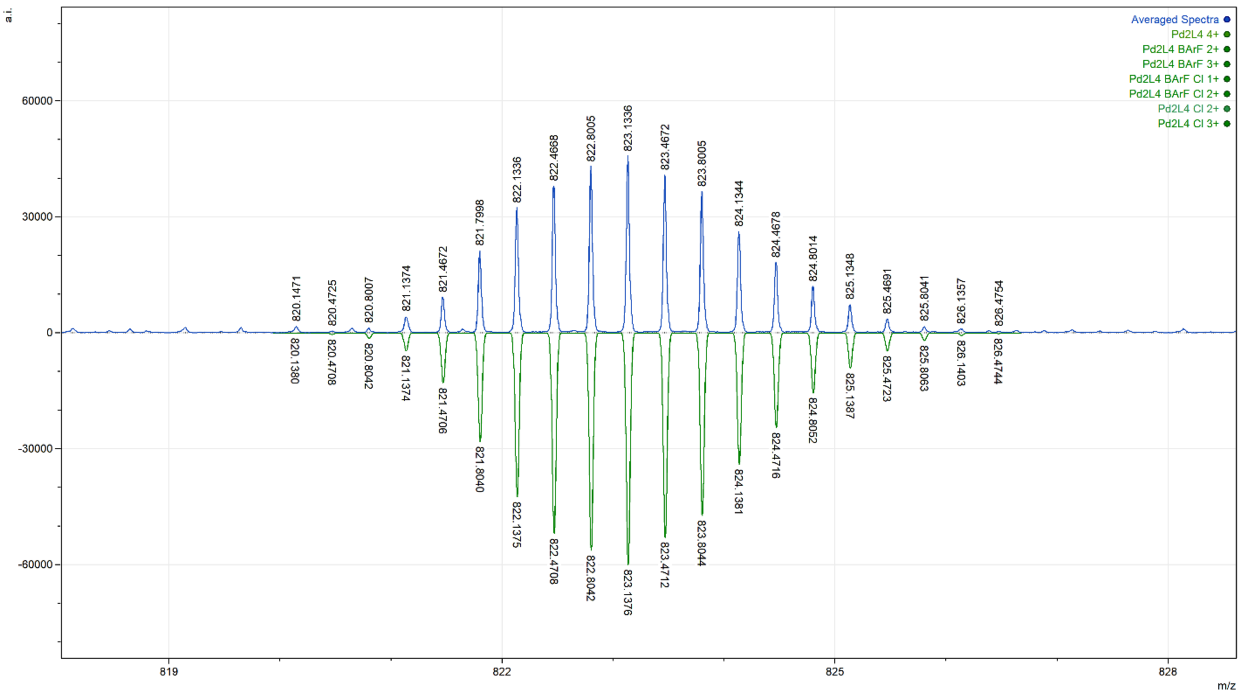

Figure S2-23. CSI HRMS of [**4**+BAr^F]$^{3+}$ (top) and calculated spectrum (bottom) around 823 m/z.

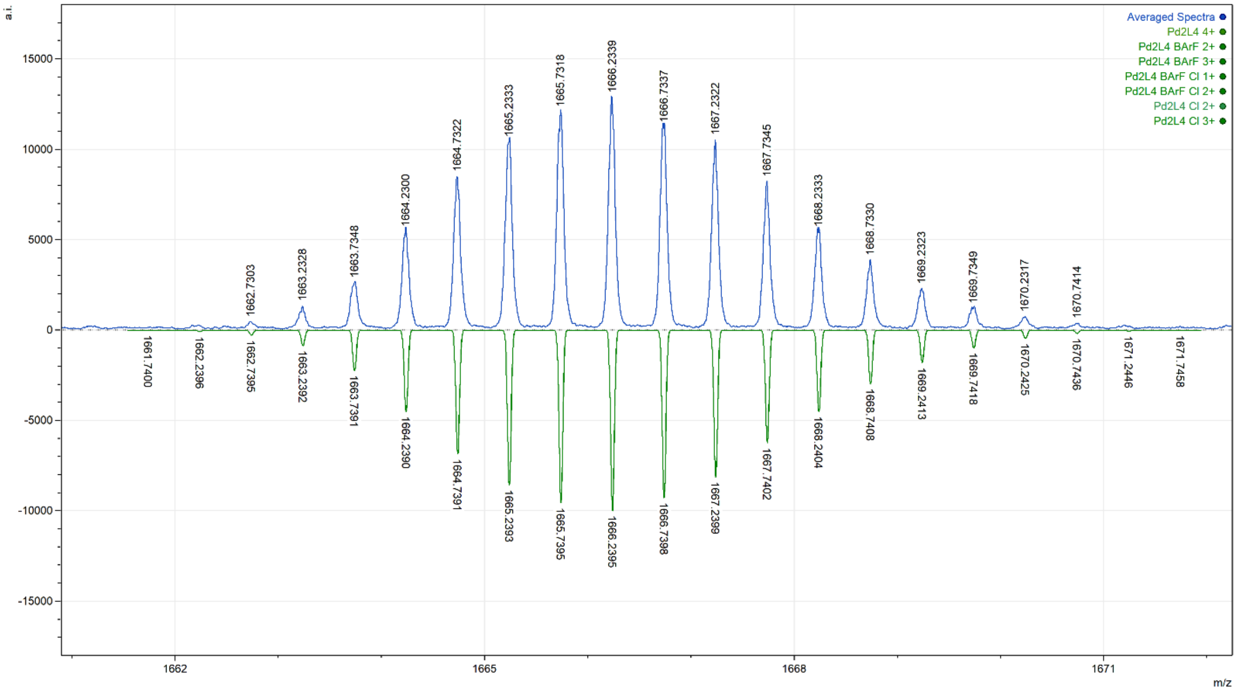

Figure S2-24. CSI HRMS of [**4**+2 BAr^F]$^{2+}$ (top) and calculated spectrum (bottom) around 1666 m/z.

Section S3. NMR studies of 4

Section S3a. Studies of [4][NO_3^-]$_4$

The [**4**][NO_3^-]$_4$ complex reported by Chand was prepared according to their procedure (see also section S2).[1] The resulting compound was readily soluble in DMSO-d_6, but found to be insoluble in water or acetonitrile. A titration of [**4**][NO_3^-]$_4$ with *n*-octyl-b-D-glucoside (**5**) in pure DMSO-d_6 was followed by NMR and is shown in Figure S3-1. The shifts that did occur could not be fitted to a 1:1 binding model . The lack of apparent binding can be rationalized based on the crystal structure of [**4**][NO_3^-]$_4$,[1] where nitrate anions are firmly bound to the interior of the coordination cage. Presumably, the bis-urea cage has a high affinity for nitrate in DMSO, thus preventing the binding of glucoside **5**.

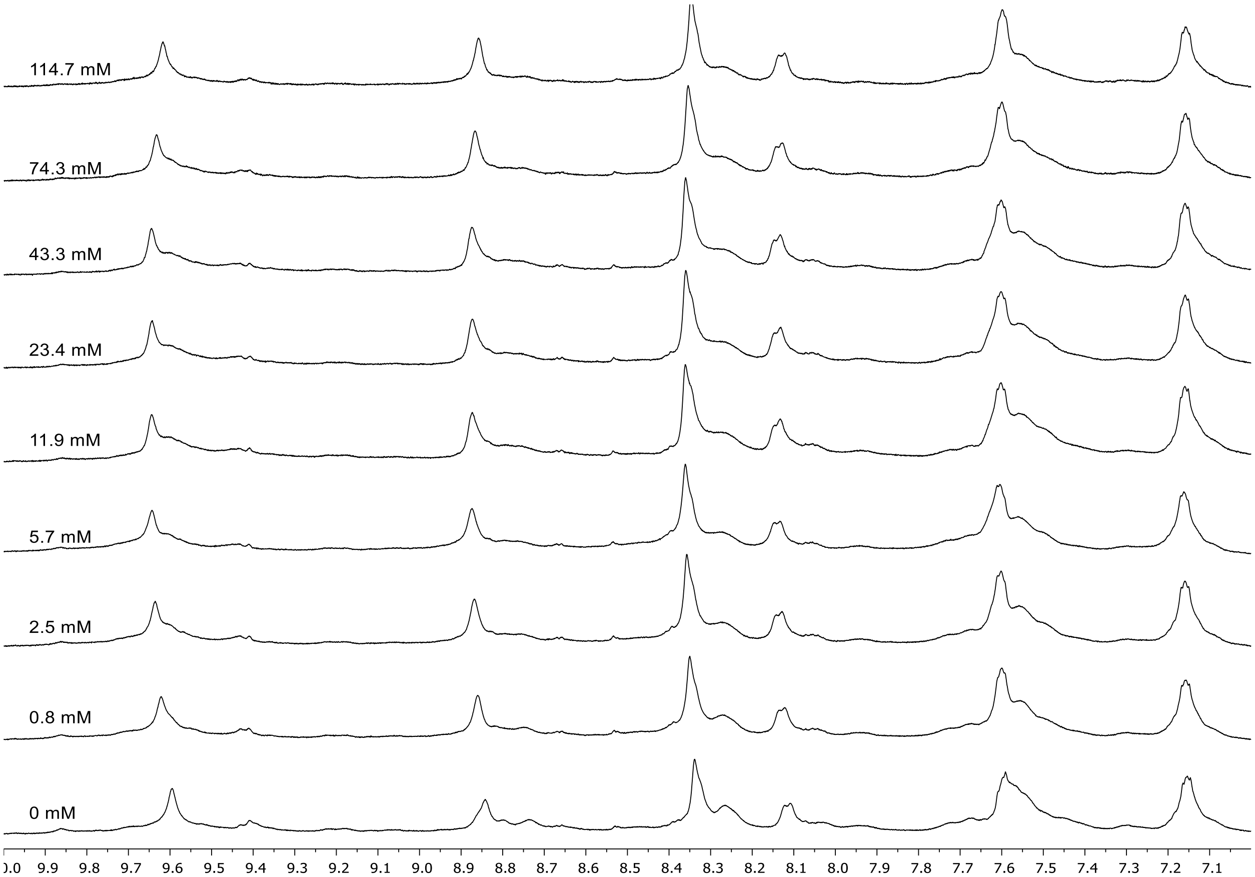

Figure S3-1. ^{1}H-NMR spectra of a binding study of [**4**][NO_3^-]$_4$ with *n*-octyl-β-D-glucoside (**5**, total concentration indicated) in DMSO-d_6.

Section S3b. Studies of [4][BF_4^-]$_4$ and [4][BAr^F]$_4$ in DMSO-d$_6$

In pure DMSO-d$_6$, synthesis of [**4**][(BF_4^-)$_4$] and [**4**][(BAr^F)$_4$] gave complex ^{1}H-NMR spectra as can be seen in Figure S3-2e and b respectively. These spectra could be somewhat resolved when measured at 80 °C as shown in Figure 3-2d and a. Moreover, addition of *n*-octyl-β-D-glucoside to these cages resulted in more resolved spectra. In the case of [**4**][(BF_4)$_4$] (Figure S3-2f) one apparent major species was observed, while the spectrum of [**4**][(BAr^F)$_4$] in the presence of **5** (Figure S3-2c) was more complex, yet better resolved that the spectrum of [**4**][(BAr^F)$_4$] alone (Figure 3-2b).

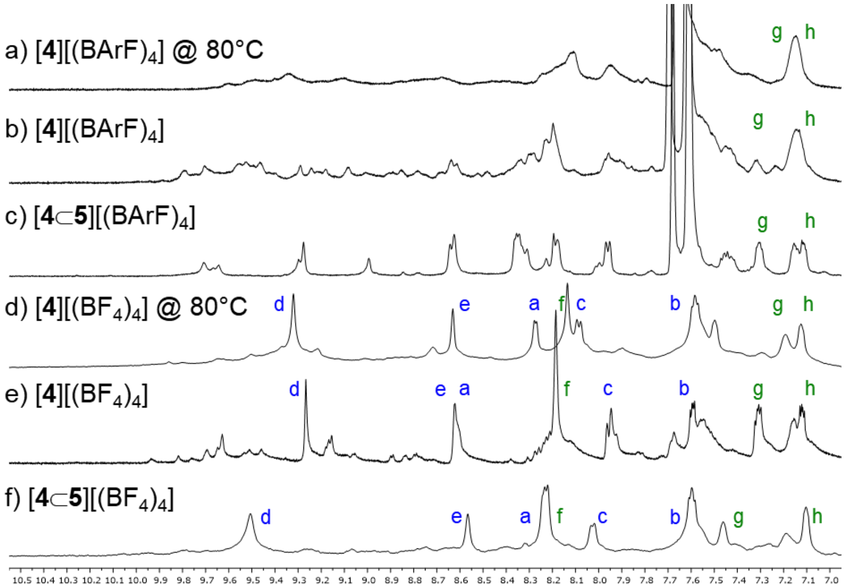

Figure S3-2. Comparison of [**4**][(BAr^F)$_4$] at room temperature (b) with heating at 80 °C (a) or addition of glycoside **5** (c) and comparison of [**4**][(BF_4)$_4$] at room temperature (e) with heating at 80 °C (d) or addition of glycoside **5** (f).

Our interpretation of these data is that in DMSO **4** exists as a library of conformers (rotation of urea's) that are in slow exchange on the NMR time scale. At elevated temperatures the exchange is faster and/or less species are populated; both result is less complicated NMR spectra. The fact that the spectra were more resolved after adding an excess of *n*-octyl-β-D-glucoside is highly indicative of binding, where the host-guest complex has far less conformers. Nevertheless, a firm characterization of binding in DMSO could not be achieved due to the complexity of the spectra at the beginning of the titrations.

Section S3c. Titration procedures with the formation procedures of [4][BAr^F]$_4$ in CD_3CN with 3 or 9% water

Procedure A:

To a solution of the 49.7 mM palladium BAr^F solution (24 µL, 1.2 µmol, 1 eq.) with CD_3CN (558 µL) and H_2O (18 µL) in a NMR tube was added the dipyridyl ligand (0.7 mg, 2.01 µmol, 1.68 eq.) to afford a 0.83 mM cage solution in CD_3CN with 3% H_2O of a total volume of 600 µL. After equilibration over 36 h, known aliquots of the guest stock solution in CD_3CN with 3% H_2O were added to the host solution and after each addition a ^{1}H NMR was spectra was taken. Aliquots: 1, 2, 4, 8, 16, 32, 64, 128, 256 and 256 µL.

Procedure B:

Identical method A, but generating a 0.83 mM cage solution in CD_3CN with 9% H_2O of a total volume of 600 µL (adding 522 µL CD_3CN and 54 µL H_2O).

Procedure C:

Guests D-glucose and β-methyl-*N*-acetyl-glucosamine were added as 0.2 M stock solutions in H_2O, due to their very low solubility in CD_3CN with 9% H_2O.

To a solution of palladium BAr^F (300.3 µL, 15 µmol, 1 eq.) with CD_3CN (9709.7 µL) and H_2O (660 µL) in a 20 mL vial was added dipyridyl ligand (7.7 mg, 22 µmol, 1.47 eq.) and the resulting 0.50 mM stock solution was equilibrated over 36 h. For every 'titration' point a separate NMR tube was prepared and a known aliquot of 0.2 M guest solution and H_2O were added as described in **Table S3-1**, keeping the percentage of water consistent during the titration.

Table S3-1. Used aliquots of [**4**][BAr^F]$_4$ solution, guest solution and H_2O for every titration point.

'Titration' point	µL cage solution	µL H_2O in cage solution	µL guest solution	µL H_2O	µL solvent total	Theoretical guest eq.	mM guest
1	970	60	0	29	999	0	0
2	970	60	1	29	1000	0.4	0.2
3	582	36	1.2	16.8	600	0.8	0.4
4	582	36	3	15	600	2	1
5	582	36	6	12	600	4	2
6	582	36	9	9	600	6	3
7	582	36	12	6	600	8	4
8	582	36	15	3	600	10	5
9	582	36	18	0	600	12	6

Section S3d. Dilution study of [4][BAr^F]$_4$ in CD_3CN with 3% water

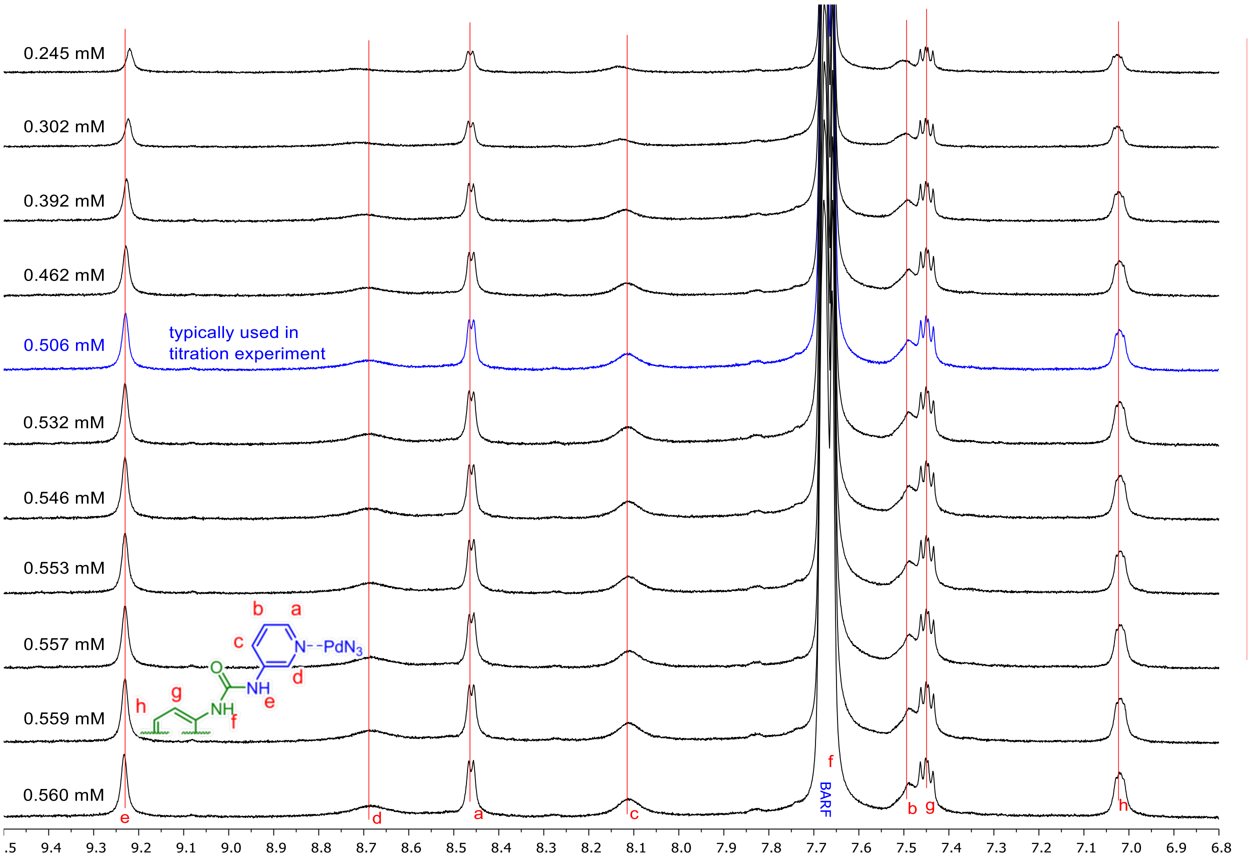

Figure S3-3. ^{1}H-NMR spectra of [**4**][(BAr^F)$_4$] at room temperature in the indicated concentration range of 0.560 – 0.245 mM in acetonitrile containing 3% of water. The vertical dashed red lines are added as a guide to the eye to emphasize that no peak-shifting was observed. This is highly indicative of a monomeric form of [**4**][(BAr^F)$_4$] under the conditions used in titration experiments. The spectrum highlighted in blue at about 0,5 mM is the most frequently used concentration of the **4** during a titration.

Section S3e. Studies of the binding of [4][BAr^F]$_4$ in CD_3CN with 3 or 9% H_2O

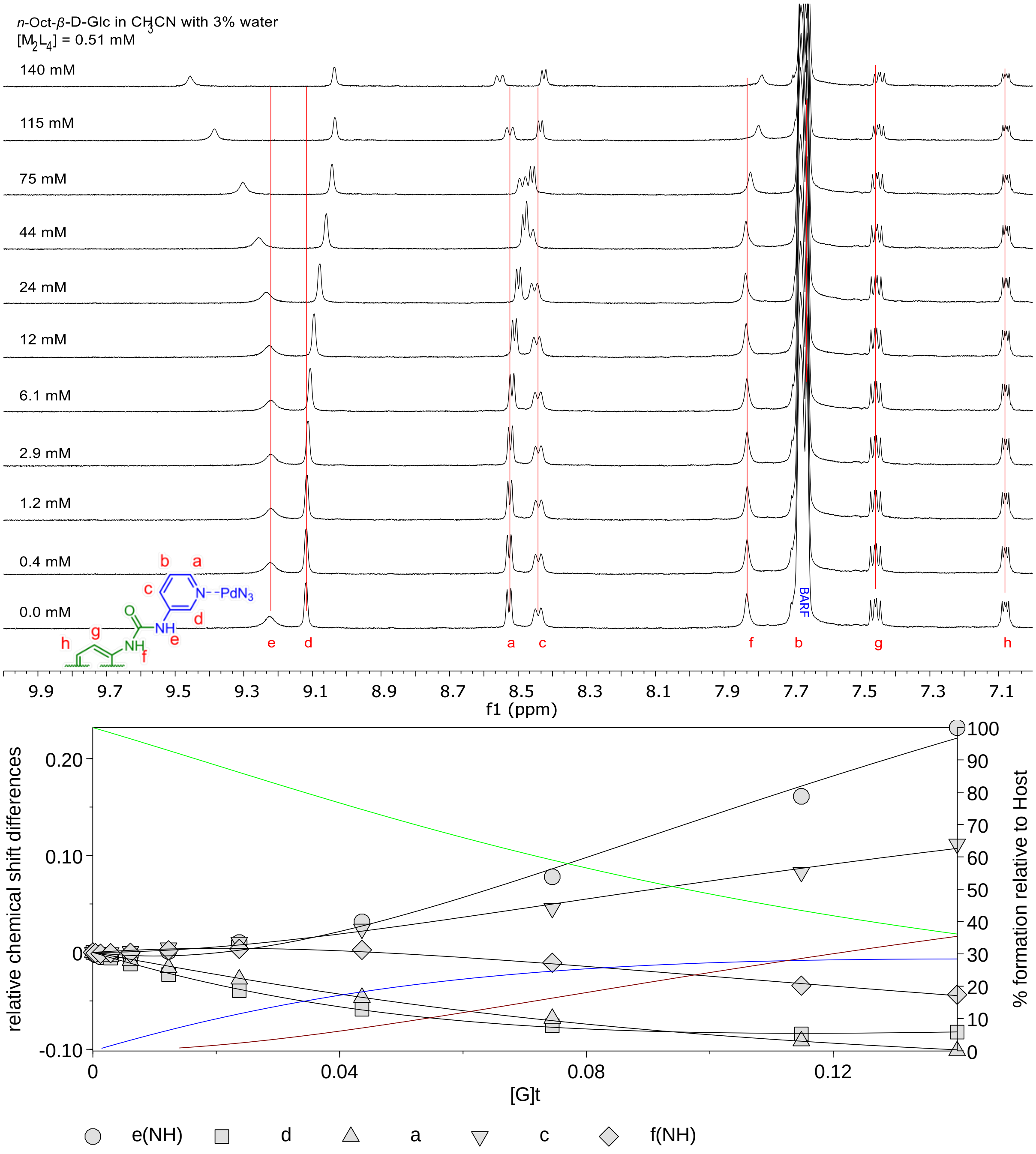

Figure S3-4. Top: ^{1}H NMR spectra and assignment of a binding study of cage [**4**][(BAr^F)$_4$] with *n*-octyl-β-D-glucoside (**5**) using **procedure A**. The guest stock solution concentration was 0.25 M, where a maximum concentration of about 140 mM of guest could be achieved in the titration. Initial concentration of host = 0.51 mM. The vertical red dashed lines were added as a guide to the eye. Bottom: HypNMR binding analysis following proton signals a, c-f of the cage. The fit was obtained assuming a 1:2 stoichiometry with $K_a^{1:1}$ = 6 M^{-1} and $K_a^{1:2}$ = 9 M^{-1}. The fit over all 55 datapoints can be seen as good with r^2 = 0.9947. The modelled species distributions is also shown as colored lines with 'Host' = green, 'Host-Guest' = blue, and 'Host-Guest$_2$' = brown.

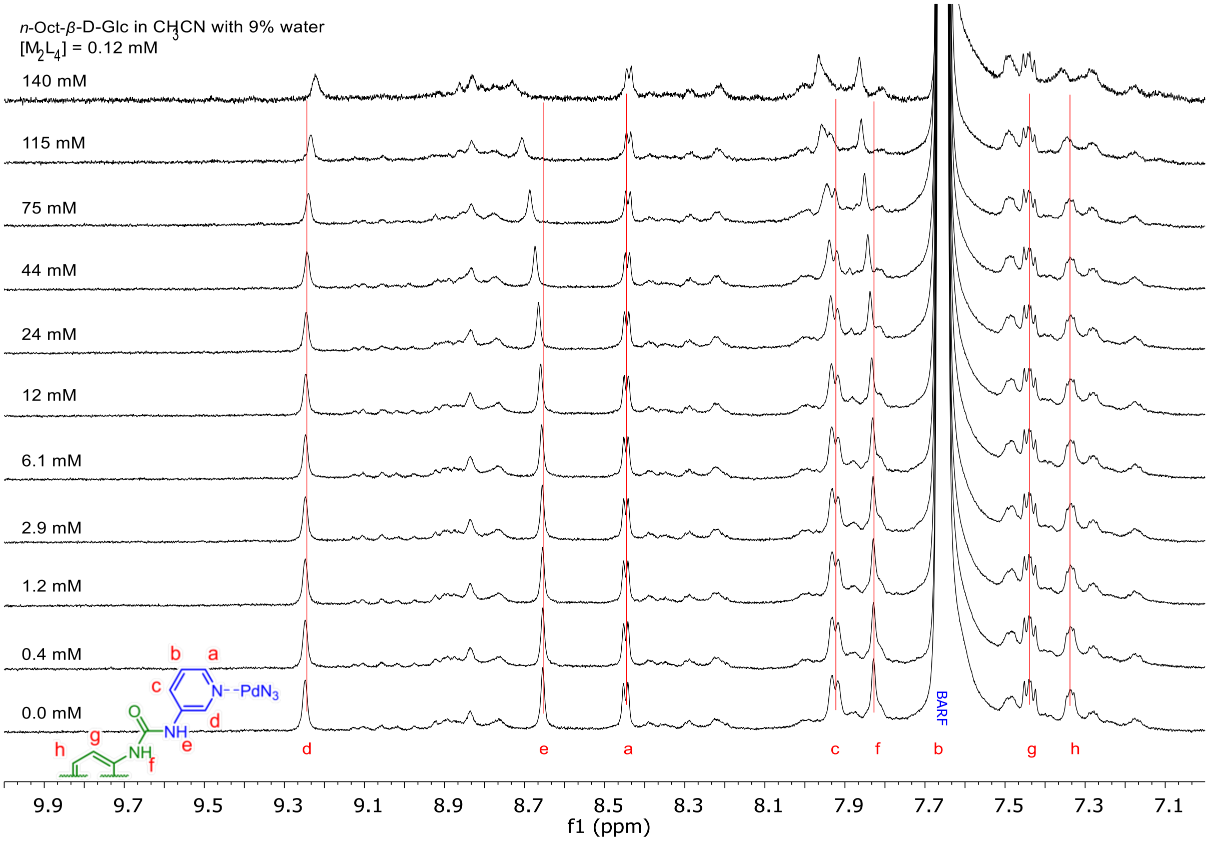

Figure S3-5. ^{1}H NMR spectra and assignment of a binding study of [**4**][(BAr^F)$_4$] with *n*-octyl-β-D-glucoside (**5**) using **procedure B**. The guest stock solution concentration was 0.25 M, where a maximum concentration of about 140 mM of guest could be achieved in the titration. Initial concentration of host = 0.12 mM. The vertical red dashed lines were added as a guide to the eye. While this particular sample appears more complicated than typically observed, the resonances belonging to the cage can clearly be distinguished and could be followed (likely cage deterioration or incomplete formation). That hardly any shifts were observed in this matrix (9% water in acetonitrile) is unsurprising given that the binding constant in the less competitive 3% water in acetonitrile is already very low (order of ~10 M^{-1}, see Figure S3-4).

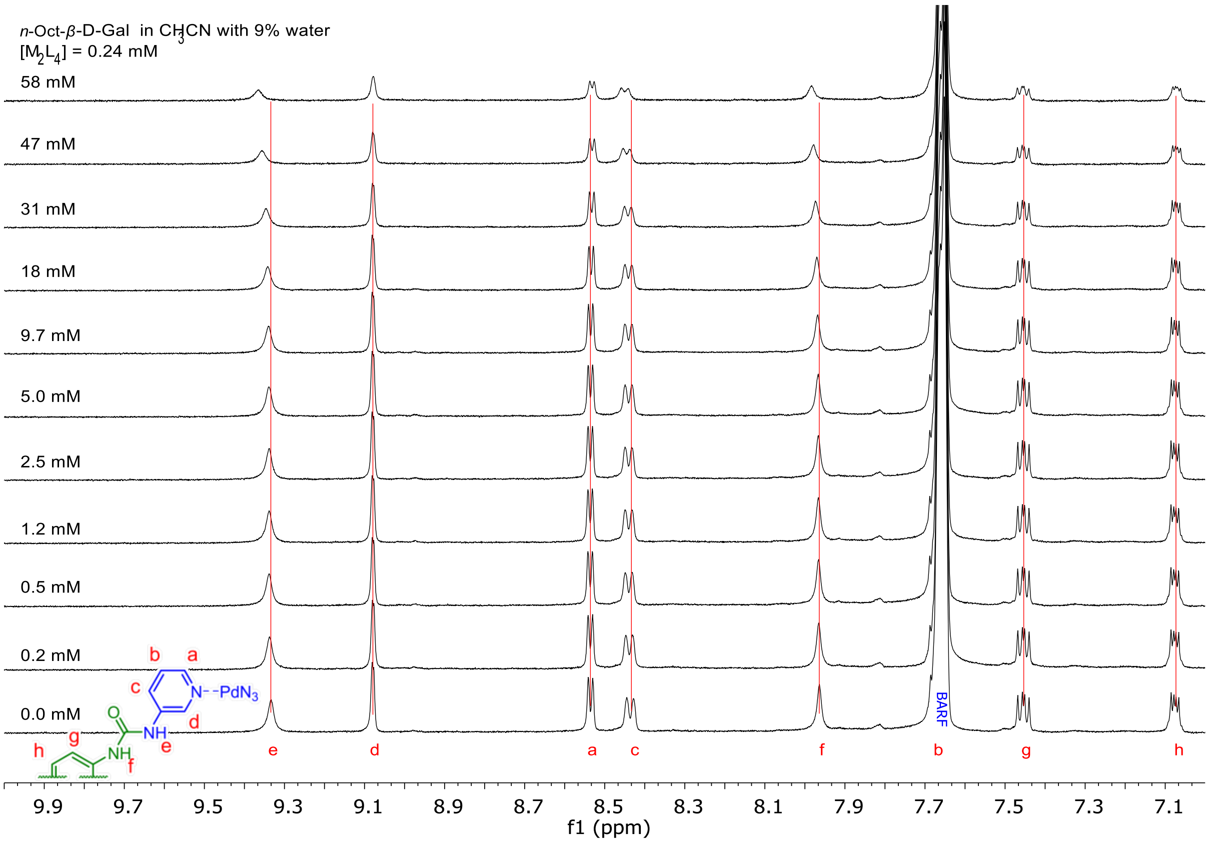

Figure S3-6. ^{1}H NMR spectra and assignment of a binding study of cage [**4**][(BAr^F)$_4$] with *n*-octyl-β-D-galactoside (**6**) using **procedure B**. The guest stock solution concentration was 0.10 M, where a maximum concentration of about 60 mM of guest could be achieved in the titration. Initial concentration of host = 0.24 mM. The vertical red dashed lines were added as a guide to the eye.

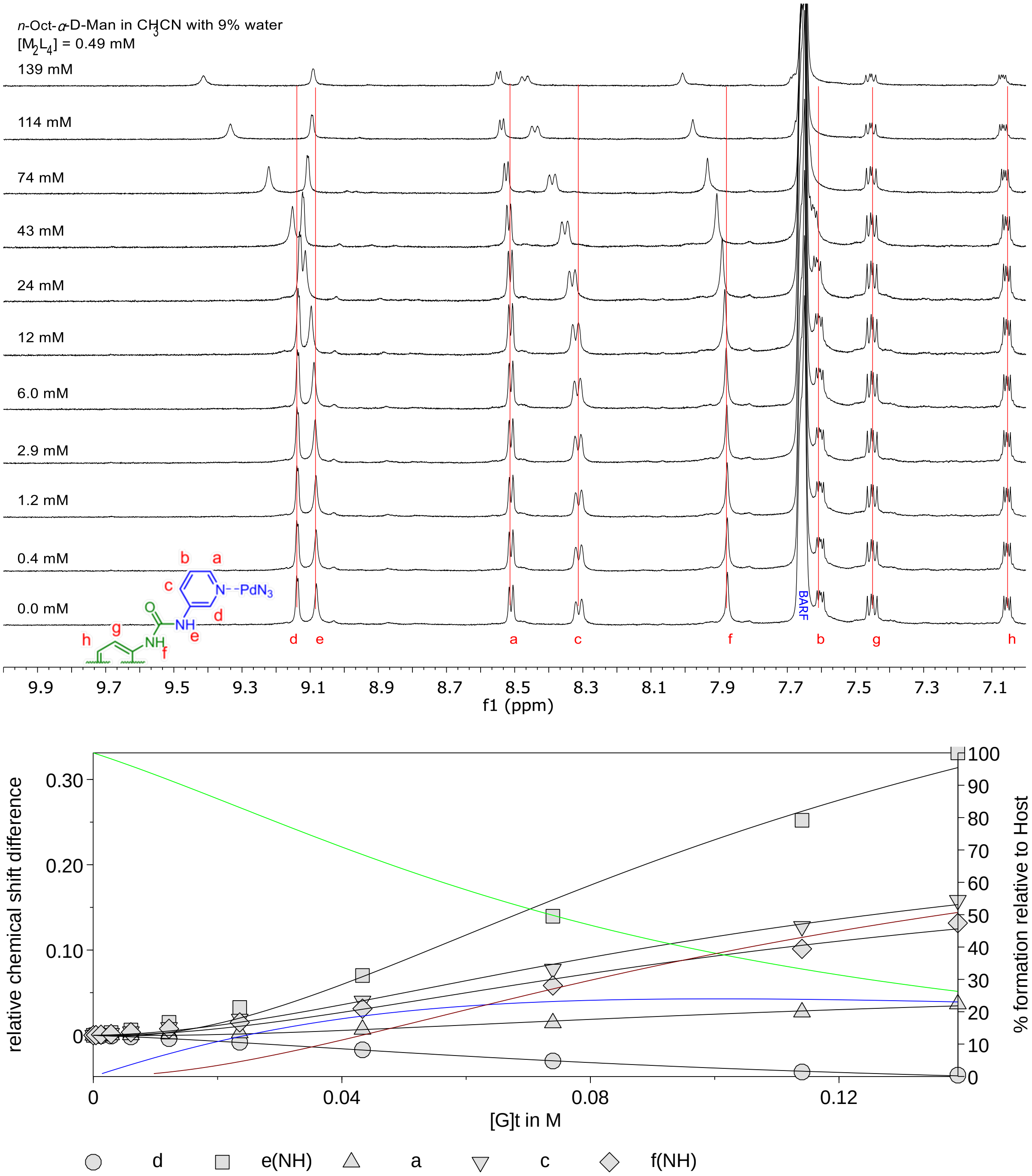

Figure S3-7. Top: ^{1}H NMR spectra and assignment of a binding study of [**4**][(BAr^F)$_4$]with *n*-octyl-α-D-mannoside (**7**) using **procedure B**. The guest stock solution concentration was 0.25 M, where a maximum concentration of about 140 mM of guest could be achieved in the titration. Initial concentration of host = 0.49 mM. The vertical red dashed lines were added as a guide to the eye. Bottom: HypNMR binding analysis following proton signals and c-f of the cage. The fit was obtained assuming a 1:2 stoichiometry with $K_a^{1:1}$ = 6 M^{-1} and $K_a^{1:2}$ = 16 M^{-1}. The fit over all 55 datapoints can be seen as good with r^2 = 0.9922. The modelled species distributions is also shown as colored lines with 'Host' = green, 'Host-Guest' = blue, and 'Host-Guest$_2$′ = brown.

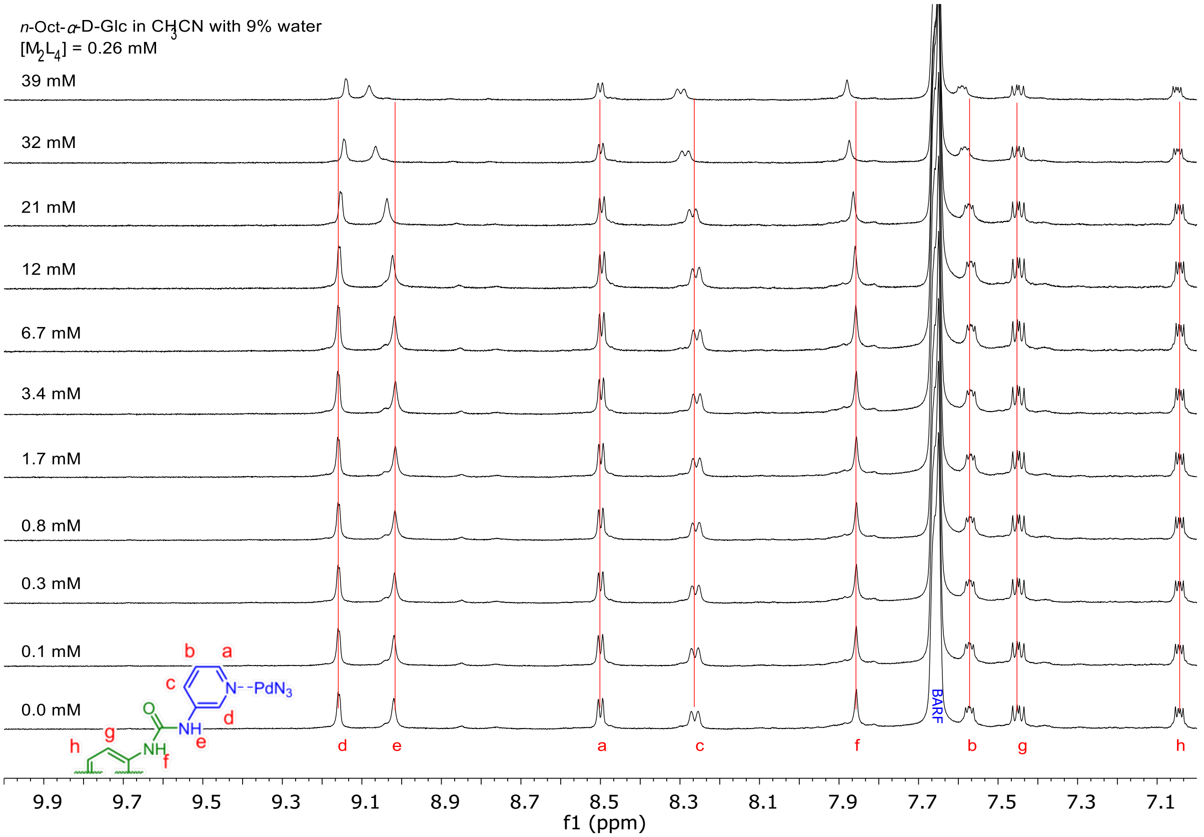

Figure S3-8. ^{1}H NMR spectra and assignment of a binding study of [**4**][(BAr^F)$_4$] with *n*-octyl-α-D-glucoside (**8**) using **procedure B**. The guest stock solution concentration was 0.07 M, where a maximum concentration of about 40 mM of guest could be achieved in the titration. Initial concentration of host = 0.26 mM. The vertical red dashed lines were added as a guide to the eye.

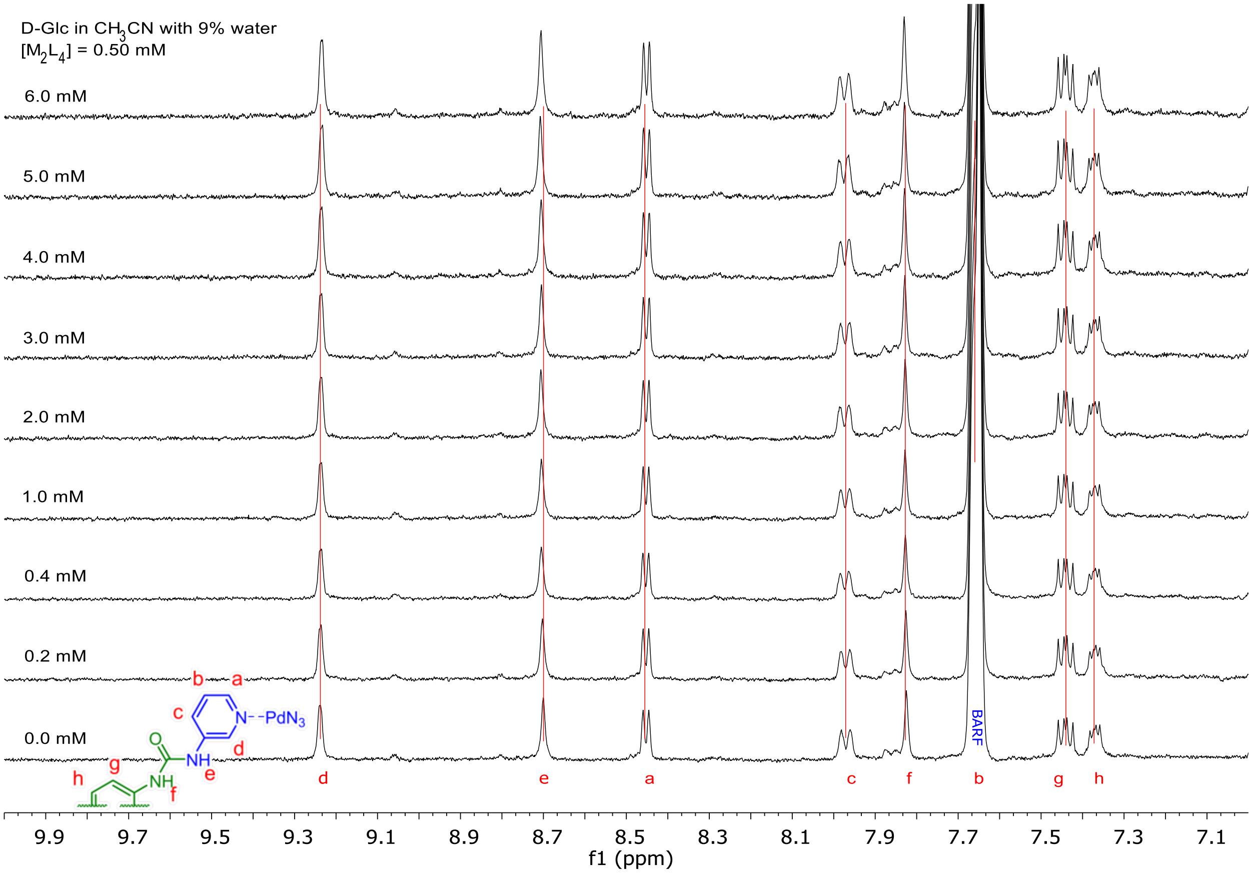

Figure S3-9. ^{1}H NMR spectra and assignment of a binding study of [**4**][(BAr^F)$_4$] with D-glucose (**9**) using **procedure C**. The guest stock solution concentration was 0.20 M, where a maximum concentration of about 6 mM of guest could be achieved in the titration. Initial concentration of host = 0.50 mM. The vertical red dashed lines were added as a guide to the eye.

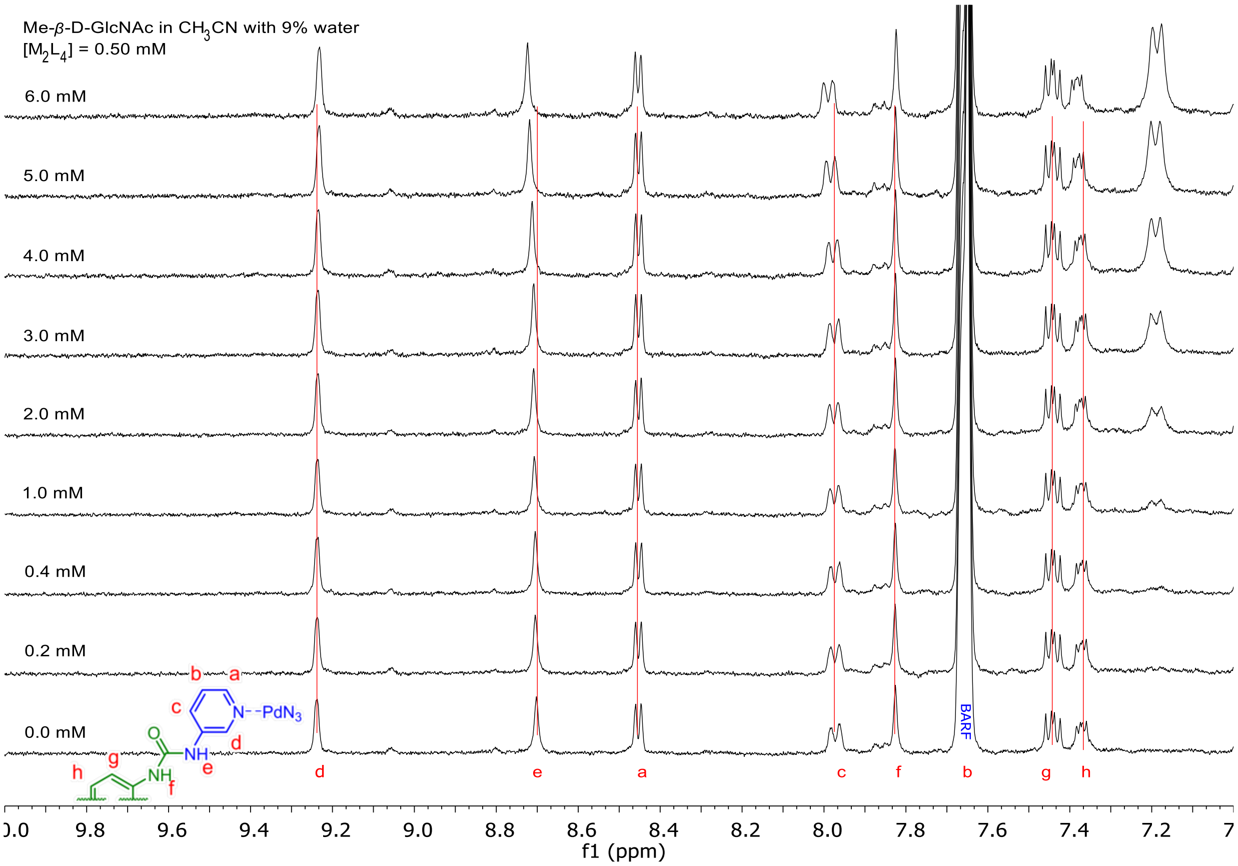

Figure S3-10. ^{1}H NMR spectra and assignment of a binding study of [**4**][(BAr^F)$_4$] with Me-β-D-GlcNAc (**10**) using **procedure C**. The guest stock solution concentration was 0.20 M, where a maximum concentration of about 6 mM of guest could be achieved in the titration. Initial concentration of host = 0.50 mM. The vertical red dashed lines were added as a guide to the eye.

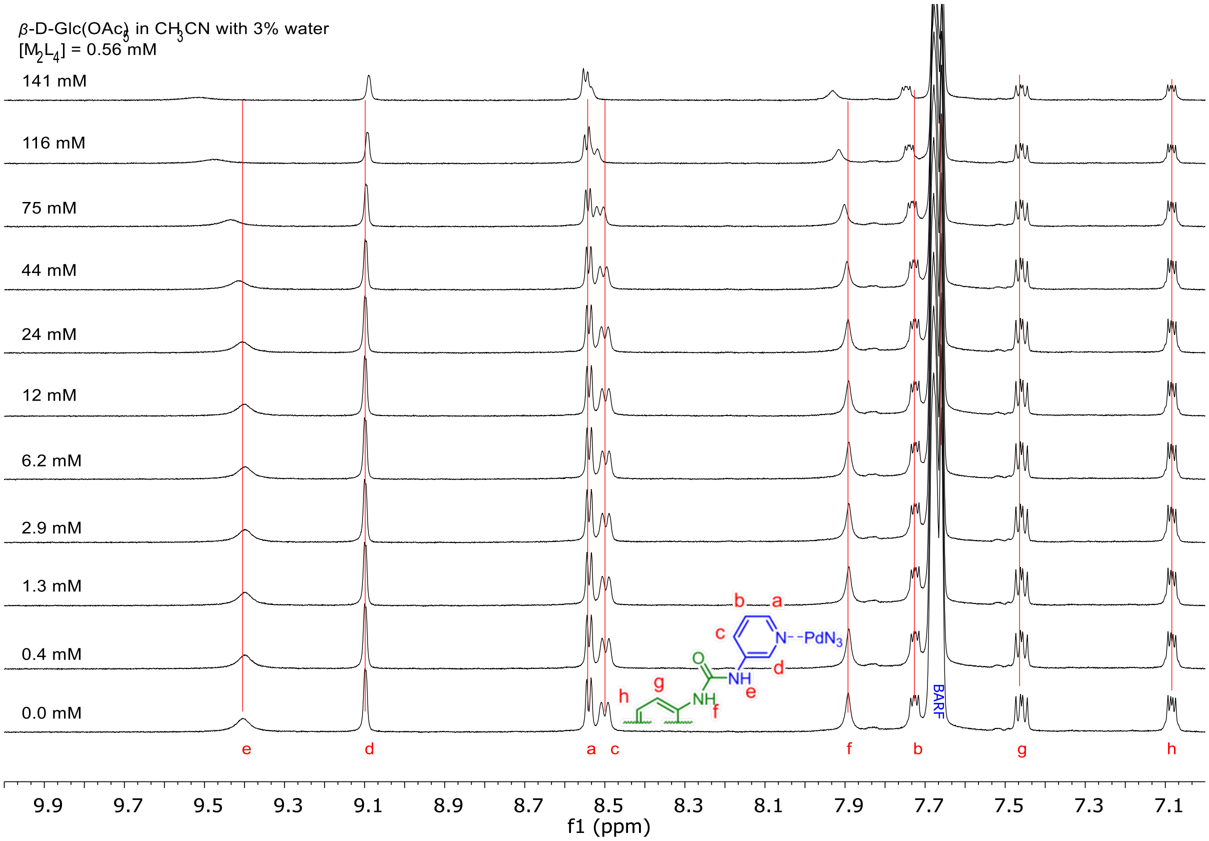

Figure S3-11. ^{1}H NMR spectra and assignment of a binding study of [**4**][(BAr^F)$_4$] with β-D-Glc(OAc)$_5$ (**11**) using **procedure A**. The guest stock solution concentration was 0.25 M, where a maximum concentration of about 140 mM of guest could be achieved in the titration. Initial concentration of host = 0.56 mM. The vertical red dashed lines were added as a guide to the eye.

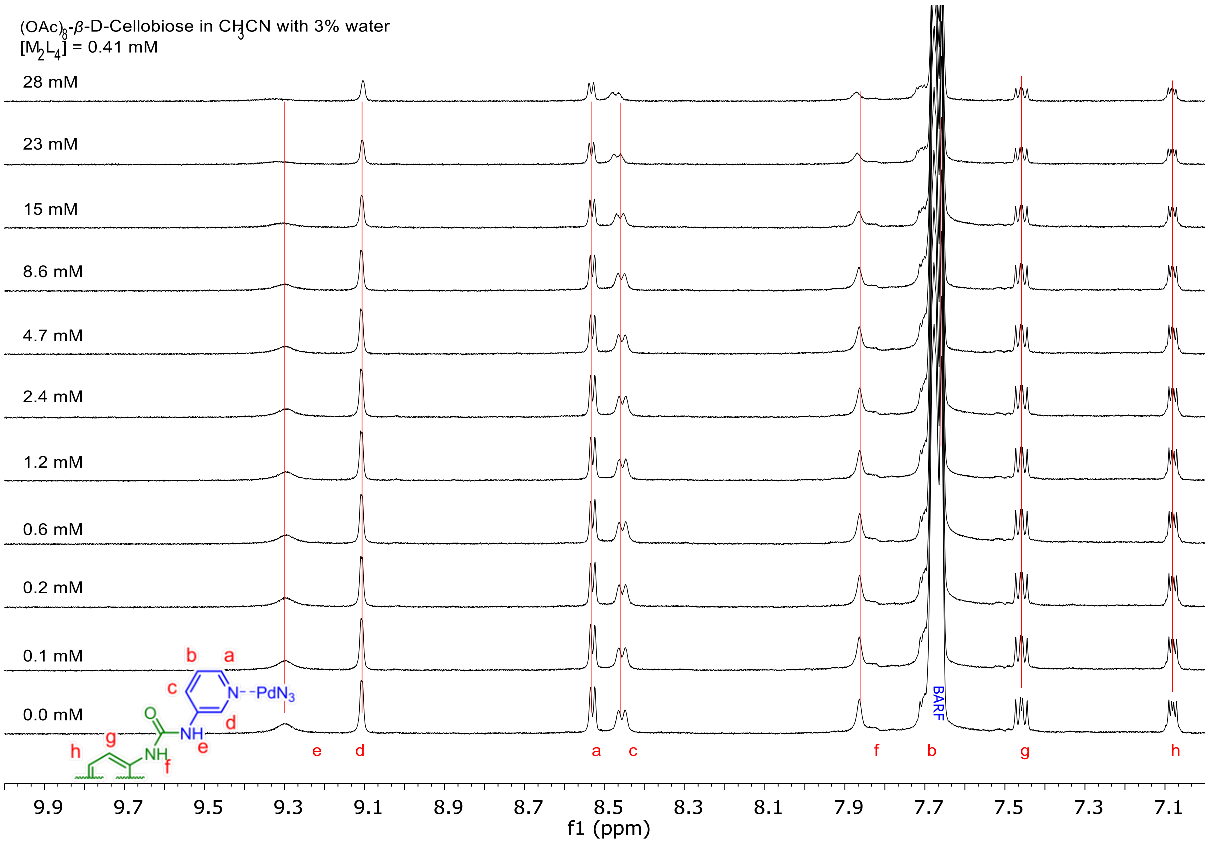

Figure S3-12. ^{1}H NMR spectra and assignment of a binding study of [**4**][(BAr^F)$_4$] with β-D-cellobiose(OAc)$_8$ (**12**) using **procedure A**. The guest stock solution concentration was 0.05 M, where a maximum concentration of about 30 mM of guest could be achieved in the titration. Initial concentration of host = 0.41 mM. The vertical red dashed lines were added as a guide to the eye.

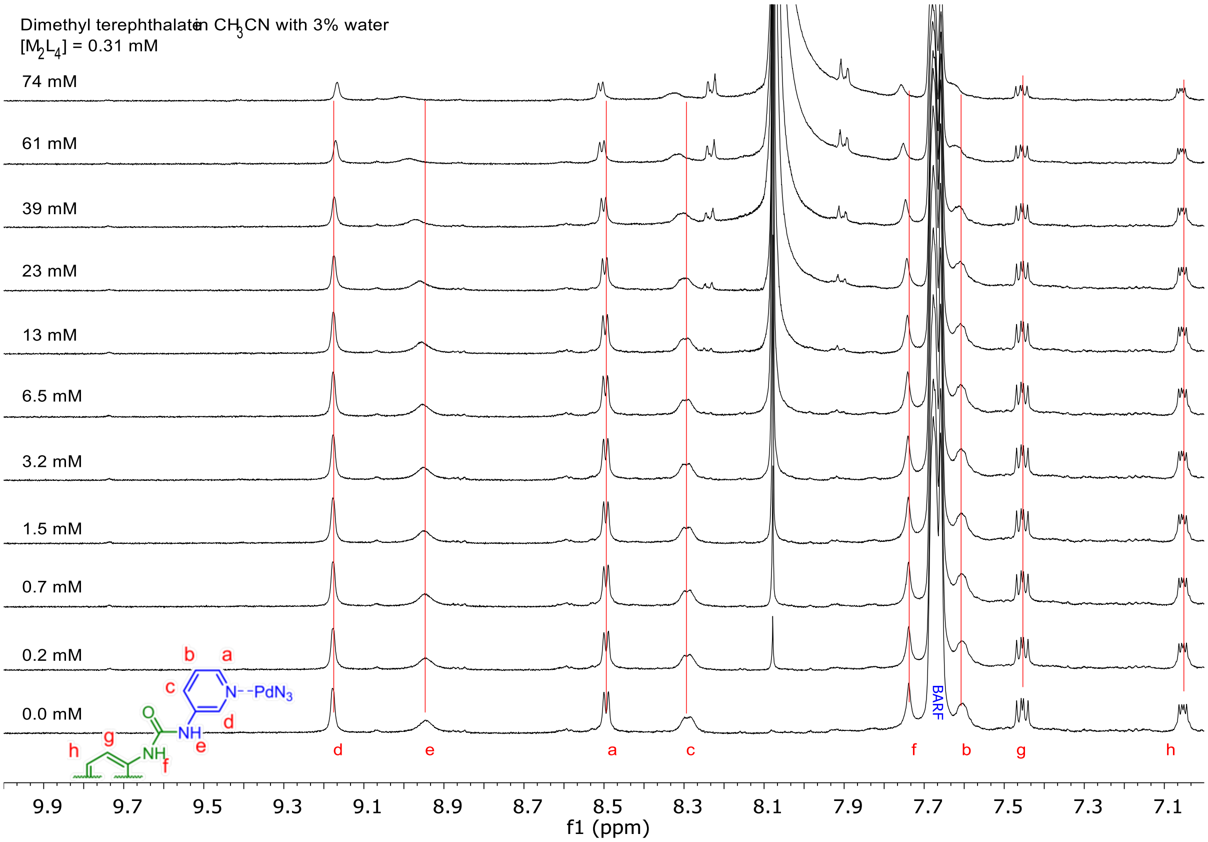

Figure S3-13. ^{1}H NMR spectra and assignment of a binding study of [**4**][$(BAr^F)_4$] with dimethylterephthalate (**13**) using **procedure A**. The guest stock solution concentration was 0.13 M, where a maximum concentration of about 75 mM of guest could be achieved in the titration. Initial concentration of host = 0.31 mM. The vertical red dashed lines were added as a guide to the eye.

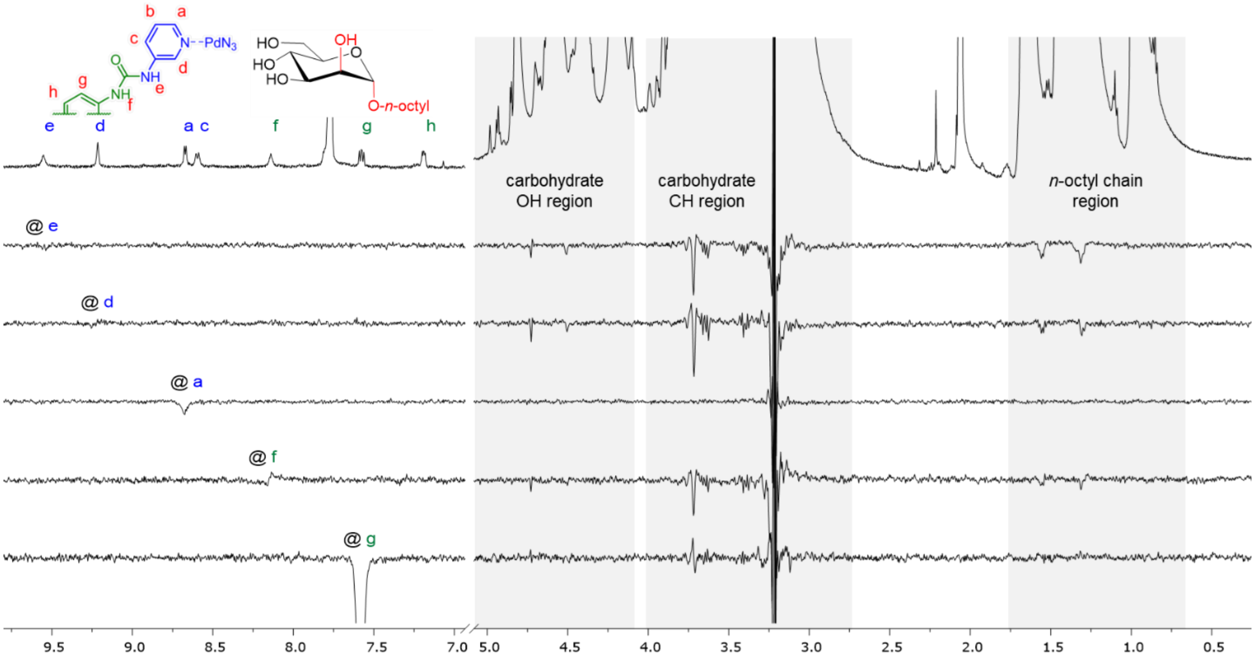

Figure S3-14. 1D Selective nOe studies of the final solution of the titration of [**4**][(BAr^{F})$_4$] with *n*-octyl-α-D-mannoside **7** with t_m = 500 ms (see Figure S3-7 for titration). Irradiation of the inwards facing protons **d**, **e** and to some extend **f** result in clear nOe signals in the carbohydrate regions. Contrary, irradiation of the outwards facing proton **a** (and to some extend **g**) has no nOe with the carbohydrate region (the large peak around 3.25 p.p.m. is water).

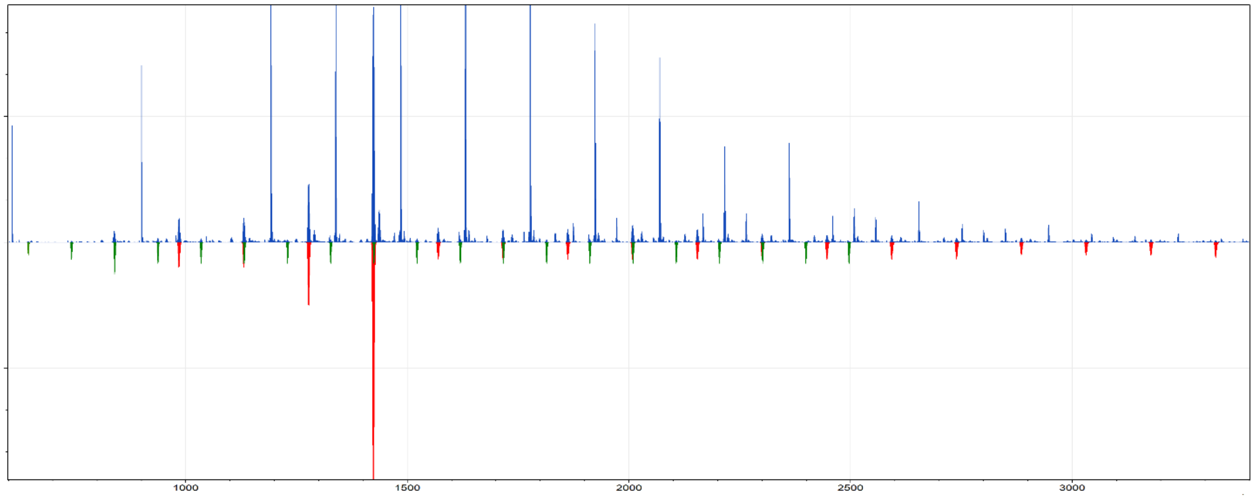

Figure S3-15. Full CSI HRMS spectrum of the solution after the titration of forming [**4**⊂**5**][(BAr^F)$_4$] in CD_3CN with 3% H_2O (top in blue). The bottom green depicts the calculated [**4**⊂**5**][Cl]$^{3+}$ with stoichiometries from 1 to 20 of **5** : **4** and the bottom red shows the calculated [**4**⊂**5**][Cl$_2$]$^{2+}$ with stoichiometries from 1 to 20 of **5** on **4**. The BAr^F species have not been observed and the chloride anions were present in the solvent and machine for mass spectrometry. Also *n*-octyl-β-D-glucoside clusters were observed. The fits to this spectrum is given in Table S3-2 below.

Table S3-2. Observed (Obs.) and calculated (Calc.) *m/z* ratio's with deviations (Δ in p.p.m.) of mass peaks observed in the CSI HRMS spectrum of [**4**⊂**5**][(BAr^F)$_4$] in CD_3CN with 3% H_2O (see Figure S3-15).

	m/z for [**4**][Cl]$^{3+}$			*m/z* for [**4**][Cl$_2$]$^{2+}$		
Eq. **5**	Obs.	Calc.	Δ	Obs.	Calc.	Δ
0	547.1132	547.1036	17.55	838.1323	838.1397	-8.83
1	644.5064	644.4999	10.09	984.2245	984.2343	-9.96
2	741.8959	741.8963	-0.54	1130.8170	1130.8292	-10.79
3	839.2859	839.2927	-8.10	1276.9111	1276.9237	-9.87
4	937.0135	937.0226	-9.71	1423.5039	1423.5182	-10.05
5	1034.4082	1034.4190	-10.44	1569.5968	1569.6128	-10.19
6	1131.8161	1131.8153	0.71	1715.6895	1715.7073	-10.37
7	1229.1999	1229.2117	-9.60	1861.7820	1861.8019	-10.69
8	1326.5948	1326.6080	-9.95	2007.8749	2007.8965	-10.76
9	1424.3229	1424.3389	-11.23	2153.9669	2153.9910	-11.19
10	1521.7190	1521.7342	-9.99	2300.0605	2300.0855	-10.87
11	1619.1148	1619.1306	-9.76	2446.1517	2446.1801	-11.61
12	1716.5101	1716.5269	-9.79	2592.7437	2592.7749	-12.03
13	1813.9049	1813.9232	-10.09	2738.8354	2738.8694	-12.41
14	1911.2988	1911.3196	-10.88	2884.9269	2884.9640	-12.86
15	2008.6947	2008.7159	-10.55	3031.0174	3031.0585	-13.56
16	2106.4230	2106.4459	-10.87	3177.1105	3177.1530	-13.38
17	2203.8193	2203.8422	-10.39	3323.2033	3323.2475	-13.30
18	2301.2311	2301.2385	-11.91			
19	2398.6068	2398.6349	-11.71			
20	2496.3364	2496.3648	-11.38			

Section S4. Modeling of 4 and its *n*-octyl-glycoside complexes

All calculations were performed using Spartan 2016 with molecular mechanics (MMFF force field) or density functional theory (DFT) at the ωB97X-D / 6-31G* level of theory. The dispersion correction was applied because it is known that dispersion forces can be significant in inclusion complexes, especially those containing aromatic rings.[3]

Section S4a. Unbound cage 4

To estimate what the energy minimum conformer might be, several structures were considered based on the relative orientation of the (most flexible) urea groups. An overview of this procedure is illustrated in Figure S4-1.

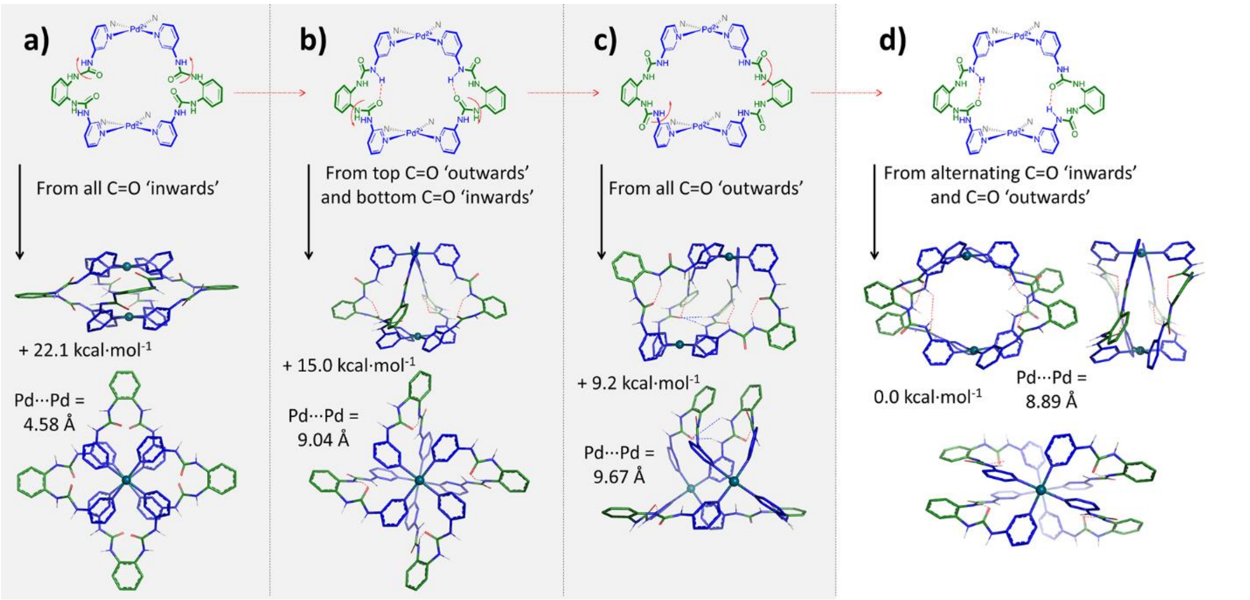

Figure S4-1. Overview of computed conformers, starting from geometries where the urea carbonyl groups as pointing 'inwards' or 'outwards' relative to the center of mass of **4** (as indicated in the top). In the initial structures, all torsion angles were constrained so that each bis-pyridyl ligand was completely flat. That structure was then subjected to an unconstrained geometry optimization using density functional theory at the ωB97X-D / 6-31G* level of theory. The resulting structures are viewed perpendicular to the Pd-Pd axis (middle) and along the Pd-Pd axis (bottom). The relative energies (in kcal·mol^{-1}) as well as the Pd···Pd distance (in Å) of each conformer is shown in the figure.

The urea carbonyl O's can be seen pointing 'inwards' or 'outwards' relative to the cage's center of mass. The conformer where all carbonyls point 'inwards' resulted in a structure without any interior void, as is obvious from structures shown in Figure S4-1a and the very short Pd···Pd distance of 4.58 Å. As is shown in Figure S4-1b for another conformer all four urea's on one PdN_4 side were oriented 'inwards', while the other four were oriented 'outwards'. This facilitates the formation of four intramolecular hydrogen bonds (indicated by red dashed lines). This structure converged into a symmetrical structure with internal void (Pd···Pd = 9.04 Å) and is about 5 kcal·mol^{-1} more stable than the 'all-inwards' structure. Considering a

structure where all carbonyl O's are pointing 'outwards', as is shown in Figure S4-1c, converged into a structure where four intramolecular hydrogen bonds were established and which was about 5 kcal·mol^{-1} more stable than the 'top-outwards/bottom-inwards' structure (Figure S4-1b). In addition, two of the phenyl rings were in close proximity to one another and held in place by an additional hydrogen bonding interaction between two urea groups of different ligands (blue dashed lines). This conformer has an interior void, as can be seen in Figure S4-1c and can also deduced from the Pd···Pd distance of 9.67 Å. In the final conformer shown in Figure S4-1d, the urea carbonyls were oriented inwards and outwards in an alternating fashion as to facilitate the formation of four intramolecular hydrogen bonds (red dashed line). This structure converged with an energy at least 9 kcal·mol^{-1} more stable the other conformers. The four anticipated intramolecular hydrogen bonds were indeed formed, and two sets of ligand phenyl rings are in close proximity (presumably due to dispersion driven stacking-like interactions). The Pd···Pd distance is 8.89 Å and the complex clearly has an open structure with a hollow interior.

An implication of the relative energies found for the conformers show in Figure S4-1 is that the most stable structure shown in Figure S4-1d is practically fully populated at 25 °C when applying Boltzmann's equation (a 5 kcal·mol^{-1} difference already gives 99.98% population). Whether this modelling divulged the true energy minimum conformer is not certain however. For example: solvation is a notoriously difficult parameter to account for, particularly when working in a solvent mixture. Nevertheless, the fact that three out of four structures considered present an open cavity –as opposed to a collapsed and thus inaccessible structure– installs confidence that the cage can act as a host for small molecules.

Shown in Figure S4-2a is a space-filling representation of the most stable conformer, together with distances D1-D4 to estimate the internal dimensions of the structure (see caption for details). The height of the interior is at least 3.4 Å (D3) and at most about 5.4 Å (D1). This distance range is on par with the interior height of amide-linked covalent carbohydrate receptors (3.9 Å) and a related urea- linked glucose receptor (5.5 Å).[4] The width of the cage is about 6.8 Å (D2) which is complementary to the smallest width of a carbohydrate such as n-octyl-α-D-mannoside (7.7 Å, see Figure S4-2b). This complementarity is particularly clear when accounting for ~0.5 Å van der Waals overlap for hydrogen bonding interactions on both sides of the carbohydrate (7.7 – (2 x 0.5) = 6.7 Å). The diagonal distance D4 of 4.1 Å is also fairly large. It can be noted that D4 involves two unbound urea NH protons (e) pointing towards each other with nearly linear N–H···H angles as well as two carbonyl O's pointing towards the cage's interior. Such an arrangement of N–H hydrogen bond donors and C=O hydrogen bond acceptors might be beneficial to form hydrogen bonds with carbohydrate's hydroxyl circumference. The measurements of these models thus suggest that it is at least sterically feasible for the cage to host a carbohydrate.

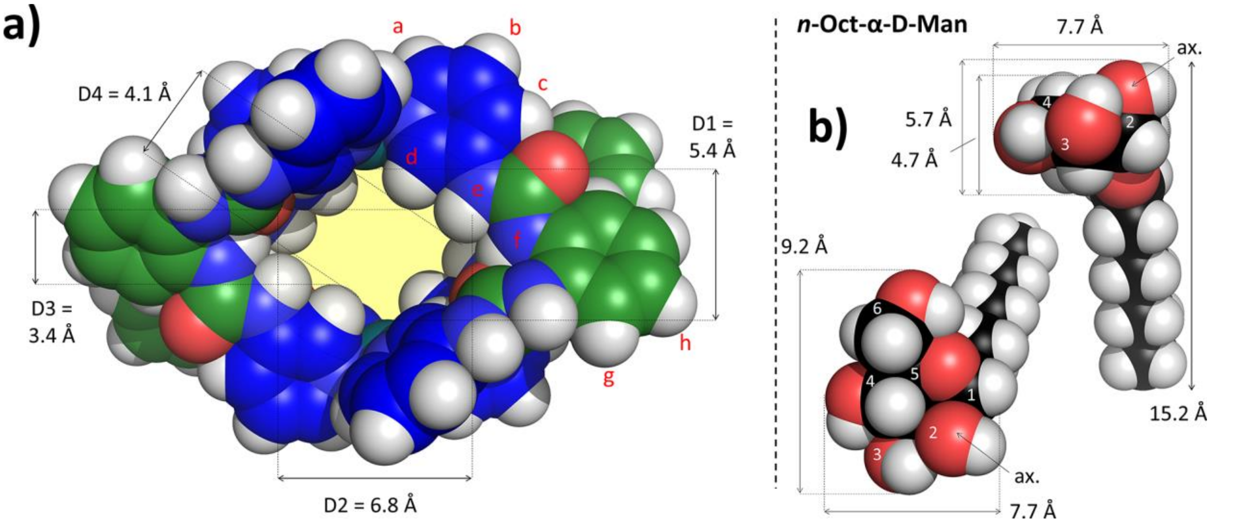

Figure S4-2. Space filling representations of the most stable conformer (DFT / ωB97X-D / 6-31G*) of: **4** computed (see also Figure S4-1) with some dimensions of the cage's interior (**a**) and of *n*-octyl-α-D-mannoside with some relevant dimension (**b**). The given dimensions are based on measurements between relevant atoms, centroids or planes and using the van der Waals radii of C (1.70 Å), O (1.52 Å), H (1.09 Å) and Pd (1.63 Å). D1 = [Pd···Pd distance] – 2 x 1.63 Å; D2 = [distance between the centroid of two protons f and two inwards facing carbonyl O's on one 'side' of the cage to a similar 'f_2O_2' centroid at the opposite side of the cage] – 2 x 1.09 Å; D3 = [distance between a centroid of four protons d on one side of the cage to a similar 'd_4' centroid at the opposite side of the cage] – 2 x 1.09 Å; D4 = [the average distance from one centroid between protons d and e to the opposite 'de' centroid, using only the inwards pointing protons e] – 2 x 1.09 Å. The given dimensions of *n*-octyl-α-D-mannoside were obtained in a similar manner.

Section S4b. Cage 4 in complex with carbohydrates

To gain a (simple) model of possible binding modes between cage **4** and several carbohydrates, models were generated of the cage bound to the four *n*-octyl glycosides studied. The starting geometries were obtained by using the coordinates of the energy minimum cage (see Figure S4-1) and manually placing the carbohydrate within the cage's cavity (the carbohydrate geometry was obtained by a DFT conformational search). Due to the dimensions of the presumed binding pocket (Figure S4-2a), the carbohydrates were positioned so that their smallest width (~7.7 A, see Figure S4-2b) was complemented by the width of the cavity (6.8 Å, D2 in Figure S4-2a) and so that the carbohydrate's pyranose ring was coplanar with both PdN_4 planes. This implied that the *n*-octyl tails were oriented in between two phenyl rings of two dipyridyl ligands. After positioning the carbohydrate, a conformational search (MMFF) of the complex was performed by allowing the hydroxyl OH bonds as well as the carbohydrate C5-C6 bond to undergo a fourfold rotation (giving a library of 1024 candidates). In all cases, this resulted in a minimum representing >80% of the Boltzmann distribution. The most stable conformer of this minimum was geometry optimized using unconstrained DFT (ωB97X-D / 6-31G*), resulting in the complexes shown in Figure S4-3.

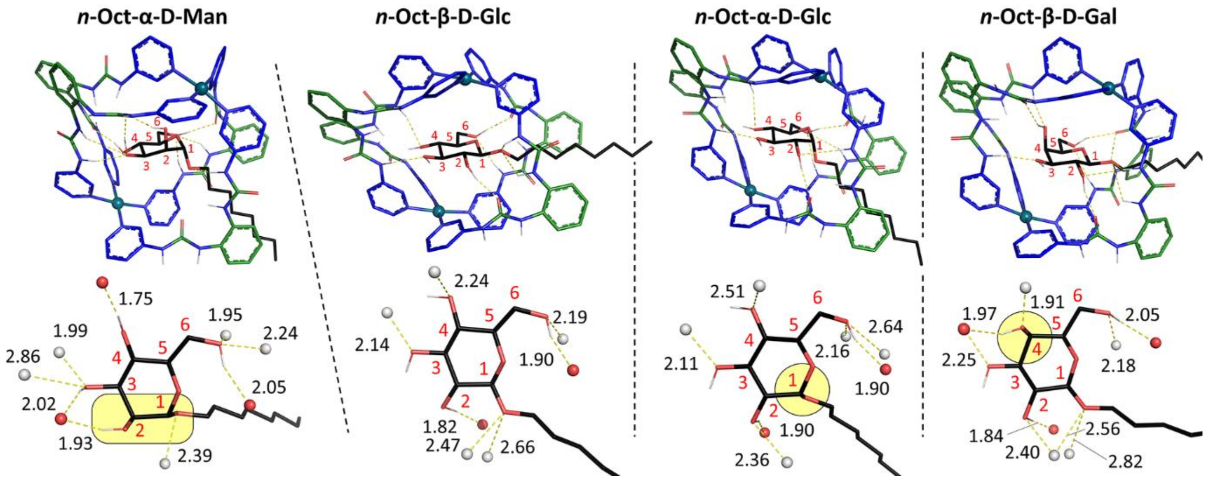

n-Oct-α-D-Man (9 HB)		*n*-Oct-β-D-Glc (7HB)		*n*-Oct-α-D-Glc (7HB)		*n*-Oct-β-D-Gal (9HB)	
moderate	weak	moderate	weak	moderate	weak	moderate	weak
1.75 (^{OH}4)	2.24 (^{OH}6)	1.82 (^{OH}2)	2.24 (^{OH}4)	1.90 (^{OH}2)	2.36 (^{OH}2)	1.84 (^{OH}2)	2.25 (^{OH}3)
1.93 (^{OH}2)*	2.39 (^{O}1)	1.90 (^{OH}6)	2.47 (^{O}1)	1.90 (^{OH}6)	2.51 (^{OH}4)	1.91 (^{OH}4)*	2.40 (^{OH}2)
1.95 (^{OH}6)	2.86 (^{OH}3)	2.14 (^{OH}3)	2.66 (^{O}1)	2.11 (^{OH}3)	2.64 (^{OH}6)	1.97 (^{OH}4)*	2.56 (^{O}1)
1.99 (^{OH}4)		2.19 (^{OH}6)		2.16 (^{OH}6)		2.05 (^{OH}6)	2.82 (^{O}1)
2.02 (^{OH}3)						2.18 (^{OH}6)	
2.05 (^{OH}6)							

Figure S4-3. Top: geometry optimized structures of **4** bound to the four *n*-octyl glycosides studied for binding. The carbohydrate C-atoms are numbered in red and the hydrogen bonds established are shown as yellow dotted lines. Bottom: zoom-in of the *n*-octyl glycoside with numbering and hydrogen bond distances in Å. The axial positions are highlighted with a light yellow background. The bottom table lists the distances divided in 'moderate' (1.5–2.2 Å) and 'weak' (>2.2 Å) and the hydrogen bonds with axial hydroxyls are shown in red and given an asterisk.[5] The angles between the carbohydrate furanose C_5O plane and the two N_4Pd^{2+} planes are 39.9° and 41.4° for the mannoside, 2.5° and 6.4° for the β-glucoside, 24.5° and 26.3° for the α-glucoside, and 17.7° and 20.8° for the galactoside.

The complexes illustrated in Figure S4-3 are shown from a perspective so that each carbohydrate has a similar orientation: with C1 at the far-right and C4 at the far left. What immediately stands out from these

models is that the carbohydrate is never coplanar with the two PdN_4 planes: i.e. the carbohydrates can be seen as nested in D4 rather than D2 (see Figure S4-2). This is least so for the all-equatorial *n*-Oct-β-D-Glc, where the angle between the furanose C_5O plane and the N_4Pd^{2+} planes are < 6.4° *versus* 17.7° – 41.4° for the other glycosides (see caption of Figure 4-3 for details). Also shown in the figure are 'top' views of the carbohydrates and their hydrogen bonding patterns with the cage's urea groups. The table lists these same distances, divided into 'moderate' (1.5–2.2 Å) and 'weak' (>2.2 Å).[5] What stands out from the hydrogen bonding patterns, is that the glycosides with an axial hydroxyl have two more hydrogen bonds than the two glycosides that don't (9 *vs* 7). Moreover, the axial hydroxyl of the mannoside forms one of the stronger hydrogen bonds with H···O = 1.93 Å. The axial hydroxyl of the galactoside is involved in two of its strongest hydrogen bonds (H···O = 1.91 and 1.97 Å). These data thus suggest that the cage might prefer *n*-octyl glycosides with an axial hydroxyl group. Actually, these data would suggest that the galactose should bind strongest. Unfortunately, due to the solubility limit we were unable to elevate the concentration of *n*-Oct-β-D-Gal above 60 mM in the solvent used (9% water in acetonitrile) and no binding analysis could be done. Is must be stressed however, that the calculations described above are relatively simple gas-phase models and that solvation and entropy have not been taken into account. This makes it impossible to draw reliable conclusions on the thermodynamics from there models to relate them to the binding studies. Rather, these models can best be seen as clear computational (DFT) proof that glycosides can be accommodated within the cage's interior.

Section S4c. Cartesian coordinates of models computed with DFT

Table S4-1. Cartesian coordinates of four conformers of **4** computed with DFT at the ωB97X-D, 6-31G* level of theory. See also Figure S4-1.

from all CO 'inwards'				from top CO 'outwards' and bottom 'inwards'				from all CO 'outwards'				from alternating CO 'inwards'/'outwards'			
	x	y	z		x	y	z		x	y	z		x	y	z
Pd	0.87	-1.4	1.583	Pd	5.029	-0.76	-1.09	Pd	-2.409	1.915	3.743	Pd	1.733	-2.733	3.048
Pd	-0.886	1.397	-1.591	Pd	-3.709	0.588	0.784	Pd	0.621	-3.235	-3.862	Pd	-1.735	2.732	-3.05
O	-4.224	2.116	2.063	O	-1	4.696	-0.209	O	5.252	-1.381	0.581	O	2.214	-2.226	-6.32
O	-3.296	-2.818	-2.805	O	3.841	6.181	0.107	O	5.416	1.367	-2.815	O	3.566	-3.243	-1.576
O	3.088	-0.291	-4.131	O	-2.12	-4.179	0.896	O	-2.229	0.71	-4.991	O	-1.767	0.407	4.737
O	-1.596	-4.762	-1.185	O	1.526	-6.987	-1.247	O	2.07	4.516	-1.035	O	-3.852	4.803	3.441
O	-3.457	-0.245	3.815	O	-0.609	0.598	4.707	O	2.485	1.921	3.252	O	0.834	3.727	5.931
O	4.577	-1.539	-1.778	O	3.912	-1.35	5.939	O	-5.454	3.149	-2.007	O	-0.125	4.937	1.139
O	2.732	2.938	3.238	O	-2.584	-0.137	-4.05	O	-2.443	-3.247	4.176	O	-1.682	-2.105	-4.301
O	2.151	4.609	0.767	O	1.411	0.501	-7.117	O	-2.39	-7.094	0.863	O	0.799	-6.304	-3.053
N	-4.604	-2.425	-0.94	N	-2.181	4.87	-2.169	N	3.251	1.333	-3.564	N	1.658	-1.064	-4.417
N	-2.598	3.398	3.09	N	-0.794	6.584	-1.485	N	2.948	-1.394	0.552	N	3.659	-2.323	2.385
N	1.9	-2.261	-4.363	N	-3.885	1.901	-0.809	N	-3.001	-0.525	-6.765	N	5.611	-2.639	-0.721
N	1.528	-2.717	0.127	N	3.081	4.07	-0.395	N	-1.478	3.259	2.467	N	5.454	-3.544	-2.834
N	2.692	-0.422	1.518	N	1.584	5.781	-0.117	N	-3.875	1.646	2.307	N	3.049	-2.883	-4.279
N	-0.915	-2.436	1.716	N	5.326	1.234	-0.69	N	-0.927	2.232	5.163	N	-0.72	1.678	-4.495
N	-4.444	-1.688	2.301	N	-2.404	-4.104	3.174	N	2.658	1.537	5.499	N	-3.023	-1.44	5.273
N	0.127	-4.544	-2.711	N	-1.954	-6.108	2.118	N	1.851	3.747	1.115	N	-2.709	3.718	-1.531
N	5.141	0.567	-1.006	N	-3.606	-0.712	2.394	N	-4.021	1.476	-1.346	N	-3.002	3.184	2.048
N	-1.916	-5.321	-3.394	N	1.673	-4.777	-0.627	N	3.936	4.144	0.25	N	-3.022	2.846	4.317
N	-4.436	-4.543	-1.794	N	-0.226	-5.966	-0.156	N	4.073	3.185	-2.453	N	-3.605	0.639	6.079
N	-5.696	-0.06	3.315	N	4.552	-2.727	-1.448	N	4.543	1.524	4.142	N	-0.157	-3.2	3.773
N	5.806	-0.103	-3.092	N	-1.027	2.847	4.909	N	-3.876	2.354	-3.479	N	1.043	2.392	4.072
N	4.182	-2.266	-4.571	N	0.11	1.715	6.57	N	-3.979	1.492	-6.251	N	-0.143	4.051	-2.83
N	-4.302	2.499	4.33	N	-3.232	2.166	2.041	N	4.08	0.353	1.536	N	1.38	6.084	-0.164
N	-2.805	0.748	-1.165	N	3.268	-0.612	3.861	N	2.136	-2.003	-4.537	N	0.993	6.731	2.014
N	-0.709	-0.072	-3.039	N	1.856	-0.356	5.648	N	-0.487	-2.911	-5.554	N	0.394	4.58	3.84
N	-1.092	2.921	-0.205	N	5.29	-1.192	0.902	N	1.82	-3.504	-2.201	N	1.796	-1.148	4.357
N	0.601	3.38	4.018	N	-3.645	-2.167	-3.885	N	-4.227	-2.883	5.577	N	-3.975	-2.006	-4.389
N	0.997	2.103	-2.081	N	-2.986	-1.355	-5.945	N	-0.841	-4.432	-3.024	N	1.625	-4.252	1.667
N	3.874	3.559	-0.362	N	-4.253	-0.987	-0.45	N	-2.22	-4.867	0.329	N	0.305	-4.51	-1.705
N	4.309	4.72	1.561	N	1.471	-0.087	-4.896	N	-2.227	-5.533	2.541	N	-0.423	-4.54	-3.88
N	2.067	5.102	3.648	N	-0.546	0.138	-5.959	N	-3.608	-5.028	5.042	N	-2.851	-3.823	-5.257
N	0.253	-0.141	3.105	N	4.585	-0.3	-3.043	N	-3.422	0.666	5.032	N	-3.359	1.467	-3.331
H	-5.55	-2.672	-0.678	H	-0.414	10.647	0.538	H	2.4	1.863	-3.691	H	1.919	-0.923	-3.452
H	1.9	-3.009	-5.045	H	1.704	10.014	1.682	H	2.217	-0.709	0.417	H	4.333	-1.892	4.301
H	3.399	-2.894	1.01	H	2.638	7.749	1.391	H	-3.544	-0.476	-7.617	H	7.304	-1.987	1.206
H	2.646	-5.422	-2.37	H	-1.133	7.062	-2.31	H	-3.218	4.362	2.191	H	3.152	-2.735	0.408
H	-0.293	-2.674	-0.869	H	-2.104	2.864	-0.325	H	0.228	5.631	-0.06	H	6.707	-1.633	3.585
H	2.713	-0.416	-0.558	H	-4.342	4.474	-3.627	H	0.355	2.296	2.627	H	6.604	-2.604	-0.907
H	6.221	1.104	1.398	H	-5.695	1.071	-1.396	H	-2.459	1.487	0.803	H	6.462	-3.472	-2.833

H	2.84	-0.363	3.589	H	-6.019	2.686	-3.268	H	-6.562	1.668	-0.462	H	2.937	-2.8	-3.272
H	0.042	-4.278	1.64	H	2.293	3.586	-0.799	H	-5.42	1.839	3.678	H	6.562	-5.737	-3.645
H	-4.193	-4.365	2.301	H	7.346	1.047	-0.26	H	-2.108	3.554	6.242	H	2.65	-4.49	-6.395
H	-2.028	-0.687	1.825	H	5.59	4.945	0.314	H	1.978	3.202	7.5	H	5.972	-7.098	-5.622
H	5.086	0.695	3.577	H	3.327	1.572	-1.095	H	0.382	0.917	4.231	H	4.009	-6.454	-7.011
H	-2.049	-5.58	1.936	H	7.587	3.453	0.37	H	-7.199	1.839	1.942	H	0.493	0.841	-3.047
H	4.206	-4.586	-0.617	H	-2.402	5.481	-2.943	H	-0.307	4.128	7.859	H	0.771	-0.841	-6.997
H	-5.282	-2.254	2.314	H	0.873	5.056	-0.092	H	-2.174	5.913	0.55	H	-1.875	2.428	-6.046
H	0.527	-5.159	-3.409	H	-1.577	8.987	-0.88	H	3.294	1.49	6.285	H	-0.899	0.858	-7.729
H	6.003	1.095	-0.97	H	-3.613	-10	0.436	H	2.31	3.203	1.843	H	-3.832	-1.766	5.783
H	-1.484	-5.232	-4.302	H	-2.064	-9.96	-1.512	H	-3.051	1.223	-1.476	H	-2.366	2.125	-0.262
H	-4.948	-4.768	-0.954	H	-0.489	-8.084	-1.803	H	4.25	3.985	1.199	H	-3.886	5.725	1.537
H	-6.388	-0.384	2.656	H	-2.048	-6.507	3.042	H	3.126	3.55	-2.405	H	-3.105	5.392	-2.69
H	-7.944	-0.39	4.448	H	-2.512	-2.115	1.314	H	5.072	1.342	4.985	H	-3.939	6.724	-0.745
H	-9.085	1.034	6.099	H	-3.6	-3.183	5.337	H	6.491	3.264	4.137	H	-2.728	2.213	2.012
H	-5.749	3.72	6.024	H	-4.724	0.567	3.587	H	8.034	3.793	2.277	H	-2.45	2.027	4.128
H	-7.952	3.101	6.93	H	-4.713	-0.979	5.544	H	5.863	1.12	-0.282	H	-4.337	0.137	6.564
H	-2.334	-7.385	-4.816	H	1.286	-4.107	0.021	H	7.711	2.706	0.058	H	-3.103	5.214	5.58
H	-6.367	-6.113	-2.303	H	6.196	-3.109	-2.652	H	5.798	5.881	0.827	H	-3.757	1.333	8.568
H	-4.393	-8.705	-5.085	H	3.329	-6.301	-2.21	H	6.259	3.989	-3.72	H	-3.434	5.568	7.999
H	-6.43	-8.083	-3.777	H	2.873	-2.493	-0.266	H	7.749	6.497	-0.568	H	-3.749	3.616	9.513
H	6.091	0.864	-3.151	H	5.472	-5.447	-3.156	H	7.996	5.524	-2.844	H	0.196	-5.243	3.665
H	4.036	-3.265	-4.593	H	-2.457	-4.665	4.013	H	-3.222	1.602	-3.683	H	-3.475	-4.035	5.529
H	-3.744	2.857	5.092	H	-0.646	-5.067	0.061	H	-4.602	1.263	-7.014	H	-0.669	-1.19	3.959
H	8.147	0.105	-4.051	H	-3.566	-8.143	2.069	H	3.194	0.616	1.951	H	-1.958	-5.84	4.77
H	5.539	-3.306	-6.453	H	0.002	-0.378	10.615	H	-4.222	4.978	-3.216	H	1.109	2.414	3.065
H	9.173	-1.095	-5.939	H	1.584	-2.151	9.875	H	-4.164	3.466	-7.918	H	0.217	4.117	-4.874
H	7.863	-2.855	-7.134	H	2.444	-2.155	7.564	H	-4.513	6.619	-5.035	H	2.517	6.802	-2.443
H	-2.236	-1.249	-1.207	H	0.095	2.6	7.058	H	-4.486	5.861	-7.407	H	-0.374	4.11	-0.761
H	-6.424	-0.464	-0.679	H	-1.688	1.094	2.936	H	1.22	-0.348	-3.69	H	1.971	5.877	-4.676
H	-3.55	2.687	-1.11	H	-2.556	4.974	4.575	H	5.385	0.06	-4.668	H	2.035	6.853	-0.183
H	-2.632	0.262	-3.75	H	-4.755	3.348	1.272	H	3.186	-3.564	-5.409	H	1.634	7.483	1.8
H	-0.27	-2.585	-5.917	H	-4.326	5.223	2.86	H	0.426	-4.475	-6.57	H	0.193	4.342	2.873
H	1.236	-0.521	-2.467	H	2.641	0.033	3.403	H	-2.487	-2.194	-8.748	H	0.085	9.021	2.802
H	-1.944	1.65	1.2	H	7.013	-2.311	0.63	H	-1.454	-1.302	-4.668	H	-0.676	5.46	6.142
H	-1.75	5.809	2.25	H	5.424	-2.133	4.636	H	1.358	-1.6	-1.537	H	-0.93	9.586	4.984
H	-0.284	4.332	-1.498	H	3.584	-0.103	1.323	H	4.402	-3.692	0.656	H	-1.296	7.789	6.667
H	-2.437	-1.404	-5.58	H	7.174	-2.925	3.048	H	2.409	-5.412	-2.752	H	1.56	0.171	2.784
H	-0.61	6.223	0.075	H	-0.931	3.65	5.516	H	-0.876	-4.076	-8.668	H	1.225	1.82	6.743
H	-5.891	1.97	-0.715	H	1.143	-0.133	4.961	H	4.096	-5.597	-0.923	H	2.017	-2.358	6.027
H	0.229	3.943	4.772	H	-0.709	1.369	9.013	H	5.303	-2.257	-5.588	H	1.714	-0.459	7.625
H	1.514	2.369	-0.086	H	-4.155	0.798	-9.784	H	-4.847	-3.333	6.236	H	-4.809	-2.486	-4.698
H	4.367	3.713	-3	H	-2.013	2.06	-9.872	H	-0.579	-3.492	-1.197	H	0.316	-3.134	0.525
H	0.637	1.919	-4.117	H	-0.331	1.769	-8.092	H	-3.334	-6.786	-1.25	H	1.886	-6.704	-1.29
H	2.83	2.88	-4.774	H	-3.427	-2.188	-6.311	H	-1.199	-5.508	-4.76	H	2.943	-5.473	2.705
H	4.794	3.849	-0.669	H	-2.963	-0.364	-1.959	H	-2.817	-7.055	-3.672	H	3.106	-7.132	0.841
H	5.218	4.301	1.435	H	-5.433	-3.781	-2.809	H	-1.817	-4.014	0.692	H	-0.294	-3.701	-1.625
H	1.245	5.654	3.842	H	-5.613	-1.711	0.942	H	-2.263	-4.546	2.783	H	-0.687	-3.57	-3.729
H	6.154	6.433	1.875	H	-6.38	-3.572	-0.529	H	-4.42	-5.277	5.59	H	-3.772	-4.142	-5.525
H	2.617	7.001	5.246	H	0.925	-0.54	-4.178	H	-0.8	-7.785	2.603	H	1.139	-6.169	-5.336

H	6.379	8.193	3.58	H	6.517	0.241	-3.563	H	-3.005	-6.731	6.894	H	-2.896	-4.608	-7.724
H	4.565	8.506	5.269	H	3.474	0.777	-6.572	H	-0.52	-9.299	4.528	H	0.736	-6.881	-7.663
H	-0.524	-1.715	4.215	H	2.617	-0.812	-2.67	H	-1.632	-8.775	6.694	H	-1.286	-6.087	-8.878
H	-0.602	2.143	6.08	H	5.863	0.923	-5.877	H	-4.139	2.322	6.06	H	-4.73	3.006	-3.082
H	0.997	1.542	2.141	H	-4.024	-2.941	-4.413	H	-5.59	-1.502	7.337	H	-6.326	-0.796	-4.267
H	-1.171	-0.281	6.133	H	-0.956	0.102	-5.03	H	-2.788	-1.081	4.093	H	-2.125	-0.185	-3.62
H	-2.618	4.173	3.741	H	-4.59	-0.738	-7.895	H	-5.565	0.961	7.591	H	-6.691	1.576	-3.651
C	3.098	-1.489	-4.338	C	-1.296	5.358	-1.203	C	-3.015	0.593	-5.932	C	2.315	-2.098	-5.119
C	-4.037	-3.258	-1.946	C	-0.649	8.722	-0.381	C	4.347	1.941	-2.927	C	4.624	-2.018	3.266
C	-3.783	2.607	3.084	C	1.713	8.021	0.901	C	4.214	-0.839	0.878	C	5.942	-1.876	2.857
C	2.762	-3.244	0.209	C	-0.113	7.449	-0.563	C	-2.178	4.261	1.907	C	6.27	-2.073	1.527
C	3.2	-4.193	-0.7	C	0.002	9.653	0.416	C	-1.584	5.115	0.988	C	5.264	-2.398	0.607
C	2.334	-4.641	-1.681	C	1.185	9.296	1.056	C	-0.246	4.967	0.652	C	3.955	-2.491	1.084
C	1.043	-4.112	-1.753	C	1.079	7.089	0.077	C	0.491	3.945	1.269	C	4.789	-3.167	-1.716
C	0.691	-3.118	-0.845	C	-2.948	2.849	-0.997	C	-0.187	3.099	2.149	C	4.949	-4.346	-3.912
C	3.267	-0.159	0.333	C	-3.084	3.825	-1.99	C	-3.522	1.538	1.013	C	4.271	-5.874	-6.133
C	4.553	0.367	0.241	C	-4.2	3.745	-2.834	C	-4.45	1.548	-0.022	C	5.701	-5.47	-4.252
C	5.215	0.695	1.426	C	-5.14	2.75	-2.638	C	-5.808	1.653	0.314	C	3.827	-3.996	-4.675
C	4.597	0.454	2.641	C	-4.967	1.845	-1.602	C	-6.159	1.752	1.65	C	3.499	-4.773	-5.788
C	3.341	-0.13	2.658	C	2.907	5.441	-0.111	C	-5.174	1.749	2.627	C	5.371	-6.234	-5.361
C	-0.911	-3.78	1.755	C	6.505	1.729	-0.293	C	-1.125	3.105	6.163	C	0.209	0.796	-4.094
C	-2.083	-4.498	1.928	C	6.621	3.067	0.065	C	-0.112	3.432	7.05	C	0.765	-0.155	-4.952
C	-3.274	-3.817	2.113	C	5.515	3.901	0.04	C	1.157	2.907	6.852	C	0.354	-0.13	-6.297
C	-3.276	-2.42	2.098	C	4.276	3.372	-0.365	C	1.37	2.026	5.791	C	-0.576	0.815	-6.695
C	-2.071	-1.765	1.857	C	4.246	2.028	-0.738	C	0.278	1.663	5.008	C	-1.116	1.703	-5.775
C	-4.457	-0.599	3.221	C	-2.163	-4.777	1.972	C	3.208	1.682	4.232	C	-2.729	-0.078	5.338
C	5.132	-0.476	-1.978	C	-2.89	-8.137	1.218	C	-4.538	2.409	-2.293	C	-2.714	3.152	-0.314
C	-1.205	-4.879	-2.33	C	-1.167	-8.096	-0.96	C	2.6	4.178	0.016	C	-3.107	3.839	0.837
C	-6.201	0.847	4.286	C	-1.988	-7.087	1.068	C	5.335	1.858	2.992	C	-3.561	5.161	0.674
C	-7.475	2.467	6.191	C	-2.919	-9.178	0.301	C	7.055	2.465	0.888	C	-3.584	5.71	-0.597
C	-5.582	2.025	4.725	C	-2.052	-9.153	-0.788	C	5.155	1.245	1.746	C	-3.138	4.979	-1.688
C	-7.467	0.525	4.79	C	-1.114	-7.059	-0.027	C	6.363	2.787	3.169	C	-3.34	3.712	3.313
C	-8.108	1.318	5.726	C	-2.977	-1.894	2.262	C	7.23	3.081	2.125	C	-3.24	3.081	5.695
C	-6.232	2.808	5.686	C	-2.973	-2.838	3.294	C	6.014	1.57	0.693	C	-3.607	3.471	8.448
C	-3.167	-5.995	-3.414	C	-3.589	-2.487	4.502	C	4.957	4.569	-0.648	C	-3.233	4.367	6.239
C	-5.517	-7.506	-3.687	C	-4.213	-1.258	4.624	C	7.145	5.26	-2.226	C	-3.445	1.987	6.547
C	-3.229	-7.1	-4.271	C	-4.222	-0.391	3.543	C	5.913	5.463	-0.169	C	-3.61	2.19	7.916
C	-4.32	-5.624	-2.71	C	1.015	-6.022	-0.722	C	5.079	4.043	-1.935	C	-3.426	4.559	7.6
C	-5.475	-6.403	-2.852	C	5.281	-3.52	-2.243	C	6.174	4.4	-2.72	C	-0.501	-4.479	3.987
C	-4.383	-7.851	-4.417	C	4.861	-4.814	-2.522	C	7.008	5.802	-0.95	C	-1.701	-4.801	4.604
C	6.261	-0.906	-4.173	C	3.669	-5.295	-2.003	C	-4.057	3.29	-4.525	C	-2.546	-3.788	5.023
C	7.425	-2.303	-6.31	C	2.899	-4.452	-1.181	C	-4.362	5.148	-6.599	C	-2.186	-2.451	4.806
C	7.57	-0.641	-4.591	C	3.403	-3.177	-0.923	C	-4.207	4.651	-4.248	C	-0.98	-2.204	4.149
C	5.514	-1.867	-4.866	C	-0.512	1.632	5.37	C	-4.044	2.868	-5.859	C	0.752	3.609	4.728
C	6.122	-2.561	-5.919	C	-0.014	0.593	8.701	C	-4.185	3.803	-6.885	C	0.496	4.525	-3.911
C	8.154	-1.321	-5.647	C	1.749	-1.39	7.881	C	-4.376	5.57	-5.273	C	1.469	5.506	-3.79
C	-3.067	-0.568	-1.094	C	0.486	0.606	7.4	C	2.152	-0.736	-4.091	C	1.772	6.018	-2.54
C	-4.364	-1.052	-0.948	C	0.382	-0.388	9.599	C	3.303	0.049	-4.103	C	1.101	5.529	-1.411
C	-5.398	-0.126	-0.793	C	1.266	-1.378	9.183	C	4.468	-0.513	-4.649	C	0.158	4.517	-1.605
C	-5.111	1.227	-0.829	C	1.379	-0.388	6.98	C	4.422	-1.806	-5.146	C	0.68	5.864	1.022

C	-3.806	1.638	-1.042	C	-2.245	2.018	2.943	C	3.246	-2.537	-5.067	C	0.286	6.931	3.247
C	-1.719	-0.301	-3.896	C	-1.981	2.997	3.905	C	-0.305	-3.677	-6.64	C	-0.864	7.553	5.7
C	-1.598	-1.228	-4.918	C	-2.732	4.179	3.856	C	-1.028	-3.445	-7.8	C	-0.087	8.242	3.539
C	-0.397	-1.893	-5.09	C	-3.723	4.323	2.901	C	-1.927	-2.392	-7.838	C	0.057	5.911	4.181
C	0.663	-1.636	-4.217	C	-3.967	3.289	2.012	C	-2.097	-1.58	-6.71	C	-0.522	6.239	5.409
C	0.449	-0.739	-3.174	C	3.075	-0.82	5.242	C	-1.356	-1.89	-5.567	C	-0.657	8.56	4.763
C	-1.662	2.671	0.985	C	6.284	-1.967	1.353	C	1.966	-2.479	-1.347	C	1.573	0.081	3.866
C	-1.945	3.688	1.893	C	6.354	-2.309	2.698	C	2.871	-2.499	-0.295	C	1.335	1.188	4.683
C	-1.546	4.987	1.57	C	5.383	-1.875	3.586	C	3.668	-3.643	-0.137	C	1.39	0.986	6.075
C	-0.927	5.227	0.356	C	4.331	-1.075	3.103	C	3.5	-4.697	-1.021	C	1.659	-0.284	6.557
C	-0.734	4.176	-0.526	C	4.347	-0.748	1.747	C	2.564	-4.606	-2.045	C	1.844	-1.345	5.682
C	1.908	3.758	3.593	C	-3.042	-1.134	-4.609	C	-3.343	-3.707	4.877	C	-2.749	-2.62	-4.644
C	1.837	2.498	-1.109	C	-3.654	-0.188	-7.946	C	-1.115	-4.279	-1.717	C	0.931	-4.029	0.54
C	3.054	3.11	-1.395	C	-1.265	1.225	-8.048	C	-2.004	-5.097	-1.027	C	1.006	-4.871	-0.571
C	3.426	3.238	-2.735	C	-2.698	-0.37	-6.949	C	-2.64	-6.121	-1.746	C	1.811	-6.02	-0.457
C	2.569	2.794	-3.726	C	-3.412	0.676	-9.004	C	-2.345	-6.27	-3.092	C	2.488	-6.248	0.729
C	1.346	2.25	-3.37	C	-2.213	1.381	-9.051	C	-1.445	-5.413	-3.71	C	2.396	-5.344	1.778
C	3.336	4.344	0.698	C	-1.488	0.335	-6.996	C	-2.295	-5.949	1.245	C	0.271	-5.221	-2.924
C	4.266	5.708	2.583	C	-3.706	-1.092	-1.675	C	-2.079	-6.413	3.639	C	-0.643	-4.989	-5.203
C	4.493	7.728	4.518	C	-4.124	-2.072	-2.581	C	-1.749	-8.117	5.841	C	-1.103	-5.783	-7.853
C	5.38	6.554	2.628	C	-5.087	-2.993	-2.146	C	-1.278	-7.554	3.546	C	0.263	-5.82	-5.865
C	3.265	5.861	3.551	C	-5.62	-2.881	-0.874	C	-2.701	-6.124	4.857	C	-1.793	-4.566	-5.882
C	3.395	6.885	4.496	C	-5.197	-1.852	-0.048	C	-2.522	-6.971	5.951	C	-2.006	-4.952	-7.205
C	5.506	7.552	3.579	C	0.81	0.215	-6.105	C	-1.128	-8.407	4.629	C	0.028	-6.221	-7.173
C	-0.332	-0.651	4.203	C	5.492	0.172	-3.907	C	-4.17	1.241	5.986	C	-4.607	1.96	-3.334
C	-0.683	0.157	5.272	C	5.112	0.557	-5.186	C	-4.963	0.48	6.829	C	-5.691	1.156	-3.653
C	-0.379	1.507	5.229	C	3.785	0.483	-5.579	C	-4.973	-0.895	6.681	C	-5.482	-0.169	-3.994
C	0.254	2.033	4.1	C	2.833	0	-4.662	C	-4.183	-1.501	5.692	C	-4.179	-0.684	-3.999
C	0.52	1.174	3.038	C	3.297	-0.396	-3.408	C	-3.413	-0.668	4.871	C	-3.14	0.175	-3.634

Table S4-2. Cartesian coordinates cage complexes with *n*-octyl glycosides computed with DFT at the ωB97X-D, 6-31G* level of theory. See also Figure S4-3.

n-Oct-α-Man				*n*-Oct-β-Glc				*n*-Oct-α-Glc				*n*-Oct-β-Gal			
	x	y	z		x	y	z		x	y	z		x	y	z
Pd	2.586	-2.216	3.735	Pd	1.971	-3.633	2.381	Pd	1.51	-1.854	3.864	Pd	2.312	-2.21	4.214
Pd	-2.447	3.739	-3.002	Pd	-1.769	2.068	-3.469	Pd	-1.71	3.399	-2.995	Pd	-1.087	3.503	-2.946
O	2.641	-0.054	-6.148	O	2.518	-2.567	-6.714	O	2.983	-1.102	-5.784	O	4.144	-0.391	-5.319
O	3.982	-1.361	-1.206	O	4.088	-3.753	-2.053	O	3.894	-1.412	-0.805	O	4.29	-1.294	-0.294
O	-1.398	0.86	5.007	O	-2.426	-1.041	3.414	O	-2.205	1.138	5.221	O	-1.917	0.461	4.735
O	-4.863	4.141	3.471	O	-5.286	3.155	2.607	O	-4.173	5.411	3.323	O	-4.935	4.22	2.98
O	0.259	3.597	6.025	O	2.37	2.873	5.22	O	0.076	4.707	5.908	O	-0.009	3.842	6.881
O	-0.11	5.045	0.992	O	1.107	3.954	0.517	O	-0.087	5.635	0.966	O	-0.245	4.857	1.906
O	-2.941	-1.477	-2.568	O	-1.733	-2.567	-4.795	O	-2.721	-1.76	-2.893	O	-2.612	-1.963	-2.484
O	-0.314	-6.003	-1.481	O	1.384	-6.436	-4.059	O	-0.711	-6.149	-1.301	O	0.335	-5.794	-1.423
O	-1.202	0.151	-0.578	O	0.25	0.804	0.161	O	-1.002	0.142	-0.963	O	-0.206	-0.109	-0.355
O	-2.385	-2.109	0.281	O	-2.631	-1.382	0.656	O	2.527	1.079	-0.921	O	-2.215	-1.784	0.439
O	0.841	-0.431	-1.549	O	-1.147	-2.79	-1.253	O	1.496	2.306	1.479	O	1.365	-0.19	-1.992
O	1.353	2.591	1.544	O	1.638	-2.01	-1.638	O	-0.998	1.136	2.498	O	3.175	1.272	-0.299
O	-0.265	2.836	-0.771	O	-1.598	1.193	1.407	O	-2.83	-1.66	-0.067	O	1.999	2.266	2.126
O	0.098	0.309	2.811	O	2.764	1.661	0.203	O	0.941	-1.15	-1.134	O	-0.654	2.104	1.451
N	1.75	0.959	-4.299	N	1.738	-1.619	-4.773	N	2.389	0.425	-4.185	N	3.502	1.091	-3.688
N	4.404	-1.689	2.898	N	3.881	-3.056	1.87	N	3.458	-1.344	3.327	N	4.187	-1.435	3.786
N	6.059	-0.971	-0.322	N	5.888	-2.634	-1.175	N	5.771	-1.078	0.458	N	6.274	-0.938	0.802
N	5.799	-1.319	-2.595	N	5.926	-3.406	-3.358	N	5.946	-1.84	-1.715	N	6.219	-2.1	-1.198
N	3.396	-0.652	-4.063	N	3.394	-3.213	-4.691	N	3.901	-1.236	-3.675	N	4.45	-0.926	-3.105
N	-1.387	2.777	-4.484	N	-0.814	0.958	-4.924	N	-0.716	2.276	-4.407	N	0.338	2.777	-4.231
N	-2.356	-1.125	5.704	N	-3.245	-3.1	3.995	N	-3.549	-0.72	5.351	N	-2.973	-1.569	5.006
N	-3.376	4.491	-1.317	N	-2.782	3.022	-1.956	N	-2.576	4.474	-1.467	N	-2.476	4.171	-1.603
N	-2.964	3.769	2.236	N	-4.428	1.756	1.011	N	-3.001	3.862	2.097	N	-3.308	3.148	1.764
N	-3.067	3.125	4.463	N	-4.335	1.145	3.228	N	-3.37	3.525	4.351	N	-3.577	2.674	3.995
N	-3.228	0.864	6.39	N	-4.135	-1.188	4.926	N	-4.356	1.359	5.951	N	-3.997	0.368	5.695
N	0.776	-2.742	4.63	N	0.065	-4.316	2.896	N	-0.373	-2.367	4.531	N	0.426	-2.922	4.648
N	1.769	2.778	4.494	N	2.365	1.521	3.37	N	0.606	3.181	4.264	N	0.972	2.658	5.176
N	-0.93	5.179	-2.909	N	-0.139	3.347	-3.409	N	-0.057	4.64	-2.864	N	0.322	4.831	-2.2
N	0.97	6.703	-0.169	N	2.161	5.129	-1.138	N	1.675	6.505	-0.216	N	1.38	6.235	1.03
N	0.847	6.763	2.147	N	2.34	5.817	1.044	N	1.135	7.318	1.892	N	0.288	6.813	2.967
N	0.612	4.691	4.021	N	2.173	3.798	3.117	N	0.08	5.348	3.703	N	-0.148	4.601	4.715
N	2.752	-0.656	5.056	N	1.9	-2.062	3.711	N	1.437	-0.232	5.125	N	2.04	-0.767	5.633
N	-5.167	-0.995	-2.372	N	-4.002	-2.705	-4.504	N	-4.889	-1.093	-3.211	N	-3.112	-1.271	-4.628
N	2.232	-3.657	2.32	N	2.088	-5.06	0.9	N	1.358	-3.407	2.536	N	2.372	-3.495	2.592
N	-0.591	-4.435	0.173	N	0.717	-4.928	-2.446	N	-1.086	-4.303	0.005	N	0.138	-3.822	-0.256
N	-2.138	-4.605	-1.519	N	-0.188	-4.832	-4.552	N	-2.167	-4.525	-2.02	N	-1.473	-4.418	-1.775
N	-4.428	-3.203	-2.338	N	-2.832	-4.403	-5.574	N	-4.293	-3.301	-3.511	N	-2.758	-3.47	-4.235
N	-4.034	2.427	-3.143	N	-3.411	0.826	-3.692	N	-3.469	2.315	-3.158	N	-2.509	2.244	-3.742
H	1.744	0.932	-3.287	H	1.95	-1.474	-3.791	H	2.61	0.787	-3.262	H	3.412	1.187	-2.681
H	5.244	-1.685	4.796	H	4.5	-3.076	3.852	H	3.881	-1.021	5.332	H	4.691	-1.2	5.785
H	7.843	-0.637	1.555	H	7.532	-2.388	0.894	H	7.096	-0.354	2.581	H	7.728	-0.206	2.912
H	3.706	-1.669	0.932	H	3.398	-2.976	-0.153	H	3.194	-1.663	1.287	H	3.831	-1.603	1.752
H	7.499	-1.005	3.982	H	6.88	-2.578	3.279	H	6.246	-0.368	4.912	H	6.986	-0.359	5.275

H	7.039	-0.866	-0.546	H	6.857	-2.389	-1.332	H	6.782	-1.061	0.444	H	7.276	-0.875	0.676
H	6.803	-1.201	-2.625	H	6.878	-3.066	-3.379	H	6.94	-1.833	-1.526	H	7.201	-2.278	-1.032
H	3.278	-0.53	-3.063	H	3.29	-3.179	-3.682	H	3.719	-0.97	-2.713	H	4.129	-0.69	-2.174
H	6.899	-3.524	-3.394	H	7.468	-5.262	-4.33	H	7.058	-4.126	-2.08	H	6.639	-4.633	-1.579
H	2.971	-2.318	-6.128	H	3.23	-4.813	-6.833	H	3.832	-3.073	-5.664	H	3.779	-2.637	-5.085
H	6.284	-4.921	-5.342	H	7.076	-6.655	-6.337	H	6.794	-5.683	-3.983	H	5.847	-6.033	-3.464
H	4.3	-4.298	-6.712	H	4.939	-6.425	-7.591	H	5.169	-5.128	-5.791	H	4.407	-5.015	-5.22
H	0.179	2.697	-3.145	H	0.679	0.463	-3.587	H	0.937	2.309	-3.166	H	1.478	2.283	-2.583
H	0.721	0.575	-6.84	H	0.602	-1.753	-7.266	H	1.211	-0.242	-6.602	H	3.163	1.32	-6.408
H	-2.857	2.812	-5.947	H	-2.203	1.399	-6.399	H	-2.265	2.178	-5.783	H	-0.703	3.185	-5.98
H	-1.495	1.459	-7.551	H	-1.259	-0.272	-7.999	H	-1.022	0.617	-7.291	H	1.116	2.3	-7.447
H	-3.188	-1.508	6.132	H	-4.012	-3.584	4.443	H	-4.43	-1.068	5.706	H	-3.858	-2.003	5.233
H	-2.379	3.047	-0.21	H	-3.073	1.173	-1.09	H	-2.652	2.778	-0.296	H	-1.924	2.614	-0.358
H	-4.732	5.871	2.013	H	-4.971	4.47	0.871	H	-3.394	6.529	1.68	H	-4.906	5.315	1.152
H	-4.443	5.983	-2.282	H	-2.596	4.95	-2.704	H	-2.608	6.247	-2.542	H	-3.099	5.795	-2.736
H	-5.404	6.89	-0.166	H	-4.024	5.941	-0.907	H	-3.234	7.618	-0.548	H	-4.744	6.549	-1.011
H	-1.96	3.86	2.344	H	-3.985	0.853	0.891	H	-2.754	2.881	2.069	H	-2.465	2.583	1.838
H	-2.227	2.578	4.315	H	-3.671	0.455	2.893	H	-2.761	2.716	4.274	H	-2.812	2.019	3.882
H	-3.793	0.258	6.969	H	-4.693	-1.831	5.473	H	-5.198	0.865	6.213	H	-4.692	-0.265	6.067
H	-3.961	5.354	5.538	H	-4.618	3.419	4.659	H	-3.629	5.902	5.568	H	-4.317	4.951	5.178
H	-3.678	1.657	8.782	H	-3.98	-0.619	7.431	H	-5.064	2.077	8.348	H	-4.663	0.994	8.114
H	-4.576	5.749	7.894	H	-4.557	3.615	7.107	H	-4.462	6.293	7.856	H	-5.11	5.212	7.497
H	-4.389	3.89	9.546	H	-4.23	1.588	8.521	H	-5.187	4.373	9.263	H	-5.265	3.225	8.992
H	1.2	-4.769	4.497	H	0.7	-6.279	2.689	H	0.209	-4.33	4.878	H	1.064	-4.892	4.757
H	-2.747	-3.723	5.809	H	-3.377	-5.716	3.891	H	-3.76	-3.253	6.102	H	-3.149	-4.199	5.21
H	0.237	-0.762	4.896	H	-0.71	-2.409	3.179	H	-1.1	-0.445	4.21	H	-0.356	-0.991	4.586
H	-1.056	-5.464	5.301	H	-1.51	-7.244	3.316	H	-1.99	-4.976	5.866	H	-1.244	-5.779	5.129
H	1.809	2.662	3.481	H	2.574	1.487	2.373	H	1.085	3.141	3.367	H	1.296	2.707	4.215
H	-0.556	5.223	-4.95	H	-0.207	3.444	-5.481	H	0.102	4.842	-4.924	H	0.978	5.33	-4.104
H	2.275	7.28	-2.454	H	2.87	5.724	-3.567	H	2.662	7.339	-2.546	H	2.872	7.363	-0.836
H	-1.204	5.335	-0.871	H	0.035	3.355	-1.334	H	-0.09	4.546	-0.789	H	-0.234	4.448	-0.235
H	1.479	6.64	-4.726	H	1.745	4.982	-5.648	H	1.92	6.549	-4.776	H	2.672	6.961	-3.275
H	1.483	7.571	-0.094	H	2.823	5.869	-1.331	H	2.387	7.223	-0.226	H	1.985	7.013	1.258
H	1.409	7.602	2.057	H	2.884	6.568	0.64	H	1.866	7.999	1.737	H	0.773	7.693	2.849
H	1.347	4.818	3.337	H	1.857	3.554	2.184	H	0.053	5.014	2.743	H	-0.096	4.305	3.747
H	-0.783	8.765	2.565	H	1.68	8.157	1.851	H	0.28	9.724	2.374	H	-1.266	8.835	3.435
H	-1.184	5.229	6.004	H	1.75	4.898	5.557	H	-1.448	6.423	5.62	H	-1.877	5.175	6.706
H	-2.355	9.066	4.464	H	1.258	8.925	4.163	H	-1.156	10.478	4.241	H	-2.817	9.137	5.339
H	-2.531	7.274	6.182	H	1.318	7.27	6.024	H	-2.031	8.809	5.867	H	-3.104	7.296	6.989
H	2.495	0.553	3.4	H	2.603	-0.796	2.232	H	1.637	0.961	3.451	H	2.126	0.613	4.091
H	1.848	2.389	7.227	H	1.632	1.036	5.986	H	0.153	2.881	6.988	H	0.527	2.014	7.819
H	2.967	-1.75	6.805	H	1.265	-3.207	5.321	H	1.229	-1.291	6.898	H	1.934	-2.03	7.276
H	2.492	0.222	8.27	H	1.106	-1.233	6.849	H	0.507	0.727	8.187	H	1.03	-0.234	8.77
H	-6.089	-1.409	-2.411	H	-4.832	-3.232	-4.743	H	-5.818	-1.409	-3.456	H	-3.578	-1.623	-5.453
H	0.424	-2.753	1.896	H	0.462	-4.161	-0.001	H	-0.359	-2.541	1.777	H	0.436	-2.91	2.157
H	1.226	-6.483	-0.088	H	2.759	-6.811	-2.465	H	0.634	-6.438	0.283	H	2.436	-5.197	-0.871
H	4.017	-4.661	2.645	H	3.714	-6.088	1.676	H	3.052	-4.396	3.21	H	4.268	-4.267	2.919
H	3.415	-6.528	1.091	H	4.121	-7.342	-0.447	H	2.617	-6.406	1.781	H	4.35	-5.417	0.701
H	-1.108	-3.581	0.385	H	-0.125	-4.438	-2.15	H	-1.634	-3.449	0.106	H	-0.575	-3.187	0.107
H	-2.67	-3.942	-0.968	H	-0.563	-3.933	-4.264	H	-2.467	-3.578	-1.84	H	-1.802	-3.459	-1.704

H	-5.402	-3.414	-2.155	H	-3.736	-4.827	-5.734	H	-5.273	-3.436	-3.724	H	-2.744	-3.48	-5.247
H	-1.284	-6.44	-3.34	H	1.436	-6.003	-6.337	H	-0.995	-6.561	-3.378	H	-1.9	-7.004	-1.355
H	-5.229	-3.816	-4.755	H	-3.094	-5.062	-8.076	H	-4.526	-4.214	-5.912	H	-4.214	-5.258	-5.351
H	-2.279	-6.821	-5.554	H	0.864	-6.6	-8.664	H	-1.525	-7.262	-5.669	H	-3.269	-8.519	-2.736
H	-4.267	-5.516	-6.289	H	-1.417	-6.135	-9.544	H	-3.305	-6.093	-6.966	H	-4.439	-7.641	-4.755
H	-5.128	3.988	-3.965	H	-4.742	2.419	-3.735	H	-4.469	4.116	-3.412	H	-3.595	3.916	-4.314
H	-7.234	0.318	-3.293	H	-6.346	-1.451	-4.672	H	-6.87	0.571	-3.506	H	-5.074	0.123	-5.648
H	-3.058	0.781	-2.323	H	-2.209	-0.867	-3.663	H	-2.597	0.44	-2.913	H	-1.521	0.488	-3.23
H	-7.246	2.675	-4.067	H	-6.701	0.975	-4.297	H	-6.705	3.04	-3.641	H	-5.303	2.601	-5.563
H	1.694	0.709	0.918	H	-0.081	-2.326	0.461	H	1.311	0.245	1.336	H	1.292	1.992	-0.446
H	-0.032	-1.072	0.591	H	1.605	-0.678	0.622	H	-0.553	-0.91	0.734	H	2.07	0.208	1.899
H	-2.921	-0.374	1.295	H	0.149	-0.628	-2.049	H	-1.013	2.094	0.667	H	1.618	-1.04	-0.156
H	-1.894	-1.533	2.17	H	-1.871	-0.242	-0.831	H	0.813	2.131	-0.996	H	0.085	-1.13	1.4
H	-2.389	-1.708	-0.611	H	-0.296	-0.331	1.788	H	2.823	0.156	-0.771	H	-2.566	0.238	0.789
H	0.653	3.267	1.577	H	-2.502	-1.394	1.628	H	2.379	2.391	1.087	H	-2.193	-0.761	2.205
H	-1.128	1.558	1.687	H	-2.017	-2.957	-0.855	H	-0.661	1.911	2.979	H	-2.409	-1.558	-0.486
H	1.574	1.943	-1.105	H	1.263	-2.8	-2.062	H	-2.806	-0.646	1.698	H	3.62	0.415	-0.136
H	0.074	3.615	-0.295	H	3.253	0.088	-0.992	H	-3.29	0.335	0.297	H	2.838	2.515	1.709
H	-0.553	0.51	3.511	H	2.091	1.225	-1.711	H	-2.671	-1.47	-1.012	H	-0.075	0.742	2.889
H	0.309	-1.087	-3.436	H	2.059	2.336	0.235	H	0.319	0.287	-2.426	H	-0.15	2.904	1.693
H	-0.666	-1.755	-2.126	H	-1.445	1.282	3.458	H	0.758	-1.611	-3.138	H	0.218	-1.906	-2.031
H	2.317	-2.34	-2.328	H	-0.001	1.822	2.568	H	-0.421	-2.395	-2.085	H	1.967	-2.151	-2.252
H	1.128	-3.194	-1.38	H	-2.62	3.358	2.397	H	2.581	-2.963	-1.972	H	1.75	-1.062	-4.542
H	1.21	-3.088	-4.441	H	-1.011	3.884	1.91	H	1.321	-3.899	-1.197	H	0.03	-0.847	-4.24
H	0.036	-4.011	-3.516	H	-1.979	3.287	4.761	H	1.757	-3.583	-4.208	H	-0.086	-3.397	-3.822
H	3.069	-4.423	-3.401	H	-0.327	3.745	4.384	H	0.37	-4.416	-3.526	H	1.551	-3.503	-4.437
H	1.868	-5.394	-2.547	H	-2.745	5.483	3.683	H	3.276	-5.2	-2.975	H	0.767	-2.568	-6.64
H	2.064	-5.13	-5.588	H	-1.048	5.936	3.548	H	1.841	-6.07	-2.428	H	-0.833	-2.343	-5.964
H	0.9	-6.129	-4.73	H	-2.554	5.32	6.144	H	2.581	-5.603	-5.363	H	-0.705	-4.95	-5.425
H	3.94	-6.461	-4.539	H	-0.829	5.633	6.065	H	1.206	-6.534	-4.797	H	0.582	-4.971	-6.608
H	2.747	-7.505	-3.779	H	-3.03	7.59	5.215	H	4.139	-7.205	-4.208	H	-0.986	-4.002	-8.326
H	3.083	-7.106	-6.798	H	-1.308	7.894	5.045	H	2.761	-8.159	-3.682	H	-2.266	-3.947	-7.136
H	1.869	-8.134	-6.054	H	-2.747	7.386	7.702	H	3.501	-7.608	-6.603	H	-2.061	-6.458	-6.839
H	4.91	-8.522	-5.776	H	-1.024	7.675	7.531	H	2.128	-8.567	-6.076	H	-0.876	-6.481	-8.134
H	3.838	-9.487	-6.799	H	-3.232	9.672	6.749	H	5.075	-9.246	-5.499	H	-3.135	-7.064	-9.022
H	3.68	-9.561	-5.04	H	-1.495	9.964	6.578	H	4.047	-10.046	-6.695	H	-2.597	-5.537	-9.73
H	-0.537	0.911	-2.266	H	-2.208	9.778	8.186	H	3.688	-10.212	-4.972	H	-3.792	-5.534	-8.425
C	2.619	0.055	-4.94	C	2.566	-2.494	-5.504	C	3.104	-0.696	-4.647	C	4.057	-0.116	-4.141
C	5.433	-1.518	3.743	C	4.819	-2.946	2.825	C	4.285	-1.003	4.328	C	5.039	-1.095	4.765
C	6.682	-1.136	3.282	C	6.137	-2.673	2.496	C	5.6	-0.639	4.086	C	6.314	-0.63	4.469
C	6.866	-0.934	1.928	C	6.494	-2.562	1.162	C	6.066	-0.635	2.786	C	6.723	-0.543	3.149
C	5.8	-1.126	1.038	C	5.518	-2.7	0.168	C	5.216	-1.011	1.738	C	5.837	-0.915	2.13
C	4.561	-1.503	1.573	C	4.196	-2.905	0.572	C	3.895	-1.357	2.052	C	4.557	-1.325	2.5
C	5.189	-1.236	-1.378	C	5.226	-3.313	-2.203	C	5.126	-1.453	-0.71	C	5.522	-1.45	-0.253
C	5.301	-2.122	-3.679	C	5.579	-4.252	-4.462	C	5.609	-2.721	-2.805	C	5.649	-2.858	-2.274
C	4.586	-3.696	-5.856	C	5.121	-5.825	-6.707	C	5.296	-4.462	-4.943	C	4.75	-4.418	-4.382
C	6.037	-3.263	-4.002	C	6.538	-5.171	-4.885	C	6.348	-3.901	-2.871	C	5.998	-4.206	-2.346
C	4.179	-1.773	-4.439	C	4.367	-4.131	-5.154	C	4.675	-2.412	-3.806	C	4.832	-2.279	-3.252
C	3.829	-2.581	-5.525	C	4.15	-4.93	-6.277	C	4.533	-3.304	-4.876	C	4.384	-3.081	-4.305
C	5.694	-4.047	-5.092	C	6.318	-5.953	-6.009	C	6.202	-4.775	-3.937	C	5.556	-4.99	-3.402

C	-0.17	2.348	-4.11	C	0.242	0.219	-4.548	C	0.514	1.868	-4.061	C	1.433	2.261	-3.663
C	0.603	1.485	-4.885	C	0.76	-0.814	-5.328	C	1.229	0.904	-4.772	C	2.466	1.689	-4.399
C	0.128	1.187	-6.175	C	0.211	-0.987	-6.612	C	0.672	0.463	-5.985	C	2.369	1.726	-5.796
C	-1.107	1.685	-6.564	C	-0.826	-0.162	-7.012	C	-0.575	0.943	-6.359	C	1.223	2.267	-6.369
C	-1.868	2.451	-5.692	C	-1.35	0.783	-6.14	C	-1.27	1.824	-5.542	C	0.211	2.773	-5.569
C	-2.265	0.265	5.66	C	-3.222	-1.714	4.089	C	-3.295	0.645	5.504	C	-2.895	-0.175	5.133
C	-3.048	3.899	-0.153	C	-3.28	2.229	-1.001	C	-2.74	3.859	-0.284	C	-2.564	3.484	-0.458
C	-3.477	4.389	1.076	C	-4.047	2.695	0.063	C	-2.994	4.556	0.898	C	-3.392	3.883	0.596
C	-4.353	5.481	1.078	C	-4.347	4.065	0.086	C	-3.177	5.948	0.796	C	-4.222	4.997	0.378
C	-4.73	6.042	-0.136	C	-3.815	4.878	-0.908	C	-3.082	6.55	-0.447	C	-4.127	5.676	-0.828
C	-4.209	5.541	-1.32	C	-3.026	4.343	-1.916	C	-2.751	5.799	-1.565	C	-3.229	5.262	-1.801
C	-3.728	3.716	3.419	C	-4.74	2.106	2.334	C	-3.575	4.357	3.291	C	-4.023	3.42	2.935
C	-3.472	3.292	5.809	C	-4.342	1.292	4.635	C	-3.864	3.776	5.656	C	-4.051	2.831	5.316
C	-4.141	3.73	8.503	C	-4.256	1.51	7.44	C	-4.816	4.21	8.257	C	-4.926	3.12	7.967
C	-3.891	4.548	6.257	C	-4.468	2.536	5.264	C	-3.924	5.066	6.186	C	-4.387	4.087	5.825
C	-3.43	2.241	6.728	C	-4.169	0.157	5.439	C	-4.29	2.702	6.446	C	-4.172	1.715	6.152
C	-3.739	2.477	8.071	C	-4.117	0.275	6.828	C	-4.749	2.923	7.743	C	-4.589	1.869	7.474
C	-4.237	4.768	7.58	C	-4.437	2.639	6.648	C	-4.407	5.281	7.47	C	-4.834	4.228	7.131
C	0.447	-4.036	4.758	C	-0.138	-5.641	2.939	C	-0.599	-3.615	4.972	C	0.206	-4.234	4.804
C	-0.814	-4.414	5.196	C	-1.372	-6.169	3.287	C	-1.823	-3.963	5.519	C	-1.081	-4.715	5.004
C	-1.751	-3.437	5.483	C	-2.409	-5.312	3.607	C	-2.809	-2.998	5.644	C	-2.141	-3.827	5.049
C	-1.401	-2.085	5.37	C	-2.198	-3.927	3.583	C	-2.564	-1.695	5.197	C	-1.905	-2.452	4.906
C	-0.104	-1.781	4.959	C	-0.933	-3.466	3.205	C	-1.327	-1.425	4.606	C	-0.589	-2.04	4.694
C	0.823	3.697	4.956	C	2.314	2.775	4.011	C	0.235	4.467	4.731	C	0.234	3.733	5.698
C	-0.212	5.559	-3.98	C	0.31	3.788	-4.594	C	0.484	5.18	-3.969	C	1.107	5.516	-3.045
C	0.927	6.34	-3.843	C	1.398	4.641	-4.679	C	1.49	6.131	-3.874	C	2.046	6.421	-2.575
C	1.364	6.703	-2.576	C	2.019	5.051	-3.516	C	1.904	6.564	-2.625	C	2.154	6.639	-1.213
C	0.602	6.321	-1.471	C	1.539	4.612	-2.274	C	1.32	6.019	-1.475	C	1.316	5.944	-0.333
C	-0.563	5.599	-1.693	C	0.446	3.736	-2.259	C	0.368	5.012	-1.646	C	0.422	5.019	-0.874
C	0.528	6.109	0.991	C	1.815	4.916	0.187	C	0.836	6.446	0.9	C	0.41	5.903	1.978
C	-0.109	6.841	3.228	C	1.961	6.103	2.401	C	0.234	7.657	2.957	C	-0.691	6.848	4.013
C	-1.864	7.154	5.335	C	1.491	6.952	5.002	C	-1.401	8.491	5.043	C	-2.436	7.172	6.143
C	-0.886	7.991	3.32	C	1.691	7.444	2.671	C	-0.104	9.003	3.092	C	-1.406	8.035	4.157
C	-0.205	5.826	4.187	C	1.966	5.159	3.44	C	-0.259	6.713	3.865	C	-0.865	5.799	4.923
C	-1.105	5.992	5.244	C	1.731	5.609	4.744	C	-1.088	7.147	4.902	C	-1.749	5.975	5.99
C	-1.766	8.159	4.38	C	1.457	7.878	3.966	C	-0.912	9.427	4.137	C	-2.276	8.205	5.224
C	2.484	0.522	4.482	C	2.221	-0.845	3.244	C	1.341	0.943	4.492	C	1.83	0.455	5.121
C	2.111	1.643	5.215	C	2.108	0.321	4.002	C	0.84	2.089	5.102	C	1.242	1.486	5.851
C	2.12	1.541	6.613	C	1.697	0.171	5.341	C	0.532	2.013	6.469	C	0.955	1.232	7.206
C	2.466	0.326	7.191	C	1.408	-1.098	5.817	C	0.722	0.806	7.128	C	1.233	-0.022	7.726
C	2.754	-0.771	6.394	C	1.501	-2.204	4.984	C	1.141	-0.316	6.432	C	1.754	-1.023	6.917
C	-4.092	-1.874	-2.445	C	-2.777	-3.194	-4.966	C	-3.883	-2.047	-3.201	C	-2.814	-2.238	-3.668
C	1.057	-3.606	1.673	C	1.291	-4.838	-0.153	C	0.292	-3.405	1.723	C	1.307	-3.429	1.778
C	0.641	-4.594	0.781	C	1.512	-5.405	-1.404	C	-0.004	-4.456	0.855	C	1.271	-4.009	0.513
C	1.51	-5.683	0.581	C	2.558	-6.333	-1.516	C	0.843	-5.58	0.905	C	2.41	-4.724	0.101
C	2.73	-5.702	1.24	C	3.318	-6.616	-0.391	C	1.948	-5.555	1.742	C	3.476	-4.841	0.981
C	3.077	-4.676	2.106	C	3.093	-5.944	0.801	C	2.199	-4.452	2.545	C	3.442	-4.214	2.22
C	-0.974	-5.104	-0.999	C	0.691	-5.492	-3.748	C	-1.287	-5.091	-1.14	C	-0.296	-4.782	-1.197
C	-2.672	-4.897	-2.786	C	-0.492	-5.194	-5.886	C	-2.451	-4.984	-3.325	C	-2.252	-5.28	-2.578
C	-3.819	-5.341	-5.317	C	-1.155	-5.872	-8.525	C	-3.062	-5.785	-5.955	C	-3.83	-6.987	-4.141

C	-2.136	-5.857	-3.654	C	0.452	-5.783	-6.728	C	-1.76	-6.039	-3.934	C	-2.409	-6.625	-2.232
C	-3.792	-4.169	-3.207	C	-1.778	-4.943	-6.384	C	-3.454	-4.336	-4.06	C	-2.912	-4.789	-3.706
C	-4.355	-4.391	-4.459	C	-2.096	-5.271	-7.701	C	-3.745	-4.734	-5.363	C	-3.704	-5.648	-4.474
C	-2.708	-6.067	-4.902	C	0.12	-6.128	-8.031	C	-2.069	-6.433	-5.229	C	-3.176	-7.475	-3.013
C	-5.168	2.959	-3.626	C	-4.637	1.348	-3.859	C	-4.58	3.04	-3.358	C	-3.538	2.836	-4.37
C	-6.34	2.225	-3.679	C	-5.717	0.538	-4.176	C	-5.82	2.435	-3.481	C	-4.481	2.1	-5.065
C	-6.325	0.912	-3.245	C	-5.514	-0.818	-4.378	C	-5.904	1.058	-3.405	C	-4.347	0.724	-5.108
C	-5.138	0.347	-2.765	C	-4.23	-1.355	-4.228	C	-4.744	0.295	-3.213	C	-3.276	0.105	-4.454
C	-3.996	1.156	-2.71	C	-3.207	-0.494	-3.826	C	-3.524	0.971	-3.079	C	-2.37	0.907	-3.759
C	-0.332	0.806	1.569	C	-0.77	0.213	0.946	C	0.351	0.076	-1.357	C	0.053	0.939	1.814
C	0.61	1.738	-0.617	C	-0.513	-1.832	-0.428	C	0.893	1.165	0.891	C	1.801	1.118	-0.025
C	0.876	1.436	0.868	C	1.236	-0.119	-0.25	C	-1.163	-0.05	0.434	C	1.542	1.089	1.49
C	-0.089	0.57	-1.327	C	0.611	-1.145	-1.198	C	-0.615	1.188	1.142	C	1.18	-0.141	-0.633
C	-0.845	-0.341	0.703	C	-1.515	-0.779	0.055	C	1.152	1.156	-0.623	C	-0.472	-0.258	1.024
C	-2.078	-1.072	1.197	C	2.388	0.721	-0.778	C	-2.607	-0.457	0.641	C	-1.949	-0.593	1.151
C	0.353	-1.484	-2.411	C	-1.095	1.854	2.59	C	0.632	-2.108	-2.165	C	1.115	-1.509	-2.515
C	1.279	-2.677	-2.334	C	-1.559	3.292	2.652	C	1.549	-3.304	-2.085	C	0.893	-1.488	-4.012
C	1.068	-3.639	-3.501	C	-1.368	3.866	4.057	C	1.423	-4.167	-3.338	C	0.628	-2.914	-4.502
C	2.043	-4.812	-3.46	C	-1.756	5.34	4.141	C	2.24	-5.451	-3.243	C	0.058	-2.988	-5.916
C	1.921	-5.726	-4.675	C	-1.786	5.863	5.576	C	2.237	-6.251	-4.543	C	-0.319	-4.422	-6.307
C	2.928	-6.874	-4.66	C	-2.05	7.364	5.66	C	3.115	-7.499	-4.485	C	-1.357	-4.509	-7.425
C	2.884	-7.737	-5.921	C	-2.002	7.909	7.088	C	3.151	-8.274	-5.802	C	-1.758	-5.945	-7.762
C	3.883	-8.892	-5.883	C	-2.248	9.415	7.156	C	4.039	-9.514	-5.74	C	-2.882	-6.025	-8.792

References

[1] *Inorganic Chemistry*, **2008**, *57*, 12222–12231.

[2] *Analytical Biochemistry*, **1995**, *231*, 374–382.

[3] a) *Journal of the American Chemical Society,* **2011**, *133*, 3854-3862; b) *Accounts of Chemical Research,* **2013**, *46*, 1020-1028; c) *Journal of Physical Chemistry A*, **2014**, *118*, 6133-6147; d) *Angewandte Chemie International Edition,* **2016**, *55*, 912-916; e) *Angewandte Chemie International Edition*, **2008**, *47*, 3430-3434.

[4] *Nature Chemistry*, **2019**, *11*, 52–56.

[5] *Angewandte Chemie International Edition*, **2002**, *41*, 48–76.
